# Neophobia, Trust, and Perceived Risk in Consumer Responses to Sustainability-Relevant Food Innovations: An Integrative Review and Conceptual Framework

**DOI:** 10.3390/nu18213509

**Published:** 2026-10-25

**Authors:** Sandip Mitra, Sylwia Żakowska-Biemans

**Affiliations:** 1 Department of Functional Foods, Organic Foods and Nutritional Sensory Science, Faculty of Health Sciences, Warsaw University of Life Sciences (SGGW), 02-787 Warsaw, Poland; 2 Department of Agricultural Finance and Cooperatives, Gazipur Agricultural University, Gazipur 1706, Bangladesh

**Keywords:** sustainable foods, food neophobia, food technology neophobia, trust, risk perception, food safety perception, consumer responses, sustainable food innovation

## Abstract

Background/Objectives: Consumer responses to sustainability-relevant food innovations are associated with psychological factors such as food neophobia (FN), food technology neophobia (FTN), trust, perceived risks and benefits, and food-safety-related perceptions. However, these constructs have been examined across heterogeneous contexts, measures, and outcomes. This review aimed to integrate this evidence, examine reported direct and indirect relationships, and develop an evidence-informed conceptual framework. Methods: Five construct-specific searches were conducted in the Web of Science Core Collection and Scopus. Following title/abstract and full-text screening, 288 English-language, peer-reviewed empirical studies with explicit relevance to food-system sustainability were included and synthesised narratively. Results: FN and FTN were generally associated with less favourable consumer responses, although their relative relevance varied with the food, production technology, familiarity, and population. Trust was generally associated with more favourable responses, but associations differed by trust referent and outcome. Innovation-related perceived risk was predominantly associated with less favourable responses, and perceived benefits with more favourable responses. Formal evidence for indirect pathways was limited: only a small number of studies tested FN/FTN- or trust-related indirect relationships through risk and/or benefit perceptions, and their results were heterogeneous. Food-safety-related perceptions, closely related to perceived risk, were operationalised in different ways: safety concerns were generally associated with less favourable responses, whereas perceived safety was associated with more favourable responses. Willingness and behavioural intentions were the most frequently assessed outcomes, whereas few studies assessed behaviour, and evidence from low-income and lower-middle-income countries was limited. Conclusions: Across sustainability-relevant food innovations, consumer responses were generally associated with novelty-related dispositions, referent-specific trust, and parallel risk and benefit appraisals, but the configuration of these relationships varied with the innovation, population, and outcome considered. The evidence is strongest for direct associations with stated responses, whereas indirect pathways and links to behaviour remain less well established. By integrating these constructs within a common framework, this review provides a structured basis for testing their temporal ordering and their relevance to actual purchasing and consumption.

## 1. Introduction

Global food systems contribute considerably to greenhouse gas emissions, land-use change, groundwater depletion, and biodiversity loss, underscoring the urgent need for a transition to more sustainable food systems. Consumer food choices are increasingly recognised as an important component of broader food-system sustainability strategies, given that changes in consumer demand may contribute to shifts towards production and consumption patterns associated with lower environmental pressures [1,2,3]. At the consumer level, this transition entails not only changes in dietary patterns, but also responses to emerging food products and technologies that may contribute to broader sustainability objectives, including the diversification of protein sources, improvements in resource-use efficiency, and the reduction or valorisation of food loss and waste. The realisation of their potential sustainability benefits is contingent, among other factors, on favourable consumer responses to such products and technologies [4,5,6,7,8,9,10].

However, despite growing public awareness of sustainable food choices, an attitude–behaviour gap persists, as favourable attitudes do not consistently translate into actual purchasing and consumption behaviours [3]. Studies combining stated and behavioural measures illustrate this gap: accepting the idea of insects as a meat substitute did not translate into willingness to buy or eat insect-based foods [11], food neophobia reduced self-reported consumption of plant-based meat alternatives and pulses [12], and sustainability attitudes predicted intentions to eat insects but not always actual choices [13]. Behavioural evidence is not uniformly negative, however: a written sustainability passage reduced disgust and maintained actual intake of an insect-containing food [14]. Factors such as food neophobia (FN), food technology neophobia (FTN), trust, risk perception, and food safety perception are frequently associated with consumer responses to novel sustainable foods and food innovations. Among these factors, trust is widely recognised as an important determinant of food-related attitudes and behaviours [3,6,15,16,17]. Current food systems are characterised by increasingly complex supply chains involving producers, processors, retailers, certification bodies, and regulatory agencies [18,19]. As consumers rarely observe food production processes directly, they often rely on trusted actors and certification systems when evaluating food products. Previous studies indicate that trust is associated with food technology acceptance, food safety evaluations, risk perceptions, consumer acceptance of sustainable foods, and emerging food innovations [3,6,19,20,21,22,23]. Trust and risk perception are closely interconnected in food choice research. Several studies suggest that perceived risk and perceived benefits partly explain how trust is associated with purchase-related responses, with the benefit pathway often being the stronger of the two [16,24,25,26,27]. Consumers appear to rely on trust particularly when making judgments about unfamiliar food-related issues [23,28]. In many situations, consumer decisions are guided more by perceived risk than by technical assessments of risk regarding contamination, biotechnology, innovative food-processing technologies, and novel foods [2].

Importantly, perceived risk is multidimensional and encompasses concerns related to health, food safety, environmental impacts, technology, and ethical considerations [29]. Although food safety is assessed scientifically through regulatory and technical measures, consumers often rely on subjective judgments when assessing whether food products are safe for consumption [15,30,31,32,33]. Trust, information exposure, past experiences, and more general public concerns all have an impact on food safety perceptions. Dietary practices, purchase decisions, willingness to pay for food products, and attitudes to food innovations of consumers are influenced by perceptions of food safety [34,35,36,37]. This issue is especially pertinent in low-income and lower-middle-income countries (LICs/LMICs), where food insecurity, weak market regulation, and limited public health infrastructure frequently coexist with underlying concerns about food safety [36,38,39,40]. Research in these settings suggests that basic consumer needs may take priority over sustainability goals [41]. For example, studies from Ghana and Uganda showed that neophobic and socio-cultural barriers, including stigma and social concern, limit the acceptance of insect-based foods [42,43]. For these consumers, cultural familiarity and perceived safety may be stronger drivers of food choice than environmental motives, although this proposition has rarely been tested directly.

Besides trust and perceived risk, consumers’ acceptance of sustainable foods may also be influenced by novelty-related barriers, particularly food neophobia and food technology neophobia. Food neophobia (FN) is the tendency to avoid or reject unfamiliar foods, while food technology neophobia (FTN) refers specifically to reluctance towards foods produced using new technologies [44,45]. Many strategies aimed at improving the sustainability of food systems rely on the development and adoption of innovative food products and technologies, including alternative proteins and cultured meat, food by-products, and other emerging sustainable food innovations [44,46,47]. However, the successful adoption of these innovations ultimately depends on consumer acceptance [48,49,50]. Consumers with high levels of FN and FTN are less likely to embrace novel foods and frequently consider them riskier and less reliable [44,51,52,53,54,55,56,57,58,59,60]. For instance, acceptance of genetically modified foods, food byproducts, alternative protein, and cultured meat has been found to be negatively influenced by food technology neophobia [22,53,60,61,62,63,64,65,66]. These findings indicate that novelty-related barriers are relevant to understanding sustainable food choice, particularly when sustainable options involve novel, unfamiliar products or production technologies [19,28].

Because consumers frequently evaluate sustainable food innovations under conditions of uncertainty, these psychological constructs rarely operate independently. While food neophobia and food technology neophobia typically amplify concerns about unfamiliar technologies, trust serves as a context-dependent mechanism that helps mitigate perceived risk. Specifically, studies on meat substitutes, cultivated meat, and edible insects suggest that higher trust is associated with greater acceptance, partly through risk–benefit evaluations [24,26,27,32]. Conversely, novelty-related barriers generally reduce willingness to try or adopt unfamiliar food technologies [53,55,59,67]. Consumer responses to sustainable food innovations therefore reflect the combined, dynamic influence of trust, risk perception, food safety concerns, and neophobia. These interactions are particularly critical in low-income and lower-middle-income countries (LICs/LMICs), where foundational concerns regarding food safety and institutional trust may precede broader sustainability evaluations [40,42,68].

This review focuses on consumer responses to food innovations that are explicitly relevant to sustainable food-system transitions, particularly where these innovations involve unfamiliar products, production processes, or technologies. Such contexts may involve uncertainty and risk-related evaluations that are particularly relevant to consumer acceptance of emerging food innovations. Accordingly, the review synthesises evidence on food neophobia, food technology neophobia, trust, perceived risk, and food-safety-related perceptions across a range of sustainability-relevant food innovations, including alternative proteins and edible insects, cultivated meat, upcycled foods, and selected gene-based technologies.

Consumer responses are treated as distinct outcomes, including attitudes and acceptance, intentions and willingness, willingness to pay, choice or preference, and reported or observed adoption or consumption. Evidence from low-income and lower-middle-income settings is considered where available to assess the extent to which these relationships vary across socioeconomic and institutional contexts.

## 2. Materials and Methods

### 2.1. Review Design

An integrative review design was employed, supported by a structured literature search, screening procedures, standardised data extraction, and evidence synthesis [69,70,71,72,73]. This approach was considered appropriate because the literature includes heterogeneous research designs and conceptual approaches examining psychological drivers [70,73].

### 2.2. Search Strategy

Literature retrieval was conducted through the Web of Science Core Collection and Scopus databases, which index journals relevant to food science, consumer studies, sustainability, nutrition, and public health. The final database searches were conducted on 2 September 2026. No restrictions were imposed on publication year in order to include both earlier foundational studies and more recent research. Because the psychological constructs examined in this review (food neophobia, food technology neophobia, trust, risk perception) have frequently been investigated independently, five separate construct-specific searches were conducted. This ensured that literature retrieval did not require the simultaneous presence of all focal constructs alongside sustainability terms. Each construct-specific search combined the relevant construct terms with a common food-related search block. This block included two complementary groups of terms: broad sustainability-related concepts (e.g., sustainable food, sustainable diets, sustainable food choice) and terms referring to food products and technologies frequently examined in relation to sustainable food-system transitions (e.g., alternative proteins, cultivated meat, cellular agriculture, plant-based alternatives). The inclusion of these innovation-related terms did not imply that all such products or technologies were assumed to be inherently sustainable. Rather, these categories were included because the focal constructs are particularly relevant to consumer responses to unfamiliar foods and emerging food technologies.

The strategy was not intended to provide exhaustive coverage of every category commonly associated with sustainable food consumption. Accordingly, categories such as organic food were not included as separate search terms, although studies on organic foods were retained when retrieved through the broader sustainability-related terms. The complete search strategies for Web of Science and Scopus are provided in Supplementary Table S1.

### 2.3. Eligibility Criteria and Study Selection

Eligible studies were peer-reviewed empirical journal articles published in English that met all of the following criteria: (1) involved consumers evaluating foods, food products, or technologies directly related to food production or processing, or making food-related choice, purchase, or consumption decisions; (2) assessed at least one focal construct, namely food neophobia, food technology neophobia, trust, perceived risk, or food-safety-related perceptions; (3) measured at least one distinct consumer-response outcome, including attitudes or acceptance, intentions or willingness, willingness to pay, choice or preference, or reported or observed adoption or consumption; and (4) demonstrated explicit relevance to food-system sustainability.

The sustainability-relevance criterion was strictly defined: the focal food, technology, or consumer behaviour had to be explicitly examined in relation to at least one food-system sustainability objective, such as reducing environmental impacts, mitigating food waste, or supporting alternative-protein transitions. Novelty or technological innovation alone was not considered sufficient evidence of sustainability relevance. For example, a study examining consumer acceptance of gene-edited food solely because it represents a novel technology was excluded, whereas an investigation of gene-edited crops aimed at reducing agricultural inputs was included.

**Figure 1:**
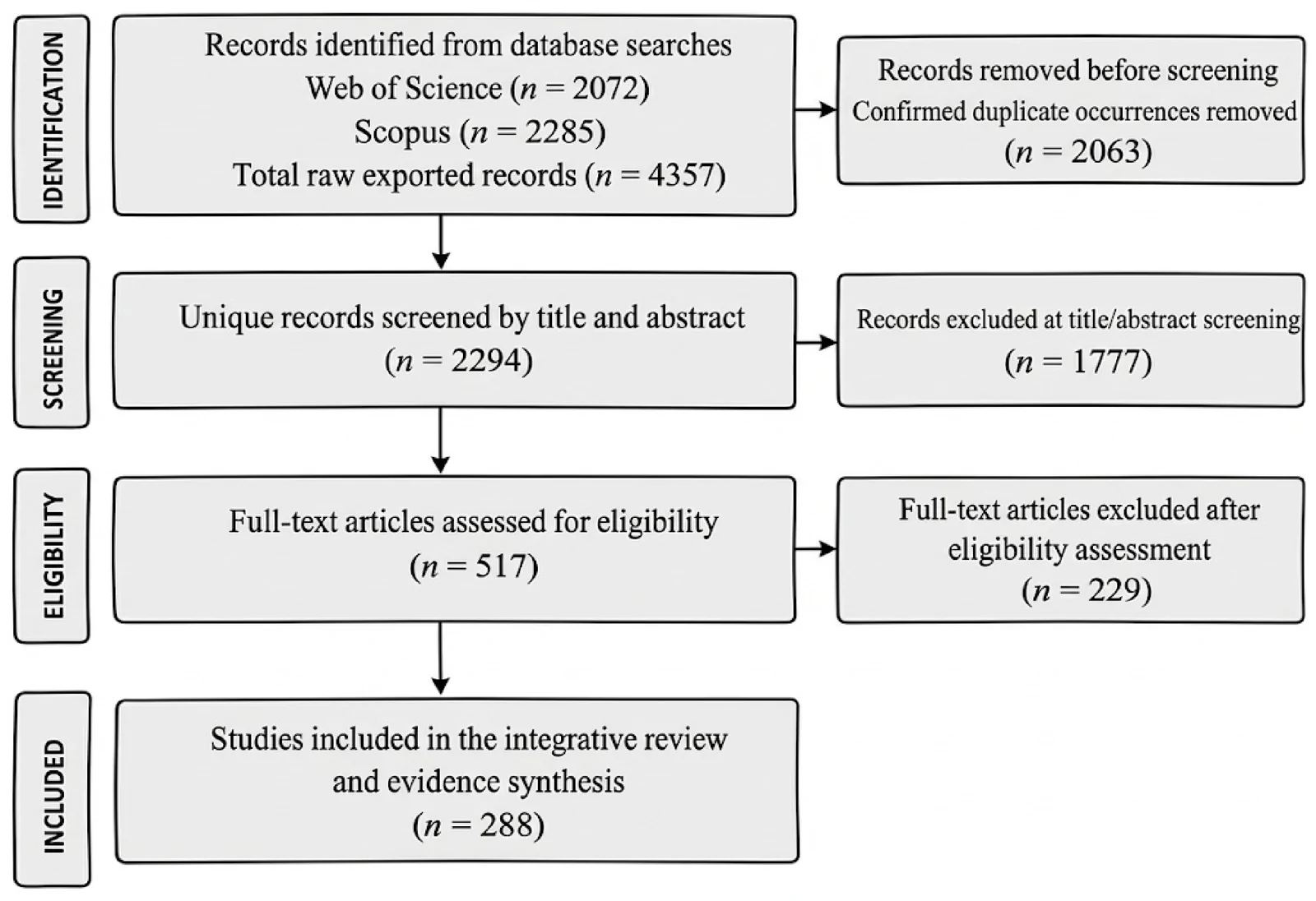
Flow diagram of the literature search and study selection process.

Study selection followed a sequential two-stage process (Figure 1). Following duplicate removal, records underwent title and abstract screening. To maximize sensitivity at this initial stage, screening was broad, and studies were not excluded solely because explicit sustainability terminology was absent from the abstract. The strict sustainability-relevance criterion was subsequently applied during the full-text eligibility assessment, where the substantive context of the research could be accurately verified. Studies lacking accessible full texts were excluded because methodological eligibility could not be confirmed. The screening process was conducted independently by the authors, with disagreements resolved through consensus. A total of 288 eligible studies were included in the final evidence synthesis. Finally, country income was not used as an eligibility restriction. Instead, after inclusion, study locations were classified according to the World Bank income classification to enable contextual comparisons of the evidence between high-income settings and low-income or lower-middle-income settings [74,75].

### 2.4. Data Extraction and Analysis

A standardised literature extraction matrix was used to extract data from all included studies [76]. The extracted information was checked by the authors to ensure consistency and completeness of data before narrative synthesis. The matrix captured study characteristics, population and setting, study design and measurement approaches, food/product context, psychological constructs, consumer response outcomes, explicit sustainability relevance, and major findings (Supplementary Table S2). The extracted information was used to assess the consistency of relationships identified among the focal constructs and consumer response outcomes. For each included study, evidence was categorised according to the construct examined and the consumer response outcome (e.g., acceptance, willingness to try, purchase intention, willingness to pay, or actual adoption). This approach enabled comparison of findings across heterogeneous study designs and facilitated the identification of consistently supported, mixed, or context-dependent, and comparatively limited evidence. Narrative synthesis was conducted across the extracted evidence to identify recurring patterns and conceptual relationships among the focal constructs and consumer response outcomes. Considering the integrative review design and the inclusion of heterogeneous quantitative and qualitative evidence, a formal risk-of-bias assessment was not conducted.

### 2.5. Development of the Integrated Conceptual Framework

Findings from the narrative synthesis were subsequently combined to construct the proposed conceptual framework [77]. The framework synthesises recurring direct associations, mixed or context-dependent findings, and formally tested mediation pathways identified across the included evidence base. It was developed iteratively from the synthesised evidence and is presented in Section 3.5.

## 3. Results

### 3.1. Characteristics and Overview of Included Studies

A total of 288 studies met the final eligibility criteria and were included in the review (Supplementary Table S2). Detailed study-level characteristics—including geographical settings, study designs, food and technology contexts, focal psychological constructs, consumer-response outcomes, sustainability relevance and rationale, and main findings and framework relevance—are provided in Supplementary Table S2. Because individual studies could examine more than one focal construct, construct categories were non-mutually exclusive. Food neophobia was examined in 160 studies, food technology neophobia in 49 studies, trust in 80 studies, focal food-, innovation-, or process-related perceived risk in 48 studies, and food-safety-related perceptions in 35 studies. A total of 21 studies included participants from at least one low-income or lower-middle-income country. The evidence encompassed a wide range of novel or technologically unfamiliar foods and sustainability-relevant food innovations, including cultivated meat, plant-based alternatives, edible insects, precision fermentation, upcycled or side-stream foods, algae- and seaweed-based products, and other emerging food technologies.

Consumer-response outcomes also varied considerably in their proximity to behaviour. To comprehensively capture consumer responses, outcomes were classified into five broad categories. Because several studies evaluated multiple dependent variables simultaneously, this classification applies to all individual measured outcomes rather than assigning a single primary outcome per study. Consequently, the categories are not mutually exclusive. The distribution of measured outcomes across the reviewed literature is as follows: Behavioural Intentions, including willingness to buy or consume (*n* = 170); Attitudes, Acceptance, and Hedonic Liking (*n* = 121); Behaviourally Anchored Outcomes, including self-reported consumption and choice experiments (*n* = 49); Willingness to Try or Taste (*n* = 45); and Willingness to Pay (*n* = 24). Given the predominance of intention-based outcomes, these categories were treated as distinct endpoints rather than as sequential stages in a behavioural process.

### 3.2. Food Neophobia and Food Technology Neophobia in Consumer Responses to Novel and Sustainability-Relevant Food Innovations

Food neophobia (FN) and food technology neophobia (FTN) represent related but conceptually distinct novelty-related dispositions. FN refers to reluctance to eat or try unfamiliar foods [45], whereas FTN concerns reluctance towards foods produced using unfamiliar or novel technologies [44]. This distinction is particularly relevant to sustainability-oriented food innovations, many of which involve novelty in the food itself, the production process, or both. Across the reviewed evidence, higher FN and FTN were generally associated with less favourable consumer responses, although the strength of these associations varied across food and technology contexts (Table 1 and Supplementary Table S2).

The evidence suggests that FN and FTN should not be regarded as interchangeable manifestations of a single novelty-related barrier. Their relative importance appears to depend on the source of unfamiliarity. For example, Hwang et al. [61] found that food neophobia was negatively associated with willingness to buy cultivated meat but was not significantly associated with willingness to buy plant-based meat alternatives, indicating that the relevance of general food neophobia may vary even across products positioned within the same alternative-protein transition. Similarly, Peressi and Simonetti [78] found that food technology neophobia (FTN), but not general food neophobia (FN), was associated with lower expected sensory quality and, indirectly, with a lower likelihood of choosing precision-fermentation dairy. This suggests that when a familiar food is made using a novel method, consumer reluctance may be more closely related to the unfamiliar production process than to the food itself, with sensory expectations forming part of this association. Furthermore, Kamei et al. [79] reported that higher food neophobia was associated with lower willingness to eat foods containing several alternative protein sources, with stronger negative associations for less familiar foods. Cross-national comparisons further indicate contextual variation: food technology neophobia was the key driver of rejection of a novel yoghurt in Greece and Cyprus, whereas food neophobia was more influential in Uganda [80]. Together, these findings suggest that familiarity and context may condition the association between neophobia and consumer responses.

FTN is particularly relevant when acceptance depends on consumers’ evaluations of unfamiliar production technologies. Negative associations between FTN and consumer responses have been reported for genetically modified foods, cultivated meat, precision-fermentation products, and other technology-intensive food innovations [60,78,81]. In genetically modified foods, FTN has been associated with less favourable attitudes and lower purchase or consumption intentions, while studies of cultivated meat similarly identify technology-related reluctance as an important barrier to acceptance. However, the strength of these associations varies across contexts, and consumer evaluations of unfamiliar food technologies also involve factors such as expected taste, perceived naturalness, past experience, and perceived benefits.

The available evidence further highlights those neophobic responses vary depending on the specific product and population context. For example, Dupont et al. [82] demonstrated that FN and FTN can differ in their relative importance even within the same cultivated-meat context. Exploring cross-category differences, Etter et al. [83] found that while FN was associated with the acceptance of plant-based proteins, algae, and insects, consumer responses to cultivated meat showed a different pattern where a preference for naturalness was particularly relevant. This contextual heterogeneity is also evident across populations; Boereboom et al. [63] demonstrated the simultaneous relevance of FN and FTN across different consumer segments and countries for cultivated meat, while Giacalone and Jaeger [84] provided broader support for cross-national heterogeneity across various sustainable food technologies. Together, these studies indicate that FN and FTN are context-dependent and non-equivalent influences, rather than universal barriers operating identically across all foods and technologies.

The reviewed evidence also cautions against assuming that information provision alone is sufficient to overcome novelty-related reluctance. Among Danish children aged 9–13 years, Erhard et al. [85] found that communicating information about the taste, health, or sustainability benefits of insect-based foods did not increase willingness to try them, while food neophobia remained a strong negative predictor of willingness. Similarly, an educational intervention with German adolescents reported by Szczepanski et al. [86] reduced FN, FTN, and food disgust and improved knowledge and attitudes towards insect-based foods, but did not significantly increase willingness to consume them. In some cases, information may have unintended effects: among highly neophobic Italian respondents, information about edible insects increased food neophobia [87], whereas in Spain health- and environment-related information did not increase acceptance of mycoprotein on its own but moderated the negative effect of FTN [88]. These findings suggest that changes in knowledge or novelty-related evaluations do not necessarily translate into corresponding changes in consumer intentions. In contrast to passive information provision, direct sensory experience may also modify consumer responses; for example, Barton et al. [89] found that direct tasting reduced disgust and increased willingness to buy and recommend cricket protein powder, and direct consumption of black soldier fly products reduced disgust among rural consumers in Madagascar [90].

Neophobia also occurs alongside affective and evaluative responses such as disgust, perceived unnaturalness, perceived risk, and perceived benefits. For instance, Szlachciuk and Żakowska-Biemans [91] found that neophobic consumers experienced greater disgust and perceived higher risks associated with insect-based foods, whereas more neophilic consumers focused on the benefits. Disgust and perceived unnaturalness repeatedly emerged as important correlates of consumer responses: they were important predictors of cultured-meat acceptance across ten countries [92], state disgust statistically mediated the association between trait disgust sensitivity and the acceptance of new food technologies [93], and disgust was a considerably stronger predictor than neophobia of the intention to eat insect-based food [94]. While these findings highlight a strong connection, cross-sectional observations of this kind cannot establish a cause-and-effect chain. When researchers formally test indirect pathways, they find a mixed picture, showing that perceived risk is not a universal mediator. Cascone et al. [17] found that the association between neophobia and lower intentions to consume insect protein included indirect pathways through lower perceived benefits and higher perceived risks, with the benefit pathway being stronger. Perceived risks and benefits also mediated the effects of food technophobia on four types of alternative protein [81]. On the other hand, Baum et al. [25] found that for CRISPR/gene-edited foods, perceived benefits were more strongly associated with behavioural intentions than perceived risks, whose association with intentions was not significant, and Karakuş et al. [95] found no significant mediating role of food neophobia. These findings indicate that associations between neophobia and consumer responses may involve affective responses, expected benefits, perceived risks, and sensory expectations, rather than a single, predictable risk pathway. Although FN and FTN are generally treated as relatively stable dispositions, their expression and associations with consumer responses may vary across contexts. Differences in prior experience, cultural exposure, and cognitive orientation may influence the extent to which novelty generates avoidance. For example, Dai et al. [96] found that multicultural experience was associated with lower technology neophobia via a lower need for cognitive closure, and with greater acceptance of novel food technologies via higher openness to experience. This suggests that the expression of technology-related neophobia can vary with consumers’ previous experiences and the cognitive context in which novel technologies are evaluated.

Overall, the evidence shows that general FN and FTN are connected but distinct. FN appears particularly relevant when the food itself is unfamiliar, whereas FTN may become more relevant when concerns centre on the technological means of production. Their associations with consumer responses also vary with familiarity, cultural context, sensory expectations, perceived benefits, and risk-related evaluations. Direct associations with attitudes, acceptance, willingness, and purchase-related outcomes are more consistently documented than specific indirect pathways through risk and benefit perceptions, and null effects of neophobia were also reported for plant-based meat alternatives, microalgae, and cultivated meat [61,97,98]. Neophobia should therefore not be conceptualised as a uniform barrier operating through a single mechanism, but as a context-dependent influence on consumer responses to novel and sustainability-relevant food innovations.

### 3.3. Trust in Consumer Responses to Novel and Sustainability-Relevant Food Innovations

Across the reviewed studies, trust was not operationalised as a single homogeneous construct. Rather, trust referred to consumers’ confidence in specific actors and systems across the food supply chain, including regulatory authorities, scientists, food manufacturers, information sources, and the technologies themselves. For example, Harrison and Liem [27] distinguished interpersonal trust, institutional trust in authorities, epistemic trust in cultivated-meat science, and trust in technology oversight. Wendt and Weinrich [99] examined four dimensions of trust: interpersonal trust, and trust in the European Food Safety Authority (EFSA), the industry, and the product, while Pareti et al. [26] separated trust in manufacturers, regulatory agencies, media, and traceability systems. Furthermore, Vanutelli et al. [100] specifically examined trust in science, Chen [55] examined supply-chain trust, and Chang et al. [101] explicitly focused on technological trust. Accordingly, findings concerning trust should be interpreted in relation to the specific dimension of trust examined rather than as evidence concerning a single generalised trust construct.

Cultivated meat provides substantial evidence that trust is relevant when consumers evaluate technologically unfamiliar foods. Across studies, greater trust in regulatory authorities, scientists, food-system actors, or technology oversight was generally associated with more favourable attitudes or willingness to try or purchase cultivated meat, whereas distrust was associated with less favourable responses. However, the relative importance of particular trust domains varied across studies and national contexts. For instance, Harrison and Liem [27] demonstrated that epistemic trust, institutional trust, and trust in technology oversight were associated with cultivated-meat acceptance, and that perceived risks and benefits mediated these associations. Similarly, Muiruri and Rickertsen [32] found that higher social trust and trust in food authorities were associated with greater willingness to try cultivated meat. Beyond regulators, trust in commercial actors may also be relevant; for example, participants in the qualitative study by Hanan et al. [66] described mistrust of large corporations, alongside unfamiliarity and food neophobia, as a barrier to cultivated-meat acceptance.

Trust and technological reluctance frequently coexist in different consumer configurations. Using latent profile analysis, Wendt and Weinrich [99] found that a “Trusting Consumers” profile exhibited greater cultivated-meat acceptance and lower food technology neophobia (FTN), while “Distrustful Consumers” showed the reverse pattern. A similar pattern is evident across countries. Specifically, Loera et al. [102] reported that across Italy, France, and the Netherlands, FTN and distrust in food-safety authorities were among the most consistent barriers to cultivated-meat acceptance. Importantly, trust-related barriers appear to be technology-specific and should not be assumed to transfer uniformly across different alternative-protein categories. Hwang et al. [61] highlighted this technology-specific distrust: distrust of biotechnology negatively affected willingness to buy cultured meat, whereas the pattern differed for plant-based alternatives. Referent-specific effects can even be opposite in sign: in rice choice experiments in China and Thailand, abstract trust in institutions reinforced the status quo, whereas interpersonal trust in farmers was associated with greater switching to sustainable alternatives [103].

Evidence from precision fermentation similarly suggests that trust in the manufacturer is closely tied to consumers’ overall views of the technology: greater trust was associated with higher perceived benefits and lower scepticism and neophobia, while perceived usefulness was associated with attitudes and purchase intentions [104].

In insect-based foods, trust appears particularly relevant in conjunction with food-safety-related concerns. Muiruri [16] found that social trust and trust in food authorities were positively associated with willingness to try insect foods, whereas food-safety concerns were negatively associated with willingness and contributed to an indirect pathway. Trust in science was also associated with differences in consumer profiles. Vanutelli et al. [100] identified consumer profiles in which higher trust in science co-occurred with more favourable attitudes and intentions towards insect-based foods, whereas higher neophobia and lower scientific trust characterised a more resistant profile. Trust in information channels also shapes consumer responses, although source preferences may vary across contexts. Legendre et al. [105] observed that familiarity with edible insect foods was positively associated with media trust, which in turn was linked to purchase activism and to purchase intention and expected liking of edible insect products. A different pattern was observed among rural Kenyan consumers, where industry and policy sources were trusted more than media [106]. In that setting, familiarity and perceived behavioural control were stronger predictors of intention, indicating that trust should not be interpreted as the dominant predictor of consumer response.

In plant-based alternatives, trust may also concern food manufacturers and supply-chain partners. For example, Chen [55] found that supply-chain trust was positively associated with consumers’ willingness to try plant-based meat. Beyond actors and supply chains, trust is also directed toward the technology itself. In the case of plant-based seafood, technological trust was directly associated with purchase and advocacy intentions, illustrating a technology-specific form of trust distinct from institutional or interpersonal trust [101].

Higher trust was generally associated with lower perceived risk and higher perceived benefits in studies that examined these relationships. Harrison and Liem [27] found that perceived benefits and risks mediated associations between several trust domains and cultivated-meat acceptance, whereas Pareti et al. [26] showed that greater consumer trust was associated with higher perceived benefits and lower perceived risks, both of which partially mediated willingness to purchase meat substitutes. Notably, benefit-related indirect effects were substantial, suggesting that the role of trust should not be conceptualised exclusively in terms of risk reduction. Similar patterns were reported for 3D-printed food, where trust in institutions, perceived benefits, and perceived risks jointly predicted consumption intention [107], and for cultured meat, where information about scientists’ financial motives weakened the effect of scientific consensus on safety perceptions by undermining trust in scientists [108]. Trust weakened the negative association between perceived risk and attitudes towards upcycled food [109] and strengthened the association between motivation and intention for sustainable and innovative food choices [3]. The evidence therefore supports the presence of indirect associations involving trust, perceived risks, and perceived benefits in some novel-food contexts, but does not establish a universal pathway across technologies.

Trust is also related to, but conceptually distinct from, food technology neophobia. Lower trust and higher neophobia frequently co-occur; for instance, Wendt and Weinrich [99] identified distinct consumer profiles where trust mirrored acceptance and neophobia levels, and trust in the manufacturer was associated with lower neophobia towards precision fermentation [104]. However, existing data do not establish whether distrust increases neophobia or whether neophobic consumers are more likely to report lower trust. Trust and neophobia should therefore be conceptualised as distinct, interrelated factors rather than components of a fixed causal sequence.

Importantly, most evidence linking trust to consumer responses focuses on attitudes and intentions rather than actual consumption. Exceptions include cross-lagged panel evidence that trust predicts the use of sustainable seafood labels [110] and a population-scale study linking trust to purchases of local and organic products [111]. The broader literature, however, predominantly maps psychological states rather than behavioural outcomes, limiting conclusions about the extent to which trust translates into food-related behaviour.

While trust mechanisms also appear in established sustainability contexts, such as the use of sustainable seafood and eco-labels [110,112], they are also evident when consumers evaluate novel, unfamiliar food technologies. However, trust is not a uniform predictor; its associations with consumer responses vary across trust referents, technologies, populations, and national contexts [61,102,106,113]. Rather than acting as a universal determinant, trust operates through direct associations with consumer responses, such as trust in authorities, supply chains, or the technology itself [32,55,101], and through indirect mechanisms involving risk–benefit evaluations [26,27]. Trust also frequently co-occurs with food technology neophobia, though the causal direction between them remains unclear. Ultimately, trust is best conceptualised as a relational, referent-specific, and context-dependent construct rather than a standalone driver of novel-food acceptance.

### 3.4. Risk Perception in Consumer Responses to Novel and Sustainability-Relevant Food Innovations 

Perceived risk refers to consumers’ subjective evaluations of the potential adverse consequences associated with a food, technology, or production system and should be distinguished from technical or expert assessments of risk [2,114]. Across the reviewed evidence, perceived risk was generally associated with less favourable consumer responses to sustainability-relevant food innovations, although the magnitude and nature of this association varied across products, risk dimensions, and consumer contexts. Importantly, risk was not evaluated in isolation: studies frequently examined it alongside perceived benefits, trust, knowledge, and novelty-related responses.

The reviewed evidence does not support treating perceived risk as an absolute barrier to acceptance. Rather, consumer responses appear to reflect a risk–benefit evaluation in which the relative weight of potential disadvantages and advantages varies across contexts. When looking at upcycled foods, Mao et al. [109] showed that perceived risks negatively affected consumer attitudes, whereas perceived benefits exerted a stronger positive association. Additionally, higher trust and better knowledge successfully moderated and weakened the negative association between perceived risk and attitudes. Similarly, in insect-based protein supplements, perceived benefits were more strongly associated with consumption intention than perceived risks [17], and perceived value exerted a much stronger influence than perceived risk on attitudes towards cultivated meat and algae-based products [115]. These findings are consistent with the broader risk–benefit perspective on food-technology acceptance [116].

A key distinction is where consumers locate the risk. When consumers view a novel food as risky, they tend to reject it [117,118,119]. Conversely, perceiving risks in the conventional food system can make sustainable alternatives more attractive. For instance, climate-change risk perception was positively associated with optimism towards artificial meat and edible insects [95], and health and environmental risk perceptions of ultra-processed foods were associated with more favourable attitudes towards reducing their intake [120]. This means that risk can either hinder or support the adoption of new diets, depending on whether the threat is attributed to the new technology or to the conventional food it replaces. Evidence for this second pathway, however, remains thin.

Perceived risk also emerged as an intermediate evaluative mechanism linking other focal constructs with consumer responses. Trust in regulators was associated with lower perceived risk and, indirectly, with stronger purchase-related responses towards edible-insect foods [24]. In cultivated meat, Harrison and Liem [27] found that perceived risks and benefits mediated relationships between several dimensions of trust and acceptance, with benefit-related indirect effects generally stronger than risk-related ones. Similarly, Pareti et al. [26] reported that trust was associated with lower perceived risks and greater perceived benefits, both of which partially mediated acceptance of meat substitutes.

Risk-related pathways were also identified for novelty-related constructs. In insect-based protein supplements, FN and FTN were indirectly associated with lower consumption intention through greater perceived risk and lower perceived benefits [17]. In genetically modified foods, higher FTN was associated with greater perceived risk and lower perceived benefits, which in turn shaped attitudes and purchase intentions [60]. Ma et al. [81] observed a similar pattern for four types of alternative protein, and the visual presentation of insects increased risk perceptions and reduced purchase intent [121]. Ultimately, these findings justify including perceived risk, together with perceived benefits, as an intermediate appraisal in the proposed framework, although only a small number of studies have formally tested these indirect pathways and null findings were also reported [95,122,123].

Therefore, perceived risk should not be treated as a single, generic concept. Depending on the study, researchers defined risk in terms of health, safety, functional performance, financial cost, social, environmental, or broader technological concerns [119]. Because the importance of these specific concerns varies by product, combining all risks into one general category might obscure what consumers actually care about.

#### Food-Safety-Related Risk and Safety Perceptions

Rather than treating food-safety-related perceptions as a completely separate psychological mechanism, they are considered here as a specific dimension of perceived risk and safety evaluation. The challenge in the literature is that researchers measure this domain from two opposite angles. Some focus on positively framed perceptions, such as perceived safety, while others assess concerns about contamination, adverse health effects, or other food-safety risks. Consequently, the direction of reported associations partly depends on how the construct is operationalised: higher perceived safety is generally associated with more favourable consumer responses, whereas greater food-safety concerns are generally associated with less favourable responses. The clearest connection between food safety and other psychological factors lies in its relationship with trust. For example, Muiruri [16] found that social trust and trust in food authorities were positively associated with willingness to try insect-based foods, and that food-safety concerns statistically mediated the association between trust and willingness. Similarly, scientific consensus on the safety of cultured meat increased perceived safety unless trust in scientists was undermined [108], and higher trust in national regulators was associated with greater perceived safety of precision-fermentation ingredients in infant formula [124]. This suggests that safety concerns do not just act as a direct barrier; they are deeply tied to how much consumers trust the system producing and regulating the food.

However, the broader evidence is heterogeneous and does not indicate that food-safety concerns consistently outweigh other determinants across products or contexts. Furthermore, research from outside predominantly Western countries remains limited. For example, food safety was among the determinants of purchase intentions for alternative foods among Chinese college students [125]. Among young urban adults in Chad, affective risk barriers were the strongest predictors of cultured-meat adoption intention, yet about half of respondents were willing to try it [126]. These findings suggest that safety- and risk-related evaluations may be relevant to acceptance in such settings, although the available evidence remains limited. The available evidence is insufficient to determine whether these relationships differ systematically across country-income settings.

### 3.5. An Integrated Framework of Consumer Responses to Sustainability-Relevant Food Innovations

Table 1 summarises the evidence across the 288 included studies informing the relationships represented in the conceptual framework. Importantly, the proposed framework should be interpreted as an evidence-informed conceptual model rather than a causal model. The relationships represented in the framework were identified across heterogeneous observational and experimental studies. Where mediation pathways were reported, only studies that formally tested indirect effects were considered as evidence for mediation, whereas other relationships were interpreted as associations.

The evidence summarised in Table 1 indicates that trust was generally positively associated with consumer responses, although associations varied by trust referent, whereas FN, FTN, and innovation-related perceived risk were predominantly negatively associated with consumer responses. Perceived benefits were generally positively associated with consumer responses.

Evidence for indirect pathways was comparatively limited. Three included studies formally tested indirect relationships linking FN/FTN, perceived risk and/or perceived benefits, and consumer responses [17,25,81]. These studies did not support a single uniform pathway: the relative contribution of perceived risk and benefits varied across models, and benefit-related pathways were particularly prominent in some contexts. Four studies formally tested indirect relationships linking trust, perceived risk and/or perceived benefits, and consumer responses [24,25,26,27]. Across these models, higher trust was associated with lower perceived risk and/or higher perceived benefits, although the relative contribution of these appraisal pathways varied by trust referent and food-innovation context. In Baum et al. [25] and Harrison and Liem [27], benefit-related pathways were more prominent than risk-related pathways.

**Figure 2:**
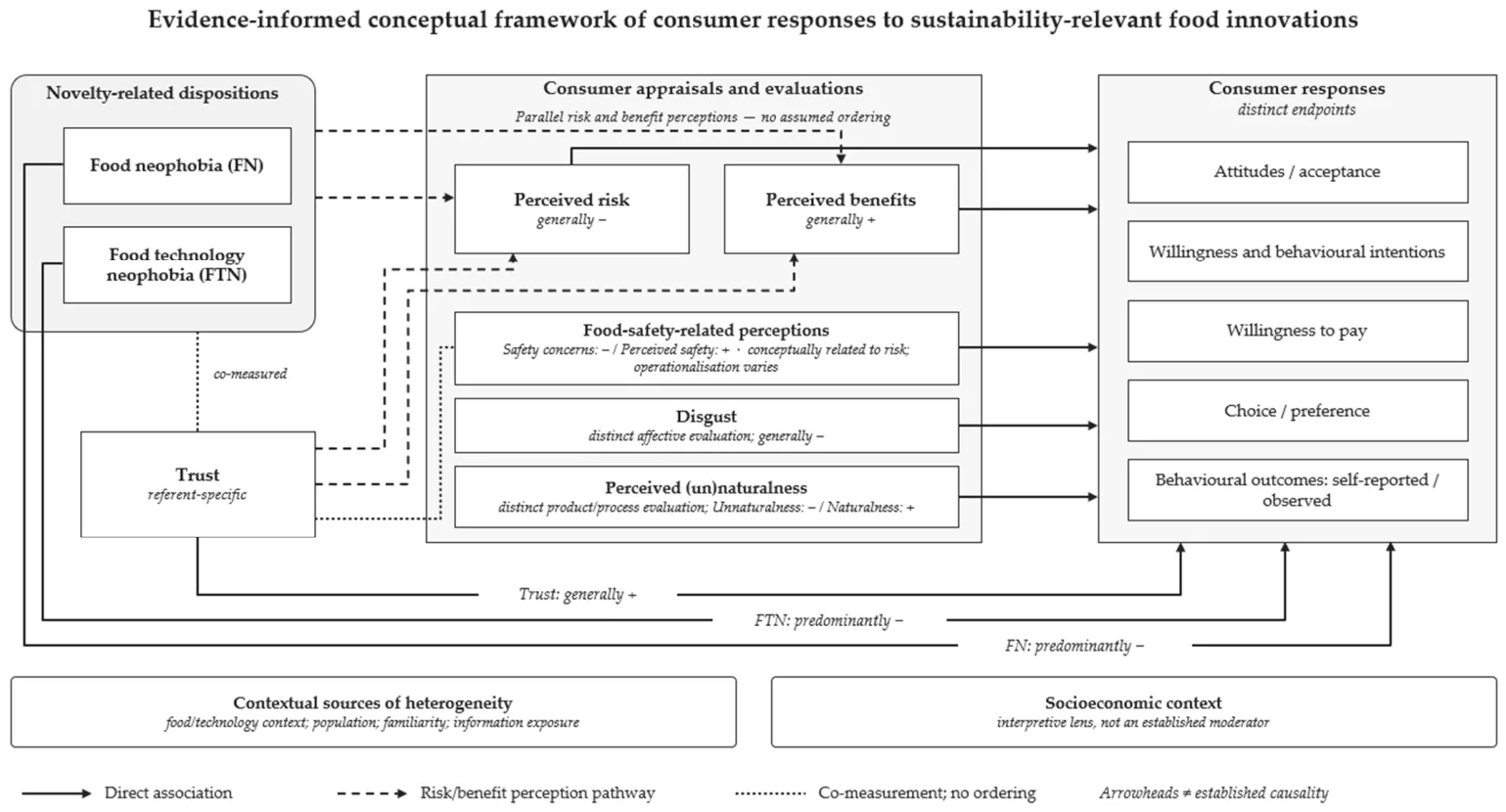
Evidence-informed integrated conceptual framework of consumer responses to sustainability-relevant food innovations. Note: The framework provides a conceptual organisation of the reviewed evidence and should not be interpreted as a causal model. Solid lines represent direct associations reported in the reviewed studies. Dashed lines represent risk- and benefit-related appraisal pathways; formal indirect effects were tested only in a subset of studies. Dotted lines indicate co-measurement or associations for which the direction or temporal ordering remains unresolved. Arrowheads indicate the conceptual direction represented in the framework and do not imply established causality. Consumer-response outcomes are distinct endpoints rather than sequential stages of adoption. Contextual characteristics are represented as potential sources of heterogeneity, while socioeconomic context is treated as an interpretive lens.

Consumer responses were represented as distinct endpoints comprising attitudes or acceptance, willingness or behavioural intentions, willingness to pay, choice or preference, and behavioural outcomes. Willingness and behavioural intentions constituted the most frequently assessed outcome category, whereas a limited number of studies included behavioural measures. Figure 2 provides a conceptual organisation of the reviewed evidence, distinguishing direct associations, risk- and benefit-related appraisal pathways, and co-measurement or associations for which the direction or temporal ordering remains unresolved.

**Table 1:** Evidence informing the relationships represented in the conceptual framework (Figure 2).

Relationship Represented in Figure 2	Observed Pattern	Evidence Basis/Illustrative Studies	Key Interpretive Qualification
FN → consumer responses	Predominantly negative, but context-dependent	Direct associations. Bryant et al. [127]; Hwang et al. [61]; Bahraseman et al. [128]; Karakuş et al. [95]; Kasana et al. [97]; Annunziata et al. [98]; Spendrup and Hovmalm [12].	Associations vary by food context, familiarity, population, and outcome.
FTN → consumer responses	Predominantly negative, but context-dependent	Direct associations and structural models. Baum et al. [129]; Lanz et al. [130]; Modlinska et al. [131]; Fantechi et al. [132]; Lührmann et al. [133]; Pawelczyk et al. [88]; Tsimitri et al. [80]; Peressi and Simonetti [78].	FTN is distinct from FN and appears particularly relevant when the production technology is unfamiliar.
Trust → consumer responses	Generally positive, with variation by trust referent and outcome	Direct associations, structural models, and profile analyses. Harrison and Liem [27]; Wendt and Weinrich [99]; Chen [55].	Trust is referent-specific; evidence is concentrated on stated responses rather than behaviour.
Risk and benefit appraisals → consumer responses	Higher perceived risk is generally associated with less favourable responses, whereas perceived benefits are generally associated with more favourable responses	Associations and structural models. Baum et al. [25]; Mao et al. [109]; Ma et al. [81]; Pareti et al. [26].	Risk and benefits are parallel appraisals. Benefits were more influential than risk in some models, but this is not a universal pattern.
Food-safety-related perceptions → consumer responses	Safety concerns are generally associated with less favourable responses; perceived safety with more favourable responses	Associations and selected mediation/choice models. Muiruri [16]; Pittman et al. [33]; Yang et al. [125].	Safety concerns and perceived product safety are different operationalisations and should not be treated as interchangeable.
Disgust → consumer responses	Generally negative	Direct associations and selected affective-pathway tests. La Barbera et al. [94]; Egolf et al. [93]; Siegrist and Hartmann [92].	Disgust is distinct from FN and perceived risk; temporal ordering relative to other evaluations is unresolved.
Perceived (un)naturalness → consumer responses	Greater perceived unnaturalness is generally associated with less favourable responses	Associations and framing evidence. Siegrist and Hartmann [92]; Etter et al. [83]; Banovic and Grunert [104].	Measured perceived naturalness and experimentally manipulated naturalness framing should be distinguished.
FN/FTN → risk/benefit appraisals → consumer responses	Formal indirect effects reported in a limited evidence base	All identified formal tests: Baum et al. [25]; Ma et al. [81]; Cascone et al. [17].	Evidence supports heterogeneous risk- and benefit-related pathways rather than a single sequential mechanism.
Trust → risk/benefit appraisals → consumer responses	Formal indirect effects reported in a limited evidence base	All identified formal tests: Baum et al. [25]; Legendre and Baker [24]; Pareti et al. [26]; Harrison and Liem [27].	Relative contributions of risk and benefits vary by model and trust referent; cross-sectional mediation does not establish temporal ordering.
Trust ↔ FN/FTN	Associations reported in some studies; ordering unresolved	Co-measurement and correlational/profile evidence. Banovic and Grunert [104]; Wendt and Weinrich [99]; Vanutelli et al. [100].	Co-measurement alone does not establish an association, and the dotted link should not be interpreted as a reciprocal effect.
Trust ↔ food-safety-related perceptions	Context- and referent-specific associations	Associational, mediation, and experimental evidence. Muiruri [16]; Eom and Choy [108]; Mankad and Carter [124].	Direction depends on trust referent, safety operationalisation, and study design; causal ordering is unresolved.

Note: FN, food neophobia; FTN, food technology neophobia. Relationships are presented for conceptual organisation and do not imply causality. Illustrative studies were selected to reflect the observed pattern and relevant contextual or methodological variation; where only a small number of studies formally tested an indirect pathway, all identified studies are reported. Consumer-response outcomes are treated as distinct endpoints.

## 4. Discussion

### 4.1. Discussion and Implications of the Integrated Framework

Research on sustainability-relevant food innovations has examined FN, FTN, trust, perceived risk, perceived benefits, and food-safety-related perceptions across diverse products and technologies. Studies combining these constructs differ substantially in their trust referents, appraisal measures, food-innovation contexts, and consumer-response outcomes [24,25,26,27]. The contribution of this review therefore lies not primarily in identifying individual associations that are already well established, but in integrating these research streams and clarifying distinctions between novelty-related dispositions, trust, appraisal domains, and consumer-response outcomes. The resulting framework should be interpreted as an evidence-informed conceptual organisation of the available literature rather than as a universal causal model. Trust emerged as an important but strongly referent-specific construct. The reviewed studies examined trust in different actors and systems, including food authorities, scientists, producers, retailers, technology oversight, and information sources, and these forms of trust were not uniformly associated with consumer responses. Overall, higher trust was generally associated with more favourable responses, but the strength and significance of associations depended on the trust referent and the outcome examined. Formal indirect-effect models further indicate that trust may be associated with consumer responses through perceived risks and benefits, but these pathways were tested in only a small subset of studies. Four included studies formally examined trust-related indirect relationships involving risk and/or benefit perceptions and consumer responses [24,25,26,27]. Their findings support the inclusion of risk and benefits as relevant appraisal dimensions, but not a single pathway operating uniformly across innovations or trust referents. Perceived risk and perceived benefits are therefore better represented as parallel appraisals than as sequential stages. Both were associated with consumer responses in the reviewed literature, with perceived risk generally showing less favourable and perceived benefits more favourable associations. However, their relative contribution varied across models. In Baum et al. [25], perceived benefits were more strongly related to behavioural intentions than perceived risk, while Harrison and Liem [27] found that the relative importance of benefit- and risk-related indirect relationships differed across trust domains. These findings indicate that benefits should not be treated simply as the absence of risk and do not support a universal compensatory process in which consumers consistently balance one against the other. Food-safety-related perceptions occupy a related but more specific position within the framework. They are treated as an appraisal domain closely related to perceived risk rather than as a separate explanatory construct. At the same time, the reviewed studies operationalised food safety in different ways, including general safety concerns, product-specific perceived safety, and safety-related food-choice motives. These measures should therefore not be treated as interchangeable. Some studies linked trust to food-safety-related perceptions, but these relationships were context- and referent-specific. For example, Muiruri [16] modelled food-safety concerns as a mediator between trust and willingness to try insect-based foods, Eom and Choy [108] experimentally examined how information about scientists’ motives shaped responses to scientific consensus on the safety of cultivated meat, and Mankad and Carter [124] qualitatively explored trust in regulatory oversight in relation to a precision-fermentation ingredient. Such findings justify representing trust and food-safety-related perceptions as related constructs, but do not establish a general causal ordering between them. FN and FTN were generally associated with less favourable responses to unfamiliar foods and production technologies, but they should not be treated as interchangeable. FN concerns reluctance towards unfamiliar foods, whereas FTN concerns reluctance towards foods produced using unfamiliar or novel technologies. Their relative relevance may therefore depend partly on whether unfamiliarity concerns the food itself, its production process, or both. The evidence also does not support a fixed sequence from neophobia to perceived risk and subsequent rejection. Only three included studies formally tested FN/FTN-related indirect pathways involving perceived risk and/or benefits and consumer responses [17,25,81]. Their findings were heterogeneous, indicating that associations between neophobia and perceived risk should not in themselves be interpreted as evidence that risk mediates consumer responses. This distinction is also relevant to studies in which FN or FTN were measured together with risk and benefit perceptions but formal indirect effects were not established. For example, Yaşa Özeltürkay et al. [60] reported associations of FTN with higher perceived risk and lower perceived benefits in the context of GM foods, but this should be distinguished from evidence of a formally tested indirect pathway to consumer responses. More generally, co-measurement, direct associations, and formal mediation tests represent different levels of evidence and should not be conflated. Disgust and perceived naturalness provide additional evaluative dimensions but should remain conceptually distinct from neophobia and perceived risk. Disgust represents an affective response, whereas perceived naturalness concerns evaluations of the product or production process. Studies of insect-based foods, cultivated meat, and other novel foods indicate that both have been associated with less favourable responses in particular contexts [92,93,94]. Experimental findings also indicate that naturalness-related responses may depend on framing and familiarity. Banovic and Grunert [104], for example, showed that responses to precision-fermentation products varied with the information provided about the production process. These findings support retaining disgust and naturalness as distinct constructs in the framework. However, because neither was used as an independent search domain, their treatment in this review should be regarded as complementary rather than exhaustive. A further implication concerns the distinction between consumer-response outcomes. The reviewed evidence was concentrated on attitudes, acceptance, willingness, and behavioural intentions, whereas studies assessing self-reported or observed behaviour were comparatively rare. Favourable attitudes or intentions, therefore, cannot be assumed to translate into actual purchasing, consumption, or sustained adoption. The framework consequently represents attitudes or acceptance, willingness or behavioural intentions, willingness to pay, choice or preference, and behavioural outcomes as distinct endpoints rather than as sequential stages of an adoption process. Figure 2 accordingly provides a conceptual organisation of the reviewed evidence. It distinguishes direct associations, risk- and benefit-related appraisal pathways, and co-measurement or associations for which direction or temporal ordering remains unresolved. Formal indirect effects were tested only for a subset of the appraisal pathways represented in the framework. Contextual characteristics such as the food or technology examined, population, familiarity, and information exposure are best interpreted as potential sources of heterogeneity rather than as established moderators of every relationship. Similarly, socioeconomic context should be treated as an interpretive lens rather than assumed to modify the psychological relationships represented in the framework.

The practical implications should likewise be interpreted cautiously. The review does not demonstrate that a single communication or assurance strategy will improve acceptance across food innovations. Rather, it suggests that communication may need to address the specific uncertainties relevant to a given product or technology, including who is responsible for oversight, how the product is produced, what evidence supports its safety, and which claimed benefits are substantiated. Transparency, credible information sources, assurance mechanisms, and opportunities for familiarisation are plausible approaches for further evaluation, rather than interventions shown by this review to work uniformly across settings.

### 4.2. Evidence from LICs/LMICs and Contextual Considerations

Socioeconomic context may shape consumer responses to sustainability-relevant food innovations and to their relationships with novelty-related dispositions, trust, perceived risk, perceived benefits, and food-safety-related perceptions. However, the evidence in this review does not establish systematic differences by country-income group. Twenty-one of the 288 included studies (7.3%) involved participants from at least one low-income or lower-middle-income country, according to the country-income classification used in this review; five of these studies included participants from LICs. Coverage was geographically concentrated: eight of these studies included participants from India, and eight from sub-Saharan Africa. Although several studies compared countries, the reviewed evidence does not establish country-income classification as a moderator of the relationships examined. Income classification is therefore used here as an interpretive context rather than as an explanation for differences in consumer responses. Nevertheless, studies conducted in LICs/LMICs illustrate that trust, perceived risk, familiarity, and novelty-related dispositions can influence consumer responses to sustainable food innovations. In Myanmar, trust in producers was positively associated with the intention to consume reared crickets [68], while in rural Kenya intentions to consume insect-based foods were higher among participants who were more familiar with the practice [106]. In Vietnam, blockchain-enabled reviews and traceability increased trust in technology and perceived transparency of environmental information, with transparency positively associated with sustainable purchase intention [134]. Studies in Uganda, Chad, Nigeria, the Democratic Republic of the Congo, Mali, and Niger likewise identified food neophobia, food-technology neophobia, perceived risk, disgust, familiarity, or culturally specific beliefs as correlates of consumer responses [43,126,133,135]. However, these findings do not indicate that any of these constructs is consistently more influential in LICs/LMICs than in higher-income settings.

Cross-national studies further show that differences in the levels of psychological constructs and consumer responses need not imply different directions of association. In Bryant et al. [127], acceptance of plant-based and cultured meat was higher in India and China than in the USA, while food neophobia was higher in India; nevertheless, greater familiarity and lower food neophobia predicted higher acceptance across all three countries. Giacalone and Jaeger [84] similarly identified substantial cross-country variation in responses to novel food technologies, while Tsimitri et al. [80] found different patterns of food and food-technology neophobia in Uganda, Greece, and Cyprus. Such findings demonstrate cross-context heterogeneity but do not isolate national income level as its source.

Interpretation is further constrained by the characteristics of the available evidence. Many LIC/LMIC studies relied on urban, young, relatively advantaged, or online samples, although some included rural populations, limiting generalisability to the wider populations of these countries. As in the overall evidence base, most studies assessed acceptance, intentions, or willingness rather than observed behaviour. Moreover, the meaning of novelty itself may be context dependent. For edible insects, which are culturally familiar foods in some settings, familiarity and local food practices may modify the relevance of food neophobia: in Uganda, familiarity and cultural context were more strongly related to future consumption than food neophobia [43], and greater familiarity was associated with stronger consumption intentions in rural Kenya [106].

Future comparative research should therefore distinguish between country-income classification and the specific contextual characteristics that may influence consumer evaluations. Equivalent measures and tests of measurement invariance across settings would be particularly important before differences in FN, FTN, trust, or risk perception levels and their associations are interpreted. The current evidence supports caution in transferring psychological models developed predominantly in high-income settings to LICs/LMICs, but does not justify assuming distinct psychological mechanisms on the basis of country-income classification alone. Sustainable food policies should be adapted to local socioeconomic contexts. Policies and interventions shown to be effective in high-income countries may not be directly transferable to LMIC settings. Similarly, scales developed in high-income countries to measure psychosocial factors associated with sustainable food choices may require adaptation and validation before use in LMICs. Addressing food safety concerns, strengthening institutional trust, and addressing risk perceptions in LMICs may be important for promoting sustainable food consumption. Acceptance in LMIC settings may depend on perceived safety, affordability, and local relevance, a proposition that requires direct comparative testing.

### 4.3. Research Gaps and Future Directions

Several studies have examined models involving trust, perceived risks and benefits, and consumer responses, but they differ substantially in the constructs included, trust referents, food innovations, and outcomes considered. Direct associations are better documented than formally tested indirect relationships, and the available evidence does not support a single explanatory pathway linking novelty-related dispositions, trust, risk and benefit perceptions, and consumer responses. Future research should therefore test competing or concurrent pathways within the same analytical framework, preferably using comparable measures across a limited set of innovations or trust referents. Such designs would help determine whether the observed associations are better represented by sequential mechanisms, interrelated evaluative processes, or a broader response to the innovation. Outcome coverage represents a further gap in the evidence base. Most studies assessed attitudes, acceptance, willingness, or behavioural intentions, while comparatively few examined self-reported behaviour, observed choice, or other behaviourally anchored outcomes. Where feasible, future studies should therefore complement stated responses with behavioural or incentive-compatible measures. Geographical coverage is similarly uneven. Only 21 of the 288 included studies involved participants from at least one low-income or lower-middle-income country according to the World Bank classification used in this review, indicating substantial underrepresentation of these settings. The available evidence is insufficient to determine whether the identified relationships vary systematically by country-income context. Comparative research should therefore use equivalent constructs and measurement approaches across settings and, where feasible, examine specific contextual characteristics that may plausibly affect these relationships. Additionally, future studies could explore whether sociodemographic factors, such as age, education, income, gender, urban–rural location, and cultural background, influence consumers’ psychological responses toward sustainable food choices. This may help to better understand differences among consumer groups and develop more targeted strategies for promoting sustainable food adoption.

### 4.4. Strengths and Limitations

A strength of this review is its integration of a broad and heterogeneous evidence base, with study-level information provided in the Supplementary Material. By distinguishing related constructs, types of evidence, and consumer-response outcomes, the synthesis offers a transparent conceptual organisation of the literature without imposing a single causal sequence. Several limitations and boundary conditions should also be considered when interpreting this review. First, no formal, design-specific assessment of methodological quality or risk of bias was undertaken because of the heterogeneity of the included quantitative and qualitative evidence. The synthesis therefore could not weight findings by study quality and relied on peer-reviewed status as a baseline inclusion criterion, which limits the confidence that can be assigned to individual relationships.

Second, retrieval was restricted to English-language, peer-reviewed empirical articles indexed in the Web of Science Core Collection and Scopus, and studies without accessible full texts were excluded. Relevant studies indexed elsewhere, published in other languages, or available as grey literature may therefore have been missed, and publication and selective-reporting biases could not be assessed.

Third, the included studies varied in design, product context, construct definition, and measurement, which precluded a meta-analysis. The review therefore provides no pooled effect estimates. Trust in different actors and perceptions of different risk domains are not necessarily equivalent, and grouping them within broader categories involved interpretive decisions. Fourth, the evidence is predominantly based on stated evaluations, willingness, and intentions, while self-reported and observed behaviour are less well represented. The findings therefore primarily describe evaluations of food innovations rather than confirmed adoption, and the review cannot estimate the magnitude or persistence of any attitude–behaviour gap. Fifth, cross-sectional associations, including indirect effects estimated from cross-sectional data, limit inferences about temporal ordering and causality. The integrated conceptual framework combines relationships identified across diverse studies, but its complete configuration has not been evaluated within a unified empirical design; it is intended to guide the formulation of hypotheses. Finally, the review focuses on selected constructs within sustainability-relevant food innovation contexts and does not comprehensively address structural determinants of food consumption, such as affordability, availability, or habit.

## 5. Conclusions

This review indicates that consumer responses to sustainability-relevant food innovations are not adequately represented by a single trust–risk–acceptance pathway. Instead, the reviewed evidence points to a set of context-dependent relationships among trust, perceived risks and benefits, food safety concerns, neophobia, perceived naturalness, and disgust. The role of trust varied across studies and trust referents: social trust, trust in authorities, producers, retailers, scientists, and other institutions were not consistently associated with consumer responses in the same way. This heterogeneity suggests that trust should be treated as a referent-specific construct rather than as a single interchangeable predictor.

The evidence also provides only qualified support for models in which trust or neophobia influences acceptance through perceived risk. Associations between these constructs and risk-related evaluations were commonly observed, but formal tests of indirect effects were less common and produced heterogeneous results. Where both perceived risks and benefits were examined, both could contribute to consumer responses, although perceived benefits showed stronger relationships or indirect effects in some models. The available evidence therefore does not establish either risk or benefit as a universal mediating pathway. Rather, their relative importance appears to depend on the innovation, the trust referent, and the way acceptance and its antecedents are operationalised.

The findings further show that perceived risk does not exhaust the psychological responses associated with unfamiliar food innovations. Food-safety-related perceptions were closely related to perceived risk but were operationalised as safety concerns, perceived product safety, or food-choice motives, and were sometimes modelled as mediators, limiting comparability across studies. Perceived risk also did not encompass all evaluations associated with unfamiliar food innovations: perceived naturalness and disgust emerged as further relevant evaluations, particularly for alternative proteins and technologically produced foods, although they were identified as complementary themes rather than reviewed exhaustively. Interpretation of these relationships is constrained by the structure of the evidence base. The reviewed studies predominantly assessed attitudes, acceptance, willingness to try or buy, and purchase intentions rather than observed purchasing or consumption. Cross-sectional survey designs were also common, limiting causal interpretation of associations and mediation models. Although experimental studies were present, product categories and geographical settings were unevenly represented, making broad generalisation across sustainable food innovations premature.

## Data Availability

No new data were created or analyzed in this study. All extracted data from the reviewed literature are available in the article and Supplementary Material.

## Supplementary Materials

The following supporting information can be downloaded at: https://www.mdpi.com/article/10.3390/nu18213509/s1. Table S1: Complete Database-Specific Search Strategies Used for Literature Identification in Web of Science and Scopus; Table S2: Characteristics and Key Findings of Included Studies (N = 288).
