# Supplementary material for: Neophobia, Trust, and Perceived Risk in Consumer Responses to Sustainability-Relevant Food Innovations: An Integrative Review and Conceptual Framework
Source: Nutrients. 2026 Oct 25;18(21):3509. doi: 10.3390/nu18213509 (PMC13764698; doi:10.3390/nu18213509)
Supplement: Supplementary file 1 [file nutrients-18-03509-s001.zip › Supplementary_Table S1.pdf]

Supplementary Material

Table S1. Complete Database-Specific Search Strategies Used for Literature Identification in Web of Science and Scopus

No.	Search Topic	Database	Search Field	Main Search Concept	Results
1	Food Neophobia	Web of Science	Topic Search (TS)	(("sustainable food*" OR "sustainable diet*" OR "sustainable food choice*" OR "sustainable food consumption" OR "alternative protein*" OR "protein transition" OR "food innovation*" OR "novel food*" OR "novel food technolog*" OR "cellular agriculture" OR "cultured meat" OR "cultivated meat" OR "cell-based meat" OR "clean meat" OR "in vitro meat" OR "lab-grown meat" OR "edible insect*" OR "insect-based food*" OR "entomophagy" OR "insect protein*" OR "plant-based meat" OR "meat analogue*" OR "meat substitute*" OR "plant-based dairy" OR "dairy alternative*" OR "mycoprotein*" OR "precision fermentation") AND ("food neophobi*"))	510 papers
2	Food Neophobia	Scopus	Title-Abstract-Keywords	(("sustainable food*" OR "sustainable diet*" OR "sustainable food choice*" OR "sustainable food consumption" OR "alternative protein*" OR "protein transition" OR "food innovation*" OR "novel food*" OR "novel food technolog*" OR "cellular agriculture" OR "cultured meat" OR "cultivated meat" OR "cell-based meat" OR "clean meat" OR "in vitro meat" OR "lab-grown meat" OR "edible insect*" OR "insect-based food*" OR "entomophagy" OR "insect protein*" OR "plant-based meat" OR "meat analogue*" OR "meat substitute*" OR "plant-based dairy" OR "dairy alternative*" OR "mycoprotein*" OR "precision fermentation") AND ("food neophobi*"))	509 papers
3	Food Technology Neophobia	Web of Science	Topic Search (TS)	(("sustainable food*" OR "sustainable diet*" OR "sustainable food choice*" OR "sustainable food consumption" OR "alternative protein*" OR "protein transition" OR "food innovation*" OR "novel food*" OR "novel food technolog*" OR "cellular agriculture" OR "cultured meat" OR "cultivated meat" OR "cell-based meat" OR "clean meat" OR "in vitro meat" OR "lab-grown meat" OR "edible insect*" OR "insect-based food*" OR "entomophagy" OR "insect protein*" OR "plant-based meat" OR "meat analogue*" OR "meat substitute*" OR "plant-based dairy" OR "dairy alternative*" OR "mycoprotein*" OR "precision fermentation") AND ("food technology neophobi*" OR "technology neophobi*" OR "Food Technology Neophobia Scale"))	80 papers
4	Food Technology Neophobia	Scopus	Title-Abstract-Keywords	(("sustainable food*" OR "sustainable diet*" OR "sustainable food choice*" OR "sustainable food consumption" OR "alternative protein*" OR "protein transition" OR "food innovation*" OR "novel food*" OR "novel food technolog*" OR "cellular agriculture" OR "cultured meat" OR "cultivated meat" OR "cell-based meat" OR "clean meat" OR "in vitro meat" OR "lab-grown meat" OR "edible insect*" OR "insect-based food*" OR "entomophagy" OR "insect protein*" OR "plant-based meat" OR "meat analogue*" OR "meat substitute*" OR "plant-based dairy" OR "dairy alternative*" OR "mycoprotein*" OR "precision fermentation") AND ("food technology neophobi*" OR "technology neophobi*" OR "Food Technology Neophobia Scale"))	69 papers
5	Trust	Web of Science	Topic Search (TS)	(("sustainable food*" OR "sustainable diet*" OR "sustainable food choice*" OR "sustainable food consumption" OR "alternative protein*" OR "protein transition" OR "food innovation*" OR "novel food*" OR "novel food technolog*" OR "cellular agriculture" OR "cultured meat" OR "cultivated meat" OR "cell-based meat" OR "clean meat" OR "in vitro meat" OR "lab-grown meat" OR "edible insect*" OR "insect-based food*" OR "entomophagy" OR "insect protein*" OR "plant-based meat" OR "meat analogue*" OR "meat substitute*" OR "plant-based dairy" OR "dairy alternative*" OR "mycoprotein*" OR "precision fermentation") AND (trust* OR "consumer confidence" OR "institutional confidence"))	505 papers
6	Trust	Scopus	Title-Abstract-Keywords	(("sustainable food*" OR "sustainable diet*" OR "sustainable food choice*" OR "sustainable food consumption" OR "alternative protein*" OR "protein transition" OR "food innovation*" OR "novel food*" OR "novel food technolog*" OR "cellular agriculture" OR "cultured meat" OR "cultivated meat" OR "cell-based meat" OR "clean meat" OR "in vitro meat" OR "lab-grown meat" OR "edible insect*" OR "insect-based food*" OR "entomophagy" OR "insect protein*" OR "plant-based meat" OR "meat analogue*" OR "meat substitute*" OR "plant-based dairy" OR "dairy alternative*" OR "mycoprotein*" OR "precision fermentation") AND (trust* OR "consumer	578 documents

				confidence" OR "institutional confidence"))	
7	Risk Perception	Web of Science	Topic Search (TS)	(("sustainable food*" OR "sustainable diet*" OR "sustainable food choice*" OR "sustainable food consumption" OR "alternative protein*" OR "protein transition" OR "food innovation*" OR "novel food*" OR "novel food technolog*" OR "cellular agriculture" OR "cultured meat" OR "cultivated meat" OR "cell-based meat" OR "clean meat" OR "in vitro meat" OR "lab-grown meat" OR "edible insect*" OR "insect-based food*" OR "entomophagy" OR "insect protein*" OR "plant-based meat" OR "meat analogue*" OR "meat substitute*" OR "plant-based dairy" OR "dairy alternative*" OR "mycoprotein*" OR "precision fermentation") AND ("risk perception*" OR "perceived risk*" OR "risk concern*" OR uncertaint*))	601 results
8	Risk Perception	Scopus	Title-Abstract-Keywords	(("sustainable food*" OR "sustainable diet*" OR "sustainable food choice*" OR "sustainable food consumption" OR "alternative protein*" OR "protein transition" OR "food innovation*" OR "novel food*" OR "novel food technolog*" OR "cellular agriculture" OR "cultured meat" OR "cultivated meat" OR "cell-based meat" OR "clean meat" OR "in vitro meat" OR "lab-grown meat" OR "edible insect*" OR "insect-based food*" OR "entomophagy" OR "insect protein*" OR "plant-based meat" OR "meat analogue*" OR "meat substitute*" OR "plant-based dairy" OR "dairy alternative*" OR "mycoprotein*" OR "precision fermentation") AND ("risk perception*" OR "perceived risk*" OR "risk concern*" OR uncertaint*))	708 results
9	Food Safety Perception	Web of Science	Topic Search (TS)	(("sustainable food*" OR "sustainable diet*" OR "sustainable food choice*" OR "sustainable food consumption" OR "alternative protein*" OR "protein transition" OR "food innovation*" OR "novel food*" OR "novel food technolog*" OR "cellular agriculture" OR "cultured meat" OR "cultivated meat" OR "cell-based meat" OR "clean meat" OR "in vitro meat" OR "lab-grown meat" OR "edible insect*" OR "insect-based food*" OR "entomophagy" OR "insect protein*" OR "plant-based meat" OR "meat analogue*" OR "meat substitute*" OR "plant-based dairy" OR "dairy alternative*" OR "mycoprotein*" OR "precision fermentation") AND ("food safety perception*" OR "food safety concern*" OR "perceived food safety" OR "perceived safety" OR "safety concern*"))	376 records
10	Food Safety Perception	Scopus	Title-Abstract-Keywords	(("sustainable food*" OR "sustainable diet*" OR "sustainable food choice*" OR "sustainable food consumption" OR "alternative protein*" OR "protein transition" OR "food innovation*" OR "novel food*" OR "novel food technolog*" OR "cellular agriculture" OR "cultured meat" OR "cultivated meat" OR "cell-based meat" OR "clean meat" OR "in vitro meat" OR "lab-grown meat" OR "edible insect*" OR "insect-based food*" OR "entomophagy" OR "insect protein*" OR "plant-based meat" OR "meat analogue*" OR "meat substitute*" OR "plant-based dairy" OR "dairy alternative*" OR "mycoprotein*" OR "precision fermentation") AND ("food safety perception*" OR "food safety concern*" OR "perceived food safety" OR "perceived safety" OR "safety concern*"))	421 records

Note on Search Strategy: The date of the last search conducted across all databases was September 2, 2026.
