# Supplementary material for: Neophobia, Trust, and Perceived Risk in Consumer Responses to Sustainability-Relevant Food Innovations: An Integrative Review and Conceptual Framework
Source: Nutrients. 2026 Oct 25;18(21):3509. doi: 10.3390/nu18213509 (PMC13764698; doi:10.3390/nu18213509)
Supplement: Supplementary file 1 [file nutrients-18-03509-s001.zip › Supplementary_Table S2.pdf]

Supplementary Material

Table S2. Characteristics and Key Findings of Included Studies (N=288)

Sl.	Study (Author/Year)	Population & Setting	Study Design & Measurement	Food / Product Context	Psychological Construct(s) & Specifics	Consumer Response Outcome	Sustainability Relevance & Rationale	Main Findings & Framework Relevance
1	Roccatello et al. (2024)	N=303, Italy, Adults	Online survey; Food Neophobia Scale (FNS)	Fish fillets obtained from fish fed an insect-based feed	Food neophobia; Perceived sustainability of feed; Attitudes on climate change	Acceptance; Willingness to Buy	Yes - Explores insect-based feed as a sustainable alternative to mitigate the environmental impact of conventional aquaculture feed.	Higher food neophobia negatively influenced perceived feed quality and acted as a barrier to acceptance and willingness to buy.
2	Onwezen et al. (2022)	N=4961 (across 3 samples), Netherlands, Adults	Online survey (cross-sectional & longitudinal); FNS, Domain-specific innovativeness	Fish, seaweed, legumes, insects, cultured meat	Food neophobia; Domain-specific innovativeness; Positive/negative emotions	Intention; Self-reported consumption (Actual behavior)	Yes - Explicitly framed around the "protein transition" towards more plant-based/meat-less diets for environmental sustainability.	Positive emotions strongly predicted intentions over time; food neophobia predicted self-reported consumption for some proteins cross-sectionally.
3	Menna et al. (2026)	N=470, Italy, Adults	Online questionnaire (info treatments); FNS	Pasta enriched with cricket, spirulina, hemp, or pea flour	Food neophobia; General health interest; Food consumption sustainability	Willingness to Try; Willingness to Buy	Yes - Investigates alternative protein sources to reduce the environmental footprint (land/water/emissions) of conventional proteins.	Low food neophobia was consistently associated with greater willingness to try and buy; highly neophobic consumers were resistant even when given positive sustainability information.
4	Sato & Ishizuka (2022)	N=683, Japan (Kanto & Nagano)	Web-based survey; FNS, Food Technology Neophobia Scale (FTNS)	Traditional insects (grasshoppers, wasps) and Novel insects (cricket cookies, fried crickets)	Food neophobia; Food technology neophobia; Anxiety concerning hygiene and taste	Willingness to Eat (Intention); Willingness to Substitute (meat/seafood)	Yes - Assesses substituting meat/seafood with insects, framed by the FAO report on environmental conservation.	FNS and FTNS negatively impacted the willingness to eat novel insects; tradition weakened this neophobic effect, but taste anxiety heavily hindered acceptance.
5	La Barbera et al. (2017)	N=118, Italy, University students	Lab experiment & follow-up; FNS, Implicit Association Test (IAT)	Chocolate bar enriched with cricket proteins	Food neophobia; Disgust; Implicit attitudes toward insects	Intention (to eat/buy)	Yes - Highlights insects' low environmental impact (lower greenhouse gas emissions and feed conversion rates) compared to conventional livestock.	Food neophobia and disgust independently reduced the intention to eat insect-based food; disgust was a considerably stronger predictor than neophobia.
6	Chia et al. (2024)	N=1224, Singapore, Adults	Online survey; Modified Food Neophobia Scale	Plant-based meat alternatives, cultured meat, insect-based products	Food neophobia; Un-naturalness; Drug residue concerns; Zoonotic diseases	Consumption intent; WTP	Yes - Explores the transition toward sustainable alternative protein sources to address environmental and health challenges.	Un-naturalness was the strongest barrier; food neophobia negatively impacted consumption intent specifically for insect-based products.
7	Inguscio et al. (2026)	N=36, Italy, Adults	Lab experiment (EEG & VAS); FNS, LOHAS	Plant-based vs. animal-based meat, fish, and dairy	Food neophobia; LOHAS; Implicit Approach-Withdrawal (AW); Mental Workload (WL)	Attitude/Liking; Purchase intention	Yes - Fostering sustainable dietary transitions to lower the food sector's environmental footprint.	Food neophobia correlated with increased mental workload and reduced approach motivation across both conventional and plant-based foods.
8	Spendrup & Hovmalm (2022)	N=483, Sweden, Meat eaters to flexitarians	Online survey; FNS	Vegetarian nuggets, soy mince, pre-cooked beans	Food neophobia; Health concern; Climate change conviction	Self-reported consumption (Actual behavior)	Yes - Replacing meat with plant-based protein as a step in an urgent transition towards a sustainable diet.	Food neophobia had a significant negative effect on the actual consumption of both PBMA and pulses among omnivores.
9	Süren & Çetin (2025)	N=402, Turkey, Chef candidates (University students/graduates)	Online survey; Structural Equation Modeling	Artificial meat (cultured/in vitro/cloned)	Environmental concern; Food safety concern; Attitudes toward artificial meat	Purchasing Intention	Yes - Adopting artificial meat to solve complex environmental and greenhouse gas emission problems associated with traditional meat.	Environmental concern positively affected artificial meat attitudes, which in turn increased purchasing intention.
10	Hajhamidiasl et al. (2025)	N=1194, Turkey, Adults	Online survey; Structural Equation Modeling; Food Neophobia Scale (FNS)	Insect-based foods (IBFs)	Food neophobia; Social norms; Biospheric values; Attitude toward sustainability	Intention to eat; Number of chosen insect-based foods (visual choice)	Yes - Examines insect-based foods as a sustainable alternative protein source to reduce greenhouse gas emissions and resource use compared to traditional meat.	Food neophobia reduced the number of chosen insect-based foods; social norms positively influenced choice and reduced food neophobia. Sustainability attitudes strongly predicted intentions, but intentions did not always translate into actual visual choices.
11	Bahraseman et al. (2025)	N=445, Mashhad, Iran, Adults	In-person survey; Structural Equation Modeling (Extended TPB); Food Neophobia Scale	Algae or algae-based foods	Food neophobia; Food disgust; Environmental concerns; Moral norms; Subjective norms; Attitude	Intention to consume	Yes - Investigates algae as a sustainable, nutrient-rich alternative protein source to address environmental impacts and food security.	Food disgust significantly decreased attitude and intention to consume algae-based foods, while environmental concerns and moral norms had positive effects mediated by attitude. Food neophobia did not significantly impact consumption intentions.
12	Embling et al. (2022)	N=476, UK, Adults	Online survey; Structural Equation Modeling; FNS, Food Technology Neophobia Scale (FTNS)	Edible macroalgae (seaweeds) and seaweed-based products (burger, sushi, etc.)	Food neophobia; Food technology neophobia; Expected product attributes (Taste/edibility, familiarity)	Acceptance (Liking, Willingness to try, Willingness to buy, Readiness to adopt)	Yes - Explores seaweed as a sustainable, plant-based alternative to animal-based proteins to reduce environmental impact.	Food neophobia significantly decreased acceptance, but perceiving products as tasty/edible and familiar partially mediated and mitigated this negative effect.
13	de Koning et al. (2020)	N=3091, 9 countries (Global), Adults	Survey; Structural Equation Modeling; FNS, FTNS	Plant-based and insect-based meat alternatives	Food neophobia; Food technology neophobia; Environmental influence on food choice; Meat nutritional importance	Willingness to try, buy, and pay more (Intention)	Yes - Examines plant- and insect-based proteins as alternatives to traditional animal proteins due to environmental and health concerns.	Food neophobia inhibited behavioral intentions for both plant- and insect-based alternatives. Environmental impact importance positively influenced perceived suitability/benefits.
14	Karakuş et al. (2025)	N=444, Turkey, Adults	Online survey; Structural Equation Modeling; FNS	Artificial meat and edible insects	Food neophobia; Climate change risk perception	Diffusion optimism / Acceptance (Attitude)	Yes - Explicitly investigates alternative protein sources as a strategy to mitigate the impacts of	Climate change risk perception positively affected optimism/acceptance of artificial meat and edible insects, while food neophobia did

							climate change and manage food shortages.	not have a significant inhibiting or mediating effect in this context.
15	Kamenidou et al. (2023)	N=742, Greece, Generation Z Adults (18-27 years)	Online survey; Cluster analysis; FNS	Edible insects and insect-based foods/drinks	Food neophobia; Disgust; Perceived health risks; Extrinsic/intrinsic barriers	Willingness to try; Intention to consume	Yes - Explores entomophagy to address the environmental footprint of conventional meat and secure a sustainable future for the planet.	Disgust and perceived extrinsic/intrinsic cues were the main barriers to entomophagy. The Generation Z cohort was generally not food neophobic, meaning rejection was tied to disgust rather than novelty aversion.
16	Bazoche & Poret (2021)	N=301, France, Adults	Online survey (with info treatments); Logistic regression; FNS	Farmed trout fed with insect-based feed (aquafeed)	Food neophobia	Readiness to eat (Intention/Acceptance)	Yes - Examines insect meal in aquafeed as a sustainable alternative to fishmeal to preserve wild fishery resources and reduce overfishing.	Higher food neophobia decreased the probability of accepting insect-fed fish; however, providing information about the environmental benefits significantly increased acceptance across the sample.
17	Gumussoy et al. (2021)	N=158 (Study 1: 80, Study 2: 78), UK, University students/public	Lab experiment; Food Neophobia Scale (FNS)	Falafels (supposedly containing mealworm flour)	Food neophobia; Disgust; Tactile sensitivity; Familiarity	Food intake (Actual behavior); Desire to eat; Liking	Yes - Explicitly tests a "sustainability passage" and investigates entomophagy as a sustainable alternative animal protein to mitigate climate change.	A sustainability information passage reduced disgust (Study 1); increasing perceived familiarity of production also ameliorated disgust and maintained actual food intake (Study 2).
18	Tiboldo et al. (2024)	N=780, Italy, Adults	Online survey; Logistic regression; FNS	Poultry meat and eggs from insect-fed animals	Food neophobia; Entomophobia; Trust in public authorities; Sustainability belief	Willingness to buy (Intention)	Yes - Framed around a circular economy approach using insect feed to reduce natural resource exploitation and GHG emissions from conventional livestock.	Food neophobia and entomophobia negatively affected the likelihood of buying insect-fed poultry/eggs, while trust in public authorities and sustainable attitudes increased it.
19	Jilwana et al. (2025)	N=285, South India (Coimbatore), Adults	Direct survey; PLS-SEM; FNS	Plant-based meat alternatives (PBMA)	Food neophobia; Perceived meat importance; Animal welfare concern; Environmental concern; Attitude	Adoption intention	Yes - Examines PBMA adoption driven by environmental sustainability, citing that PBMA generates 90% less GHG and uses less land/water than traditional meat.	Food neophobia and perceived meat importance negatively influenced attitudes toward PBMA, while animal welfare concerns positively drove attitudes and subsequent adoption intention.
20	Boereboom et al. (2022)	N=1291, NL, UK, FR, ES, Adults	Online survey; Cluster analysis; FNS, FTNS	Cultured meat	Food neophobia; Food technology neophobia; Importance of environmental impact	Willingness to engage (try, purchase, pay more)	Yes - Investigates cultured meat as a sustainable alternative to traditional livestock to mitigate climate change and reduce land use.	Higher levels of food neophobia and food technology neophobia significantly resulted in a lower willingness to engage with cultured meat across the four countries.
21	Tan et al. (2016)	N=976, Netherlands, Adults	Online survey; Hierarchical linear regressions; FNS	Mealworms incorporated into familiar carriers (beef stew, curry, brownie, spice cake)	Food neophobia; Taste familiarity; Appropriateness	Willingness to try; Willingness to buy; Expected sensory-liking	Yes - Insects are investigated as a sustainable alternative to reduce the environmental impact of meat production.	Food neophobia negatively influenced acceptability; neophilic individuals and those with prior taste experience were significantly more willing to try insect-based foods.
22	Brunner & Nuttavuthisit (2020)	N=1042 (Switzerland and Thailand), Adults	Survey & Hierarchical cluster analysis; Food neophobia scale	Edible insects	Food neophobia; Relevance of sustainability, health, price, and taste	Willingness to eat / Regular adoption (Acceptance)	Yes - Entomophagy is framed around FAO arguments highlighting lower greenhouse gas emissions, feed, and space requirements compared to conventional meat farming.	Food neophobia strongly differentiated consumer segments, with "laggards" showing the highest food neophobia and rejecting insect consumption, while "early adopters" exhibited the lowest neophobia.
23	Olum et al. (2021)	N=310, Uganda, Adults	Structured questionnaire & Logistic regression; Food Neophobia Scale (FNS)	Edible insects (grasshoppers, termites)	Food neophobia; Familiarity; Socio-cultural factors	Current consumption (Actual behavior); Future consumption (Willingness to consume)	Yes - Edible insects are assessed as a sustainable meat alternative to mitigate the greenhouse gas emissions and heavy land/water use caused by livestock production.	High food neophobia significantly reduced the likelihood of currently consuming unfamiliar edible insects, though familiarity and cultural setting were stronger predictors for future consumption intentions.
24	Pittman et al. (2026)	N=1309, Philippines, Japan, China, Adults	Cross-national online survey & Ordinal logistic regression; Modified FNS	Gene-edited non-browning bananas	Food neophobia; Perceived safety; Cultural cognition (Individualism/Hierarchy)	Willingness to consume (WTC); Labeling preferences	Yes - The gene-edited banana is explicitly examined as an innovation to reduce post-harvest food waste and cut supply chain CO2 emissions by over 25%.	Food neophobia consistently and significantly reduced willingness to consume gene-edited bananas across all three countries, while perceived safety strongly increased it.
25	Hwang et al. (2020)	N=1017, Korea, Adults	Online survey & PLS regression; Food Neophobia Scale	Cultured meat and plant-based meat alternatives	Food neophobia; Sustainability; Unnaturalness; Distrust of biotechnology; Food curiosity	Willingness to buy (WTB)	Yes - Alternative meats are investigated to address the high energy, land, and water usage, as well as greenhouse gas emissions, associated with conventional livestock.	Food neophobia and distrust of biotechnology negatively affected the willingness to buy cultured meat, whereas food neophobia had no significant effect on plant-based meat alternatives.
26	Rabadán & Bernabéu (2021)	N=400, Spain (Madrid area), Adult wine consumers	Personal interviews (survey); Wine Neophobia Scale (WNS), Abbreviated Food Technology Neophobia Scale (AFTNS)	Wine (eco-innovative and organic)	Wine neophobia; Food technology neophobia; Environmental prioritization	Importance of attributes in purchasing decisions (Attitude)	Yes - Focuses on eco-innovation in wine production to reduce environmental impact and meet consumer demand for sustainable products.	Lower food technology neophobia and wine neophobia correlated with higher environmental awareness; however, the organic label remained the least valued purchasing attribute across all consumer segments.
27	Hielkema & Lund (2021)	N=1005, Denmark, Adults	Online survey; Multinomial & Binary logistic regression; FNS	Meat reduction (vegetarian/vegan meals vs. conventional meat)	Food neophobia; Identity-congruence; Habitual behavior; Social network influence; Climate awareness	Self-reported consumption (Actual behavior); Intention to reduce meat	Yes - Examines willingness to reduce meat consumption to address public health issues and mitigate human-induced greenhouse gas emissions and land use.	Food neophobia acted as a significant barrier, decreasing the likelihood of consumers progressing to the meat reducer stage, while awareness of the climate impact of meat strongly drove intention to reduce.
28	Platta et al. (2025)	N=947, Poland, University students	Online survey; Exploratory factor analysis (EFA) & K-	Insect-based foods	Food neophobia; Concern for food safety; Concern for	Willingness to consume	Yes - Explores entomophagy as a sustainable source of protein to	Food neophobia was a highly significant psychological barrier that reduced acceptance

		(Generation Z)	means clustering; FNS		environmental sustainability		address greenhouse gas emissions, natural resource depletion, and climate change.	of insect-based foods, whereas concern for environmental sustainability and food safety drove willingness to consume.
29	Moruzzo et al. (2021)	N=420, Italy (Pisa), Adults	Self-reported questionnaire; Logit model; FNS	Food containing insects	Food neophobia; Insect phobia (disgust, fear, social acceptance)	Probability of eating; Intention to eat	Yes - Investigates insect-based foods as an environmentally sound product and a sustainable protein alternative to address population growth and food security.	Both food neophobia and insect phobia significantly and negatively reduced the probability and intention of eating foods containing insects, highlighting them as major barriers to acceptance.
30	Hanan et al. (2026)	N=9, Malaysia (Klang Valley), Consumers	In-depth qualitative interviews; Transcendental phenomenological approach	Cultured meat (CM)	Food neophobia; Curiosity; Trust/Scepticism toward corporations; Unfamiliarity; Ethical/Religious concerns	Intention to consume / embrace	Yes - Cultured meat is explored as a sustainable solution to mitigate greenhouse gas emissions, deforestation, zoonotic diseases, and antimicrobial resistance linked to industrial animal farming.	Food neophobia, unfamiliarity, and mistrust of large corporations acted as major personal and societal barriers to CM acceptance, despite curiosity and acknowledgement of its environmental and safety benefits.
31	Castro-Alija et al. (2024)	N=459, Spain (Castilla y León), Adults across age groups	Cross-sectional survey; Food neophobia questions	Insect consumption (Entomophagy)	Food neophobia; Motivations (environmental, nutritional); Curiosity	Willingness to consume (Acceptance)	Yes - Entomophagy is framed as a sustainable alternative protein that drastically lowers greenhouse gas emissions and feed conversion ratios compared to conventional livestock.	Food neophobia and resistance were prominent barriers, particularly among older individuals; conversely, younger participants were more open to entomophagy, often motivated by environmental sustainability and curiosity.
32	Michel & Begho (2023)	N=248, UK, Adults	Online survey & discrete choice experiment; Food Neophobia Scale (FNS), Eco-consciousness scale	Insect-based foods (cricket powder sausages)	Food neophobia; Eco-consciousness; Perceived agency over environmental problems	Willingness to pay (WTP)	Yes - IBFs are analyzed as a low-emission alternative to meat that utilizes organic waste streams and requires less land and water.	High food neophobia significantly lowered the valuation and WTP for insect-based sausages, while high eco-consciousness and perceived agency correlated with higher valuations and increased receptiveness to environmental information.
33	Pickering et al. (2025)	N=1276 (UK) & 1532 (Japan), Adults	National survey data & OLS regression	Seaweed	Food neophobia; Risk-taking propensity; Institutional trust; Political orientation	Current consumption (Actual behavior); Likelihood to eat (Intention)	Yes - Seaweed is assessed as a sustainable food source that sequesters carbon, mitigates climate change, and grows without synthetic fertilizers, freshwater, or arable land.	Risk-taking propensity consistently increased current and future seaweed consumption, overcoming unfamiliarity/food neophobia barriers in the UK; additionally, left-wing political orientation drove UK acceptance whereas right-wing orientation drove Japanese consumption.
34	Gençer Bingöl & Ağagündüz (2025)	N=504, Türkiye, Young Adults (18-40 years)	Online cross-sectional survey; Structural Equation Modeling; Food Neophobia Scale (FNS)	Cultured meat	Food neophobia; Sustainable nutrition awareness; Animal welfare attitudes; Expectations and concerns	Acceptance / Intention to consume	Yes - Cultured meat is evaluated as an alternative protein source to address the environmental burdens (greenhouse gas emissions, water, energy, land use) of traditional meat consumption.	Food neophobia indirectly hindered acceptance by lowering expectations, while sensitivity to sustainable nutrition and animal welfare increased concerns but did not directly dictate the acceptance of cultured meat.
35	Dontsop Nguezet et al. (2024)	N=1560, DR Congo, Mali, Niger, Adults	Face-to-face survey; Generalized ordered logit (GOLOGIT) model	Food products (eggs, meat, fish) from animals fed on Black-soldier fly larvae (BSFL)	Cultural beliefs; Perceived animal health risks; Socioeconomic factors; Group membership	Degree of agreement to buy (Intention)	Yes - BSFL as animal feed is investigated as an eco-friendly and cost-efficient alternative to conventional feed in order to reduce the environmental footprint and greenhouse gas emissions of meat production.	Positive cultural beliefs and a lack of perceived animal health risks reduced the likelihood of disagreeing to buy BSFL-derived food, while group membership and education increased acceptance.
36	Alabdulmohsen & Al-Otaibi (2025)	N=2208, Saudi Arabia, Adults	Online cross-sectional survey; Multiple regression; Food Neophobia Scale (FNS)	Edible insects	Food neophobia; Disgust; Environmental concerns; Health benefits; Religious belief (Halal)	Willingness to consume / Acceptance	Yes - Edible insects are examined as a sustainable and eco-friendly alternative protein source to address global food security, climate change, and resource constraints.	Food neophobia, disgust, and sociocultural fears were significant barriers to willingness, while younger age, being male, and environmental/health concerns were strong positive predictors.
37	Tiboldo et al. (2024)	N=850+, Italy, Consumers	Online survey; T-test & Ordered logit regression	Insect-fed poultry products (meat and eggs)	Food neophobia (implicit); Trust in food safety system; Environmental awareness; Beliefs about animal welfare	Willingness to buy; Willingness to pay (Price premium)	Yes - Investigates insect-fed poultry to improve the sustainability of livestock production and promote a more circular supply chain with environmental benefits.	Consumers willing to buy insect-fed poultry showed higher environmental awareness and trust in food safety, with their willingness to pay a premium heavily driven by positive beliefs about animal welfare and expected quality.
38	Jung & Sharma (2025)	N=341 (Study 1) & N=119 (Study 2), USA, Adults/Students	Online experiment (Study 1) & Lab experiment (Study 2); UTAUT model; CB-SEM & Mediation analysis; FNS	Cultured meat (and plant-based meatball deception in lab)	Food neophobia; Hedonic motivation; Cost/Benefit perception; Social influence; Trust in regulatory system/industry	Behavioral intention; Actual behavior (Amount consumed)	Yes - Cultured meat is examined as a sustainable innovation to drastically reduce the greenhouse gas emissions, land use, and water consumption caused by traditional livestock agriculture.	Hedonic motivation was the strongest predictor of behavioral intention, while food neophobia significantly and negatively impacted actual consumption behavior in the lab setting.
39	Yılmaz & Güldüren (2026)	N=475, Turkey, Young adults (18–30 years)	Questionnaire survey; Food Neophobia Scale (FNS); Sustainable and Healthy Eating Behaviours Scale (SHEBS)	Alternative protein sources (plant-based products, mushroom protein, algae, artificial meat, edible insects)	Food neophobia; Sustainable and healthy eating behaviors (local food, seasonal food, meat reduction); Gender	Acceptance of alternative protein sources	Yes - Positioned to mitigate environmental impacts of livestock, including greenhouse gas emissions, carbon/water footprints, and natural resource depletion.	Plant-based proteins had the highest acceptance as alternatives to meat, while edible insects were rejected by 100% of participants; food neophobia was significantly negatively correlated with the behavior of reducing meat consumption.

40	Welk et al. (2025)	N=1114, Germany, Adults	Quantitative online survey & Linear regression; PANAS; Food Neophobia Scale (FNS); Food Choice Questionnaire (FCQ)	Innovative broccoli leaf products (kale alternative, soup, smoothie, pesto, wrap)	Mood (positive/negative); Food neophobia; Purchase motives (familiarity, certification marks, gentle processing, regional cultivation, price); Lifestyle	Willingness to buy	Yes - Examines utilization of discarded broccoli cultivation side streams (leaves) to reduce food waste, enhance resource efficiency, and lower nitrogen emissions.	Positive mood, low food neophobia, and a sustainability-oriented lifestyle significantly increased willingness to buy broccoli leaf products, whereas high food neophobia and preference for familiar products significantly hindered it.
41	Riverso et al. (2023)	N=148, Italy, Young adults / students (18–31 years)	Computer-based laboratory experiment (2x2 between-subjects ANOVA); Video manipulation; Food Neophobia Scale (FNS)	Insect-based foods (cricket flour tortilla vs. baked raw crickets; Acheta domesticus)	Message Sensation Value (MSV: high vs. low); Food neophobia (FNS); Emotional arousal	Intention to eat raw/visible insects; Intention to eat processed/non-visible insects	Yes - Framed around edible insects as sustainable alternative protein sources offering high feed-conversion efficiency, lower greenhouse gas/ammonia emissions, and reduced land/water use.	High food neophobia strongly decreased consumption intention across products; for low-neophobia consumers, high MSV messages increased intention to eat visible insects, whereas for high-neophobia consumers, high MSV messages backfired and reduced intention to eat processed insect foods.
42	Amaral et al. (2025)	N=510, Brazil, Adults (≥18 years)	Online survey; Descriptive and frequency analysis; 5-point Likert scales	Edible insects as alternative protein (snacks, processed foods, whole vs. pulverized)	Knowledge/familiarity of entomophagy; Eating habits; Disgust/repulsion; Environmental perspective; Ethical perspective; Food neophobia/novelty	Intention to consume / Willingness to buy	Yes - Evaluates insect protein as an eco-friendly alternative to counter animal welfare concerns, global warming, water scarcity, and intensive resource use of conventional meat.	Eating habits and disgust were primary negative barriers, but environmental perspective was the top positive motivator; 52.9% showed willingness to buy insect products, strongly preferring invisible/pulverized insect ingredients in familiar formats like snacks.
43	Mumbi et al. (2025)	N=990, UK, Adults (640 regular meat consumers vs. 350 meat avoiders-reducers)	Cross-sectional online survey; Partial Least Squares Structural Equation Modelling (PLS-SEM) & Multigroup Analysis (MICOM); Theory of Planned Behaviour	Grass-derived proteins (ryegrass, moor grass)	Facilitators (health, safety, nutrition); Positive/negative attitudes toward grass protein; Positive attitudes toward meat; Food neophobia; Subjective norms; Perceived behavioural control	Intention to try/consume	Yes - Explores grass-derived protein as a sustainable alternative utilizing abundant grasslands to alleviate environmental strain, land/water usage, and resource depletion from livestock.	Facilitators (health, safety, nutrition) and positive attitudes toward grass protein were the primary drivers of intention; direct facilitator pathways dominated for meat avoiders-reducers, while regular meat consumers showed higher resistance linked to cultural meat attachment.
44	Pagliarini et al. (2022)	N=302, Italy, Adults	Laboratory test (tasting); ANOVA & PCA; Health and Taste Attitude Scale (HTAS), Dutch Eating Behavior Questionnaire (DEBQ), Food Neophobia Scale (FNS)	Beetroot purees added with phenol extract from unripe grapes (winemaking by-products)	Food neophobia; Eating behavioral attitudes (emotional eating, restrained eating); Taste/Hedonic ratings (sour, sweet, astringent)	Liking / Acceptance (Actual sensory evaluation)	Yes - Phenol extracts from unripe grapes (winemaking by-products) are investigated as sustainable ingredients to promote the reuse of agricultural waste within the food chain.	Acceptance of phenol-enriched purees was polarized; a segment of consumers preferred high-phenol samples characterized by sourness/astringency, and these "high-likers" had significantly lower food neophobia and higher emotional eating scores.
45	Wendt & Weinrich (2025)	N=1099, Germany, Adults	Online survey; Latent profile analysis (LPA); Consumer trust toolkit, Abbreviated Food Technology Neophobia Scale (AFTNS)	Cultured meat	Trust (in EFSA, industry, product, interpersonal); Food technology neophobia; Sociodemographics	Acceptance / Purchase intention	Yes - Cultured meat is explored as a sustainable technology offering lower greenhouse gas emissions, reduced land/water demand, and improved animal welfare compared to industrial animal husbandry.	Latent profile analysis revealed four trust segments; the "Trusting Consumers" (17.5%) had the highest acceptance and lowest food technology neophobia, while "Distrustful Consumers" (24.3%) rejected cultured meat due to high neophobia and deep distrust of food safety institutions and manufacturers.
46	Wilkinson et al. (2018)	N=820, Australia, Adults	Online survey; ANOVA; Food Neophobia Scale (FNS)	Edible insects (witchetty grubs, ants, crickets, etc.) and insect-based products	Food neophobia; Demographics (sex, age, ethnicity); Prior consumption; Taste/appearance perception	Willingness to try	Yes - Entomophagy is advocated to meet global food demand while producing substantially less greenhouse gas emissions and requiring less water, feed, and space than conventional meat.	Food neophobia was a major barrier, with neophobic consumers largely unwilling to try any insects; however, incorporating insects into familiar formats (e.g., biscuits) or having prior consumption experience significantly improved willingness to try.
47	Diaz Vicuna et al. (2026)	N=900, Italy, Beef consumers	Online survey (CAWI); Principal Component Analysis (PCA) & K-means cluster analysis	Beef from cattle fed hazelnut skins (agro-industrial by-products / upcycled animal feed)	Food neophobia/neophilia, green self-identity, food waste reduction awareness/concern, animal feeding awareness/concern	Acceptability, environmental sustainability perception	Yes - Investigates hazelnut skins (a by-product classified as waste) as alternative animal feed to promote circular economy principles and reduce the environmental impact of livestock production.	K-means cluster analysis yielded five consumer segments with varying acceptance levels; age and sustainability sensitivity significantly predicted acceptance, with the "Informed, curious, and concerned" segment reporting the highest acceptability and sustainability scores.
48	Gärtner et al. (2024)	N=123, Germany, Sweden, Iceland, Consumers	Consumer acceptance test (Central Location Test); Kruskal-Wallis test	Vegan spread powders containing torula yeast (single-cell protein)	Trust in stakeholders/food industry, food neophobia (FNS-A), sensory perception	Overall sensory acceptance / Liking	Yes - Explores torula yeast (single-cell protein) as a highly scalable, efficient, and sustainable alternative protein source produced with a low environmental footprint and utilizing side-stream agro-industrial products.	Both vegan spreads received favorable ratings ("like slightly"), with the "Balkan"-style spread scoring higher; Icelandic consumers showed higher neophobia and higher trust in the food chain compared to German and Swedish consumers, who felt transparency was lacking.
49	Hopkins et al. (2023)	N=601, Australia, Adults	Online survey; Chi-square, Spearman's rank correlation; Food Neophobia Scale (FNS)	Edible insects as food	Food neophobia status, dietary choices (vegan, vegetarian, flexitarian, omnivore), protein food source habits, motivating factors	Previous insect-eating experience, future willingness to eat insects	Yes - Evaluates entomophagy as a sustainable solution to meet expanding global protein demand while reducing land, water, energy use, and greenhouse gas emissions.	Food neophobia was strongly associated with dietary choices (vegans/vegetarians exhibiting higher neophobia) and significantly decreased both previous insect-eating experience and future willingness to eat insects; nutritional benefits and increased accessibility were primary motivators.
50	Chen (2025)	N=48 (focus groups) & N=490	Mixed-methods; Moderated regression analysis	Plant-based meat (PBM)	Social representations theory, perceived benefits, perceived	Willingness to try	Yes - Examines plant-based meat as a novel sustainable food product	Perceived benefits, supply chain trust, and social norms positively contributed to

		(questionnaire), Taiwan, Consumers			trust in supply chain, perceived social norms, food neophobia		aligned with environmental and animal welfare considerations.	willingness to try plant-based meat, while food neophobia inhibited it and moderated the effect of social norms and perceived benefits.
51	Puleo et al. (2025)	N=164, UK, Consumers	Central Location Test (tasting); ANOVA & t-tests; Food Neophobia Scale (FNS)	Cricket-fortified biscuits	Food neophobia; Educational messaging; Attitudes toward insects	Hedonic liking / Acceptance	Yes - Entomophagy is highlighted as a sustainable solution that utilizes less land/water and emits fewer greenhouse gases than conventional livestock.	A 10% cricket-fortified biscuit was moderately accepted, but food neophobia significantly reduced hedonic liking; educational messaging failed to improve overall sensory hedonic responses.
52	Rodrigues Romano et al. (2025)	N=543 (Study 1) & N=805 (Study 2), Brazil, Consumers	Online experiment; OLS regression & Moderated mediation; Food Neophobia Scale (FNS)	Insect-based biscuits	Food neophobia; Goal framing (health vs. hedonic); Perceived sustainability; Visual imagery	Purchase intention	Yes - Insects are promoted as an environmentally sustainable substitute for meat, requiring less water and land while generating fewer greenhouse gas emissions.	Health goal framing effectively increased purchase intentions and perceived sustainability, successfully mitigating the negative impact of food neophobia on the intention to purchase insect-based biscuits.
53	Grahl et al. (2020)	N=420, Germany, France, Netherlands, Consumers	Central Location Test (tasting); Linear mixed effects models; Food Neophobia Scale (FNS)	Spirulina-filled pasta (alternative protein)	Food neophobia; Familiarity; Expectation-experience gap; Flavor masking	Overall liking; Expected liking	Yes - Spirulina is presented as a sustainable alternative protein source to facilitate a dietary shift, reducing energy, land, and water use compared to meat-based diets.	Increased spirulina content decreased liking, but a lemon-basil flavor successfully masked off-flavors; food neophobia negatively impacted expected liking conceptually, while familiarity with spirulina improved acceptance.
54	Brunner et al. (2025)	N=188, Switzerland, University students and employees (young adults)	Experiment (Tasting session); Single Category Implicit Association Test (SC-IAT); Multiple linear regression; Food Neophobia Scale (FNS)	Insect-based foods (cricket powder protein bar)	Food neophobia; Implicit attitudes; Green consumption values; Emotional responses; Sensory liking; Information provision (nutritional, environmental, taste)	Interest in entomophagy	Yes - Entomophagy is evaluated as a sustainable alternative protein source that utilizes significantly less land, water, and feed, and emits fewer greenhouse gases, contributing to a circular economy.	Green consumption values and positive emotional/sensory responses strongly predicted interest in entomophagy, while high food neophobia and negative implicit attitudes were significant barriers; informational interventions alone had no significant effect.
55	Etter et al. (2024)	N=916, Switzerland, Adults (Omnivores and Flexitarians)	Online survey; PCA & Multiple regression analyses; Food Neophobia Scale (FNS)	Meat alternatives (17 alternative protein sources including plants, algae, insects, and cultured meat)	Food neophobia; Environmental identity; Health consciousness; Preference for naturalness; Meat alternative rejection	Acceptance / Willingness to consume	Yes - Meat alternatives are investigated to address the severe environmental damages of meat production, including deforestation, biodiversity loss, and greenhouse gas emissions.	Food neophobia significantly lowered the acceptance of plant-based proteins, algae, and insects, while strong environmental identity and health consciousness increased their acceptance; cultured meat was predominantly hindered by a preference for naturalness.
56	Sogari et al. (2023)	N=3421, Belgium, China, Italy, Mexico, USA, Adults	Online cross-sectional survey; Multigroup structural equation modeling (M-SEM); Food Neophobia Scale (FNS), Entomophagy Attitude Questionnaire (EAQ)	Edible insects (whole and processed)	Food neophobia; Disgust (EAQ sub-dimension); Prior tasting experience	Willingness to engage (eat and purchase) (WTE)	Yes - Entomophagy is framed as a traditional and novel practice requiring fewer resources and offering sustainable alternatives to the Global North's conventional food supply.	Food neophobia and insect-specific disgust significantly hindered the willingness to engage in entomophagy; notably, food neophobia was a stronger predictor of rejection for those who had previously eaten insects than for non-eaters, while disgust affected both groups equally.
57	Peressi & Simonetti (2026)	N=413, Germany and Italy, Adults	Online survey/simulated online supermarket choice task; Mediation/moderation analysis & Mixed logit models; Food Neophobia Scale (FNS), Food Technology Neophobia Scale (FTNS)	Precision-fermentation dairy products (cheese, yoghurt, milk, ice cream)	Expected sensory quality, Food neophobia (FN), Food technology neophobia (FTN)	Choice / Willingness to buy	Yes - Precision fermentation is evaluated as a sustainable alternative that eliminates the need for livestock, reducing environmental impacts and greenhouse gas emissions.	Conventional dairy was consistently preferred over precision-fermentation alternatives; expected sensory quality strongly mediated this choice, while food technology neophobia (but not general food neophobia) significantly dampened sensory expectations and reduced the likelihood of choosing precision-fermentation products.
58	Vermetten et al. (2026)	N=402, Spain and the Netherlands, Young adults (18-40 years)	Online survey; Linear regression & Pearson correlations; Food Neophobia Scale (FNS), Food Technology Neophobia Scale (FTNS)	Novel alternative proteins (mealworm and mung bean protein powders)	Food neophobia, Food technology neophobia, Health claims	Willingness to try (WTT)	Yes - Adopting alternative proteins like insects and plant-based proteins is framed as crucial to reducing the environmental impact of traditional animal-based proteins and promoting sustainable food systems.	Willingness to try alternative proteins was inversely correlated with both food neophobia and food technology neophobia; consumers showed much higher acceptance for plant-based proteins than insect proteins, and the addition of protein-related health claims did not significantly improve willingness to try.
59	Orkusz et al. (2020)	N=866 (N=464 survey, N=402 tasting session), Poland, University students / Adults	Survey and tasting session; Binomial logistic regression & Chi-square tests; Food Neophobia Scale (FNS)	Edible insects (whole and processed into bread/biscuits)	Food neophobia, Demographics (sex, travel history), Knowledge, Environmental concern	Acceptance / Willingness to consume	Yes - Edible insects are positioned as an environmentally friendly alternative to meat with lower greenhouse gas emissions, less water use, and a smaller land footprint.	Food neophobia was the strongest barrier to acceptance, with neophobic individuals highly unlikely to accept insects; willingness to eat insects was significantly higher when they were processed invisibly into familiar foods (bread), while environmental benefits were the least influential motivator for consumption.
60	Torri et al. (2020)	N=1445, Italy, Adults	Online survey; Hierarchical multiple linear regression & Correspondence Analysis; Food Neophobia Scale (FNS)	Jellyfish (whole, processed, varying culinary preparations)	Food neophobia, Disgust sensitivity, Jellyfish consumption attitude (JCA), Socio-demographics, Culinary contexts	Willingness to eat / Willingness to buy	Yes - Jellyfish are framed as an abundant, sustainable novel food source that could reduce environmental impact, diversify fisheries, and help food systems adapt to climate change and overfishing.	Food neophobia and disgust sensitivity were strong negative predictors of the willingness to consume jellyfish; however, acceptance significantly increased when jellyfish were processed into invisible forms (rather than whole), paired with familiar savory flavors, or evaluated by young, frequent travelers.
61	Miglietta et al. (2025)	N=380, Italy, Adults	Quali-quantitative online survey; Free word association	Insect-based foods (insect flour/pasta)	Disgust, Food neophobia, Positive moral attitude, TPB	Behavioral Intention (Buy/Eat)	Yes - Insect-based foods are evaluated as sustainable protein sources	Three distinct consumer segments emerged: one driven by disgust (rejecting insects

			(ALCESTE) & Theory of Planned Behavior (TPB) modeling; Food Neophobia Scale (FNS-R)		variables (Subjective norms, Behavioral control)		requiring less water/soil and generating fewer greenhouse gases than conventional livestock, aligning with FAO environmental goals.	entirely), one driven by sustainability and nutrition (accepting insects), and a cautious but curious group; while disgust strongly predicted rejection, positive moral attitudes toward the environment drove acceptance, highlighting the need for targeted informational strategies.
62	Schlup & Brunner (2018)	N=379, Switzerland, Adults	Mail survey; Tobit regression; Food Neophobia Scale (FNS)	Edible insects / Insect-based food	Food neophobia, Food technology neophobia, Insect salience (visibility), Familiarity, Prior consumption	Willingness to consume (WTC)	Yes - Entomophagy is framed as a sustainable, eco-friendly alternative to meat that requires less feed, space, and produces fewer greenhouse gas emissions, addressing climate change and food scarcity.	Food neophobia and food technology neophobia significantly reduced willingness to consume insects, though food neophobia was no longer the sole primary barrier; acceptance increased significantly when insects were invisible (low salience) and when consumers had prior tasting experience.
63	Maciejewska et al. (2025)	N=140, Poland and Spain, Consumers	Online survey; Mixed model ANOVA; Food Neophobia Scale (FNS), Insect Phobia Scale (INS), Entomophagy Scale (ENS)	Edible insects / Tenebrio molitor (yellow mealworm) hydrolysates	Food neophobia, Insect phobia, Entomophagy attitudes, Demographics (age, gender, education)	Willingness to try / Acceptance	Yes - Insects are presented as a sustainable alternative protein source capable of reducing greenhouse gas emissions and environmental degradation associated with meat production.	Spanish respondents exhibited a higher baseline acceptance and lower neophobia than Polish respondents, though both groups showed increased willingness to try when informed about the nutritional benefits and safety of insect-based products; overall, familiarity and education were key drivers of acceptance.
64	Laureati et al. (2016)	N=341 (survey) and N=68 (hedonic test), Italy, Adults	Online survey & visual hedonic assessment; Generalized Linear Model (GLM) ANOVA; Food Neophobia Scale (FNS)	Insect-based foods and feed (visible, invisible, sweet, savory)	Food neophobia, Sustainability behavior/awareness, Information provision, Socio-demographics (gender, age, university affiliation)	Willingness to accept / Expected liking	Yes - Insects are described as providing sustainable animal proteins with a higher feed-conversion efficiency, lower greenhouse gas emissions, and lower land/water requirements than conventional livestock.	Food neophobia was the strongest barrier to accepting insects as food or feed, with neophobic individuals showing high rejection rates; providing information on environmental and nutritional benefits only marginally increased the expected liking of insect-based foods, and surprisingly, consumers' personal involvement in sustainability issues did not increase their willingness to eat insects.
65	Kasana et al. (2025)	N=400, Indonesia (East Java), Consumers (>15 years old)	Online survey; Structural Equation Modeling-Partial Least Squares (SEM-PLS); Modified neophobia measurement	Microalgae (as an alternative protein source)	Environmental concern, Health interest, Food neophobia, Knowledge, Attitude, Self-efficacy	Willingness to try	Yes - Microalgae are framed as a sustainable alternative protein source that provides nutritional benefits for humans and acts as a carbon sink (utilizing CO2) to benefit the environment.	Environmental concern, health interest, and knowledge significantly and positively influenced consumer attitudes toward microalgae, which in turn strongly predicted willingness to try; conversely, food neophobia had a negative but statistically insignificant effect on attitudes.
66	Costa et al. (2025)	N=946, Sweden, Adults (Omnivores, Flexitarians, Pescatarians)	Online survey; Logistic regression, Multiple linear regression, Mediation analysis; Alternative Food Neophobia Scale (FNS-A)	Seafood (salmon, herring, oysters, seaweed, octopus)	Food neophobia, Sensory expectations (appearance, aroma, taste, texture), Emotional arousal, Familiarity	Expected liking	Yes - Seafood (especially oysters and seaweed) is positioned as an alternative food source that could potentially reduce demand for terrestrial animal-source foods due to its lower environmental impact.	Higher food neophobia significantly decreased expected liking for all seafood types except the highly familiar salmon, with this relationship partially mediated by increased emotional arousal; individuals with higher food neophobia were also more likely to expect to dislike all sensory modalities (appearance, aroma, taste, and texture) prior to tasting.
67	Barton et al. (2020)	N=107 (survey) and N=102 (acceptability trial), Canada (Atlantic region), Adults	Survey & Consumer acceptability trial (focus group + sensory tasting); Paired t-tests & Penalty analysis; Food Neophobia Scale (FNS)	Cricket (Acheta domesticus)-based protein powders	Food neophobia, Specific Beliefs and Attitudes about Insect Consumption (BAIC), Sensory properties	Acceptance / Willingness to buy / Liking	Yes - Insects are described as a sustainable protein source that provides high energy with lower requirements for land and water, and lower greenhouse gas emissions compared to farm animals.	Before the trial, participants believed eating insects was disgusting and unnatural; however, after blindly tasting chocolate and original protein powders containing 30% cricket powder (which did not negatively impact sensory liking), participants' disgust significantly decreased, and their willingness to buy and recommend cricket powder significantly increased.
68	Niva & Vainio (2021)	N=1000, Finland, Adults (18-79 years)	Online survey; Latent class analysis (LCA) & Multinomial logistic regression; Food Neophobia Scale (FNS)	Beef, plant-based protein products, and insect-based products	Food neophobia, Eating motives (sustainability, health, pleasure, naturalness, price), Sociodemographics	Past changes and future intentions to consume	Yes - Transitioning from beef to plant- and insect-based proteins is framed as necessary to mitigate climate change and reduce environmental impacts.	Five consumer clusters were identified based on consumption changes; increasing the use of alternative proteins was significantly associated with lower food neophobia and higher health and sustainability motives, while established beef lovers were highly neophobic and prioritized pleasure.
69	Cicatiello et al. (2020)	N=62, Italy, Young adults / University students (18-35 years)	Tasting panel and questionnaire; Analysis of covariance (ANCOVA); Food Neophobia Scale (FNS)	Insect-based snacks (cricket chips, whole dried crickets, cricket flour chocolate bar, whole dried mealworms)	Food neophobia, Product features (visible vs. invisible insects, sweet vs. savory), Food preferences, Demographics	Overall liking (Sensory evaluation)	Yes - Entomophagy and insect rearing are positioned as a sustainable alternative protein source with a lower environmental impact and high feed conversion efficiency.	Overall sensory liking for the insect-based snacks was high, especially for chocolate bars with invisible insect flour; however, higher food neophobia, a desire to know all ingredients, and the visibility of whole insects significantly reduced overall sensory liking.
70	Stanco et al.	N=437, Italy, Meat	Online survey; Logistic	Cultivated meat	Food technology neophobia,	Willingness to purchase	Yes - Cultivated meat is positioned as	Willingness to purchase cultivated meat was

	(2025)	consumers (Adults)	regression & Word cloud analysis; Abbreviated Food Technology Neophobia (AFTN) scale		Green Consumer Value (GCV), Health consciousness, Ethical motives, Innovation-seeking		a sustainable technology that reduces the environmental footprint of traditional farming, greenhouse gas emissions, and land/water use.	low (35.5%); food technology neophobia and skepticism significantly reduced purchase likelihood, whereas strong green consumer values (sustainability) and awareness of cultivated meat positively drove consumer acceptance.
71	Videbæk & Grunert (2020)	N=975, Denmark, Adults	Online questionnaire & Discrete choice experiment; Latent class choice model & Hierarchical regression; Food Neophobia Scale (FNS), Disgust Sensitivity Scale	Edible insects (whole and as an ingredient)	Food neophobia, Disgust sensitivity, Food neophilia, Attitude ambivalence (interest vs. disgust)	Choice / Intention to eat	Yes - Insects are presented as a sustainable alternative to meat that can greatly benefit the environment and diminish greenhouse gas emissions.	Three consumer segments were identified (Insect Opponents, Insect Feeders, Potential Entomophagists); while food neophobia and disgust were strong barriers, the intention to eat insects was primarily driven by interest, and all segments exhibited attitudinal ambivalence (tension between positive and negative evaluations).
72	Kamin et al. (2024)	N=286 (subset of flexitarians from N=1023), Slovenia, Young adults (18-35 years)	Online survey; Hierarchical and K-means clustering & t-tests; Food Neophobia Scale (FNS)	Meat alternatives (plant-based meat, cultured meat, insects)	Food neophobia, Environmental awareness, Meat attachment, Social norms, Self-efficacy, Subjective health	Attitudes and willingness to try/replace	Yes - Reducing meat consumption and adopting flexitarian diets/alternative proteins is positioned as crucial for protecting the environment and building sustainable food systems.	Flexitarians segmented into Ethical and Utilitarian groups; Utilitarian flexitarians exhibited higher food neophobia and meat attachment, while Ethical flexitarians showed higher environmental awareness and significantly more positive attitudes and willingness to adopt plant-based and cultured meat (though both groups rejected insects).
73	Mazurek et al. (2024)	N=106, Poland, Adults (20-27 years)	Sensory evaluation & Online survey; Multiple regression & Kruskal-Wallis tests; Food Neophobia Scale (FNS)	Muffins containing 15% insect powder (sweet and savory)	Food neophobia, Consumer attitudes, Sensory characteristics (appearance, smell, taste, texture)	Acceptance / Sensory rating	Yes - Edible insects are promoted as an alternative protein source with a lower environmental impact, highly efficient metabolism, and reduced greenhouse gas emissions compared to traditional livestock farming.	Overall acceptance of insect muffins was lower than control muffins, with sweet versions generally preferred over savory; food neophobia significantly hindered acceptance, while taste and texture were the most critical sensory predictors driving consumer acceptance or rejection of the products.
74	Salgaonkar & Nolden (2024)	N=454, USA, Adults	Online survey; Choice-Based Conjoint Analysis (CBC) & Multiple linear regression; Food Neophobia Scale (FNS)	Hybrid hotdogs (50% plant-based / 50% beef)	Food neophobia, Meat attachment, Ethical/Moral/Social/Health motives, Product attributes (protein, fat, price, claims)	Willingness to purchase / Product selection	Yes - Plant-based and hybrid meat alternatives are framed as strategies to reduce animal meat consumption in support of environmental sustainability, animal welfare, and reduced ecological burden.	Hybrid hotdogs were the least preferred option compared to 100% beef and 100% plant-based alternatives; surprisingly, psychological factors like food neophobia, meat attachment, and ethical motives did not significantly influence the choice of hybrid hotdogs, suggesting a lack of consumer familiarity and understanding of this new category.
75	Fantechi et al. (2026)	N=500, Italy, Meat consumers (Adults)	Online survey; Structural equation modeling (SEM) & Seemingly unrelated regression (SUR); Food Neophobia Scale (FNS)	Alternative proteins (seitan, insects, cultured meat)	Food neophobia, Food technology neophobia, Norm Activation Model (awareness of consequences, ascription of responsibility, personal norms), Values (sustainability, health, animal welfare)	Intention to engage / try / Frequency of consumption	Yes - Alternative proteins are presented as substitutes for conventional meat necessary to address global challenges including environmental sustainability, public health, and animal welfare.	Awareness of environmental impacts and involvement in sustainable eating strongly predicted personal norms driving the intention to try alternative proteins; however, food neophobia (for insects) and food technology neophobia (for cultured meat) acted as significant barriers to engagement.
76	Ramaroson et al. (2026)	N=127 adults and N=67 children, Madagascar (rural southeast), Consumers	Mixed methods: Survey & Focus Group Discussions (FGDs); ANOVA, Student's t-test, Chi-square & Factorial Correspondence Analysis; Food Neophobia Scale (FNS)	Black soldier fly (BSF) larvae products (whole larvae, powder, crackers)	Food neophobia, Sensory preferences, Cultural beliefs, Demographics	Acceptance / Product preference / Willingness to incorporate and produce	Yes - Black Soldier Fly larvae are promoted as a sustainable protein alternative with a minimal environmental footprint that can upcycle organic waste and reduce dependence on environmentally damaging bushmeat.	While participants showed moderate food neophobia, direct consumption of BSF products significantly reduced disgust and increased environmental awareness; processed forms (crackers) were highly accepted by both adults and children, whereas whole larvae were rejected due to visual disgust and texture.
77	Kamei et al. (2024)	N=1079 (main survey) + 909 (other phases), Japan, Adults (20-69 years)	Online survey; Confirmatory Factor Analysis (CFA), ANOVA, Correlation analysis; Japanese version of the Alternative Food Neophobia Scale (J-FNS-A)	Hamburgers with alternative protein patties (soy meat, cultured meat, cricket, algae, mealworm)	Food neophobia (J-FNS-A: approach and avoidance factors), Familiarity, Demographics	Willingness to eat (WTE)	Yes - Alternative protein foods are described as sustainable resources needed to shift dietary styles, reduce environmental impacts (carbon and water footprints), and address food security.	The newly developed Japanese Alternative Food Neophobia Scale (J-FNS-A) demonstrated that Japanese consumers have relatively high food neophobia scores; higher neophobia was significantly associated with a lower willingness to eat hamburgers made with unfamiliar alternative proteins, and this negative effect strengthened as familiarity decreased.
78	Chezan et al. (2022)	N=102 (survey) and N=6 (interviews), Europe (mostly UK), Adults	Mixed methods: Online survey & Semi-structured interviews; Linear regression & Mann-Whitney tests; Food Technology Neophobia Scale (FTNS)	Fungal protein (mycoprotein) meat substitutes	Food Technology Neophobia, Perceived benefits (health, naturalness, sustainability), Familiarity, Mold association, Demographics	Purchase intentions (PI) / Willingness to try	Yes - Fungal protein (mycoprotein) is presented as a sustainable alternative that can facilitate sustainable diets without compromising animal welfare and has important environmental advantages compared with conventional meat.	Although participants recognized the societal and environmental benefits of fungal protein, actual purchase intentions were low due to poor sensory expectations and the stigma/disgust associated with mold; food technology neophobia was a significant but weak predictor of lower perceived benefits and purchase intentions.

79	Kane & Dermiki (2021)	N=268, Ireland, Adults (Staff and students at an institute of technology)	Online survey; Non-parametric tests (Mann-Whitney, Kruskal-Wallis, Spearman's correlation); Food Neophobia Scale (FNS)	Entomophagy / Willingness to consume insects	Sociodemographics, previous knowledge/experience, food neophobia, meat attachment, health score, environmental concern	Willingness to try insects and conditions for consumption	Yes - Entomophagy is being explored as a sustainable food source in Western countries to combat climate change, owing to lower greenhouse gas emissions and reduced resource, transport, water, and land use compared to animal proteins.	Less neophobic males who did not follow specific diets were the most receptive to entomophagy, while food neophobia, disgust, and safety concerns acted as major barriers; participants were much more willing to consume insects disguised in familiar foods rather than whole, or indirectly by feeding insects to livestock.
80	Cascone et al. (2026)	N=415, Italy, Physically active consumers (amateur athletes and gym users)	Online survey; Partial Least Squares Structural Equation Modelling (PLS-SEM); Food Neophobia Scale (FN) and Food Technology Neophobia (FTN)	Insect-based protein supplements	Food Neophobia (FN), Food Technology Neophobia (FTN)	Consumption intention, Perceived benefits (PB), Perceived risks (PR)	Yes - Insect-based proteins are framed as a strategy to transition toward more resilient and sustainable agri-food systems, offering high feed conversion efficiency and a low environmental footprint.	Perceived benefits (such as functional, nutritional, and performance-related outcomes) were the strongest positive drivers of consumption intention, outweighing perceived risks; Food Neophobia and Food Technology Neophobia both reduced consumption intention by indirectly diminishing perceived benefits and amplifying perceived risks.
81	Hoek et al. (2011)	N=553, UK and the Netherlands, Adults	Cross-national consumer survey; Categorical regression (CATREG) & ANOVA; Food Neophobia Scale (FNS), Food Choice Questionnaire (FCQ)	Meat substitutes (plant-based, mycoprotein)	Food neophobia, Food choice motives (ecological welfare, health, convenience), Product familiarity, Attitudes toward meat	Acceptance / Usage frequency (Non-users, light/medium-users, heavy-users)	Yes - Meat substitutes are framed as sustainable alternatives necessary to reduce the negative environmental impact of industrial-scale meat production.	Food neophobia, lack of familiarity, and inferior sensory expectations were the main barriers to meat substitute acceptance for non-users; conversely, heavy users were primarily driven by ecological welfare and ethical motives, whereas non-users strongly favored conventional meat for its sensory qualities.
82	Miller et al. (2025)	N=187, USA, Midwestern college students (Generation Z)	Cross-sectional online survey; Welch's t-tests & Descriptive statistics; Food neophobia questions included (no validated FNS reported)	Edible insects	Knowledge (environment, nutrition, health domains), Willingness to consume, Food neophobia, Demographics	Willingness to try / Willingness to consume	Yes - Edible insects are described as a sustainable alternative protein source with significantly lower carbon emissions, feed, and water requirements compared to traditional livestock, addressing climate change and resource scarcity.	Most Gen Z students were reluctant to eat insects primarily due to disgust and food neophobia, coupled with a general lack of knowledge about insect sustainability and safety; however, over half expressed a conditional willingness to try them if their sustainability benefits were emphasized.
83	Jensen & Lieberoth (2018)	N=189, Denmark, College students	Online survey & Surprise tasting session; Multivariate regression & Partial correlations; Food Neophobia Scale (FNS)	Edible insects (Roasted mealworms, visible vs. invisible)	Food Neophobia, Pathogen Disgust Sensitivity, Perceived Infectability, Perceived social norms, Insect eating disgust	Willingness to eat (WTE) / Actual tasting behavior	Yes - Incorporating insects into Western food culture is framed as a way to address global challenges such as global warming, deforestation, species extinction, and depleted fresh water supplies.	Food neophobia and insect-specific disgust were significant barriers to willingness to eat, particularly when insects were visible; however, actual tasting behavior during a collective session was primarily predicted by perceived social norms rather than trait-level disgust or fear of contamination.
84	Tsimitri et al. (2021)	N=700 (Greece n=384, Cyprus n=222, Uganda n=94), Consumers (Urban areas)	Cross-national survey; Two-step cluster analysis (TSCA), Categorical regression (CatReg); Food Neophobia Scale (FNS), Food Technology Neophobia Scale (FTNS)	Functional yogurt-like product (derived from halloumi cheese whey enriched with banana by-products)	Food neophobia, Food technology neophobia, Socio-demographics (age, gender, income, education, marital status, family size)	Acceptance / Rejection / Willingness to consume / Neophobic tendency	Yes - The novel yogurt is framed as exploiting agri-food industrial by-products (whey and banana waste) to promote bioeconomy, circular economy, and reduce environmental impact.	Three clusters of neophobic tendency were identified (neophilic, neutral, neophobic), with respondents generally showing low-to-neutral neophobia and high acceptance of the novel yogurt; technophobia was a key driver of rejection in Europe, while food phobia was stronger in Uganda; higher education and younger age were associated with lower neophobia and higher acceptance.
85	Mustapa & Kallas (2023)	N=1260, Spain, Adults	Online survey; Kruskal-Wallis and Mann-Whitney U tests; Food Neophobia Scale (FNS), New Ecological Paradigm (NEP) Scale	Animal products (pork, chicken, eggs, fish) fed with insects	Food neophobia, Food neophilia, Ecocentric and anthropocentric environmental attitudes (NEP), Opinions on the use of insects in animal feed, Sociodemographics	Willingness to consume (WTC) insect-fed animal products	Yes - Using insect meal for animal feed is framed as a sustainable alternative that reduces land/water use, greenhouse gas emissions, and waste, contributing to a circular economy.	Most Spanish consumers expressed high uncertainty regarding consuming animal-fed insect meals but showed a high ecocentric attitude; women were more willing to consume sustainable animal products than men despite higher neophobia; food neophobia negatively impacted willingness to consume, while food neophilia and ecocentric attitudes positively influenced acceptance.
86	Niimi et al. (2022)	N=101 (consumer test) and N=288 (online survey), Sweden, Consumers	Central location test (CLT) & Online survey; ANCOVA, Cochran's Q test, Multiple factor analysis for contingency tables (MFACT), Check-all-that-apply (CATA); Food Neophobia Scale (FNS), Cooking and Food Provisioning Action Scale (CAFPAS)	Bolognese sauce made with beef vs. meat substitutes (soy, mycoprotein, oat)	Cooking ability (CAFPAS), Food neophobia, Sensory attributes (CATA), Demographics	Liking (overall, appearance, aroma, taste/flavour, texture), Sensory discrimination	Yes - Meat substitutes are framed as new and sustainable food products necessary to mitigate the food industry's climate impact.	Cooking ability and food neophobia were negatively correlated; meat substitutes could be liked as much as or more than beef in a familiar meal context (Bolognese); cooking ability did not strongly affect sensory discrimination or overall liking, but food neophobia significantly impacted hedonic ratings.
87	Dean et al. (2024)	N=4488, Cross-national (12 countries including	Online/paper survey; Partial least squares structural equation modeling (PLS-SEM);	Cultured muscle, plant-based protein, and fungal-based protein	Food neophobia, Food technology neophobia, Environmental and health	Perceived suitability/benefits, Willingness to try, buy, and pay more	Yes - Alternative proteins are presented as ways to decrease energy footprints, greenhouse gas emissions,	The largest drivers of willingness to consume meat substitutes were their perceived suitability/specific benefits and environmental

		UK, Pakistan, China, USA, France, etc.), Consumers	Food Neophobia Scale (FNS), Food Technology Neophobia (FTN) scale		impact on food choice, Meat nutritional importance, Meat sensory importance		water use, and avoid factory farming/animal cruelty.	impact; the biggest inhibitors were food neophobia, food technology neophobia, and the perceived nutritional/sensory importance of conventional meat.
88	Lanz et al. (2025)	N=566, Germany, Adults (18-35 years, 50% vegetarians/vegans, 50% omnivores/flexitarians)	Online survey; Multiple linear regressions, t-tests, chi-squared tests; Food technology neophobia scale, Food Choice Questionnaire (FCQ) naturalness	Cultured meat (cultured burger vs. conventional and plant-based burgers)	Food technology neophobia, Environmental attitude, Health consciousness, Meat alternative rejection, Importance of food naturalness	Perception as vegetarian, Willingness to eat, Perception of benefits, Perceived sensory/health/environmental attributes	Yes - Cultured meat is framed as an innovation that can reduce land/water usage, generate fewer greenhouse gas emissions, and cause less harm to animals compared to traditional livestock farming.	Cultured meat presented a conflict for vegetarians: they recognized its environmental/animal welfare benefits more than omnivores but were less willing to consume it due to uncertainty over whether it aligns with vegetarianism; food technology neophobia negatively predicted willingness to eat across both dietary groups.
89	Rombach et al. (2022)	N=3091, Cross-national (China, USA, France, UK, New Zealand, Netherlands, Brazil, Spain, Dominican Republic), Consumers	Online/paper survey; Partial least squares structural equation modeling (PLS-SEM); Food neophobia, Food technology neophobia, Food choice questionnaires	Cultured meat	Food curiosity, concerns about food technology, food neophobia, meat importance, perception of cultured meat as a realistic alternative, food allergies, locavore tendencies, demographics	Willingness to try, buy, and pay a price premium for cultured meat	Yes - Cultured meat is enjoyed as a more sustainable meat option that addresses negative externalities associated with livestock production like water depletion, climate change, and biodiversity loss.	Food neophobia, food technology neophobia, food allergies, and locavore tendencies were inhibiting factors, whereas food curiosity, meat importance, and perceiving cultured meat as a realistic alternative were key drivers of willingness to try, buy, and pay a price premium.
90	Cavallo et al. (2025)	N=798, Italy, Adults (National representative sample)	Online survey; Confirmatory Factor Analysis (CFA) & Nested Structural Equation Models (SEM); Food Choice Questionnaire (FCQ), Food Neophobia Scale (FNS)	Sushi (raw/undercooked fish)	Food choice motives (FCQ), food neophobia, social norms, traceability, sushi consumption frequency	Sushi consumption frequency	Yes - Mentions sushi and alternative food trends as part of sustainable seafood choices and broader dietary shifts towards sustainable food systems.	Social norms were the strongest positive drivers of sushi consumption, while sensory appeal, price, food neophobia, and fish traceability negatively impacted consumption frequency, indicating that Italian consumers value safety, naturalness, and traceability when breaking traditional food taboos regarding raw fish.
91	Clarkson et al. (2018)	N=32, New Zealand (Dunedin), Adults (Students and staff participating in product design workshops)	Mixed methods: Focus group discussions & Consumer idealised design workshops; Thematic qualitative analysis	Insect protein-based food and liquid products (using extracted locust protein)	Consumer attitudes, drivers and barriers (food neophobia, disgust sensitivity, culture), ideal product attributes (4P's: product, promotion, price, place)	Consumer acceptance / Ideal product attribute design	Yes - Insect production is highlighted as a highly sustainable practice requiring significantly less feed, land, and water, and producing fewer greenhouse gas emissions compared to livestock farming.	Participants experienced both disgust and intrigue, citing culture, food neophobia, and lack of necessity as barriers, while novelty, health, and sustainability were drivers; participants overwhelmingly designed insect protein as convenient sweet snacks, drinks, or breakfast options (rather than meat substitutes) packaged attractively with cartoon insect imagery for children or fitness-oriented consumers.
92	Junges et al. (2021)	N=14 interviews + N=410 survey respondents, Brazil (Rio Grande do Sul state), Consumers	Mixed methods: Semi-structured interviews & Online survey; Factor analysis & Two-Step Cluster Analysis; Food Neophobia Scale (FNS), Semantic differential scale	Insect-based foods (cereal bars, breaded steak, pasta made with insect flour)	Food neophobia, attitudes toward insect-based foods, meat consumption habits and reduction intentions, marketing attributes (appearance, price, packaging, variety, accessibility)	Consumer attitude / Willingness to consume / Market segmentation	Yes - Insect-based foods are promoted as a sustainable alternative protein source that requires fewer resources and causes less environmental impact than traditional animal protein production.	Two distinct consumer segments were identified ("favorable attitude" [44.9%] and "unfavorable attitude" [55.1%]); food neophobia strongly hindered acceptance, with cereal bars receiving the highest acceptance and breaded steak the lowest, while appearance and price emerged as critical marketing attributes for market success.
93	Gómez-Luciano et al. (2021)	N=401 (Spain n=200, Dominican Republic n=201), Adults	Cross-national survey; Principal Component Analysis (PCA) & Binary logistic regression; Food Neophobia Scale (FNS), Food Technology Neophobia Scale (FTNS)	Insects as food	Perception of insects as food, Food Neophobia Scale (FNS), Food Technology Neophobia Scale (FTNS), willingness to try and buy	Willingness to accept / try / buy insects as food	Yes - Insects are presented as a sustainable source of protein to address increasing food demand and mitigate climate change/environmental pressures.	Dominicans exhibited the highest food neophobia and lowest acceptance, whereas Spanish consumers showed higher openness; openness to "eat almost anything" and overstating the benefits of new food technologies were key positive predictors of acceptance, while disgust, neophobia, and fear of trying new foods served as major barriers.
94	Orsi et al. (2019)	N=393, Germany, Consumers	Online survey; Ordinal regressions (cumulative link models)	Edible whole insects and processed insect-based food (powder, extracts, bars, pasta, granola, burgers)	Food neophobia, disgust, environmental awareness, health consciousness, risk assessment, personal experiences, familiarity, sociodemographics	Acceptance / willingness to try whole insects (AccWhole) and processed insect products (AccProcess)	Yes - Insects are framed as a sustainable animal protein source requiring far fewer natural resources (water, feed, land) and causing significantly less greenhouse gas and ammonia emissions, fitting into the circular economy.	Willingness to try insects was low among Germans; food neophobia and disgust were the strongest negative barriers, with food neophobia highly significant for whole insects and disgust having a higher negative impact on processed products; health consciousness increased acceptance of processed products, while visibility of whole insects remained a major hurdle, making processed products a more promising market entry strategy.
95	Costa et al. (2023)	N=211, Sweden, Consumers	Central location tasting session & online pre-screening; Mixed ANOVA, Pearson correlations, multiple linear regression, mediation analysis; Food	Novel surimi-based products shaped to resemble pasta (Alaska pollock, cod, salmon variants)	Food neophobia, food technology neophobia, food agency (CAFPAS), emotional arousal, aroma/flavor intensity, hedonic liking,	Liking (overall, appearance, aroma, taste/flavour, texture), emotional arousal, purchase intention	Yes - Aquatic foods and surimi-based products utilizing aquatic side-streams are framed as supporting the transition away from terrestrial animal-source foods towards more	Negative associations were found between food neophobia and hedonic ratings/purchase intention; mediation analysis revealed that the relationship between food neophobia and hedonic liking was indirectly explained by

			Neophobia Scale (FNS), FNS-A, Food Technology Neophobia Scale (FTN), CAFPAS		purchase intention, information condition (blind vs. informed)		sustainable, low-environmental-impact protein sources.	emotional arousal (higher arousal leading to lower liking), supporting the arousal hypothesis; however, this effect was absent for familiar/popular salmon, highlighting that familiarity can offset neophobic aversion.
96	Tuccillo et al. (2020)	N=400 (survey) and N=58 (sensory evaluation), Italy, Adults	Online survey & sensory evaluation; t-tests, Pearson correlations, Cochran's Q test, Hierarchical cluster analysis, ANOVA; Food Neophobia Scale (FNS), Disgust Scale (DS), CATA for emotions	6 edible insects & 6 insect-based products/dishes (IBPD), plus 3 cricket visibility levels (whole, focaccia bits, crackers)	Sociodemographics, personality traits (food neophobia, disgust sensitivity), willingness to eat (WTE) specific species/stages/products, emotions (CATA), sensory liking and visibility levels	Willingness to eat (WTE) insects and insect-based products/dishes, emotional responses, sensory liking	Yes - Entomophagy is positioned as a sustainable practice that provides a reliable and sustainable alternative source of high-protein food to replace conventional meat and reduce environmental pressures.	Males were more positive towards insects, less influenced by species/processing, and more willing to eat for taste; crickets were the most accepted species, and adult insects were preferred over larvae; insect visibility negatively impacted acceptability, with invisible flour crackers preferred over whole crickets; three consumer clusters were identified ("In favour of eating insects" [41%], "Picky towards eating insects" [32%], and "Against eating insects" [27%]), driven mainly by food neophobia and disgust sensitivity.
97	Giezenaar et al. (2023)	N=572 (Millennials and Gen X), Aotearoa New Zealand (A-NZ), Consumers	Online survey; Linear models, Dunn tests, Principal Component Analysis (PCA), mixed-effect models, k-means cluster analysis; Food Neophobia Scale	Cultivated meat (CM) compared to conventional meat and plant-based meat alternatives (PBMAs)	Awareness of CM, potential engagement (taste, regular eating, perceived product attributes relative to conventional meat and PBMAs), demographics, meat/PBMA consumption frequency, food neophobia	Awareness, willingness to taste/eat regularly/purchase, perceived product attributes	Yes - Cultivated meat is framed as an alternative protein source that enhances personal health and environmental sustainability by reducing land/water usage and greenhouse gas emissions compared to livestock farming.	52% of respondents were aware of CM (higher in men, Millennials, and frequent PBMA consumers); CM aware respondents rated potential engagement and product attributes significantly higher; CM was perceived as more ethical, environmentally sustainable, and animal-friendly relative to conventional meat, and more natural/tasty relative to PBMAs; cluster analysis divided respondents into three groups: "positive" (41%, more aware, frequent PBMA consumers, lower meat intake), "neutral" (50%), and "negative" (9%).
98	Fasanelli et al. (2025)	N=295, Italy, Consumers	Mixed-methods survey (hierarchical evocation, food neophobia scale, checklists, open-ended questions, projective sensory analysis), lexical, prototypical, and similarity analysis (IRaMuTeQ)	Cultured (synthetic) meat	Social representations (central core and peripheral schemes), food neophobia, attitudes (SR_NFQ, CuMAQ), projective sensory experience	Social representations, attitudes, and willingness to consume cultured meat	Yes - Cultured meat is framed as an alternative protein source addressing environmental impacts, intensive farming, and sustainability.	Social representations of cultured meat differ between omnivores and vegetarians/vegans; omnivores' core representations center on sustainability, innovation, and future, while vegetarians focus on sustainability, ethics, and science; food neophobia, technology neophobia, and disgust act as barriers, and sensory/cognitive anchors shape willingness to consume.
99	Zhang et al. (2026)	N=574, UK, Meat eaters (omnivores and flexitarians)	Online survey with written shopping scenario & optional environmental information video; Mixed model ANOVA, correspondence analysis, Cochran's Q, cluster analysis (Ward's method); Food Neophobia Scale (FNS), Food Technology Neophobia Scale (FTNS), Food Disgust Scale (FDS)	Burger patties (conventional beef, plant-based, lab-grown beef, edible insect)	Expected overall liking, purchase intention, emotional responses (CATA), personal traits (FNS, FTNS, FDS clusters), information sharing (video)	Expected liking, purchase intention, emotional response, final burger choice	Yes - Alternative proteins and protein transition are framed as strategies to reduce greenhouse gas emissions and improve the sustainability of the food system.	Conventional beef was most liked and chosen, followed by plant-based and lab-grown beef, with edible insects least liked; four personal trait clusters were identified (food explorers, food tech fearers, novel/disgust fearers, everything fearers) with distinct liking and emotional profiles; food explorers were most receptive to alternatives, while providing environmental information increased selection rates for alternative burgers without altering expected liking.
100	Ranga et al. (2023/2024)	N=183 students (France n=103, Ireland n=80), France and Ireland	Convergent parallel explanatory mixed-methods survey (open and closed-ended questions); Non-parametric tests (Mann-Whitney U, Chi-square, Kruskal-Wallis H, Spearman's correlation), thematic content analysis; Food Neophobia Scale, Food Disgust Scale, Food Choice Questionnaire	Edible insects and insect-containing foods / products under various conditions and visibility forms	Sociodemographics, country of study, previous knowledge/experience, food neophobia, food disgust, food choice motives, product characteristics (taste, visibility, nutrition, packaging, availability, brand)	Willingness to consume insects for the first time, after knowing regulatory framework, and based on product characteristics	Yes - Entomophagy is explored as a sustainable source of protein to mitigate the ecological footprint and environmental impacts (greenhouse gases, ammonia) associated with livestock production.	Students in France had a more positive opinion of entomophagy than in Ireland, and knowledge of the EU regulatory framework significantly increased willingness to consume (especially in France); food neophobia and disgust were major barriers, while taste and disguise (invisible insect ingredients) greatly improved willingness to consume over whole insects.
101	Siegrist & Hartmann (2020)	N=6128, Cross-national (Australia, China, England, France, Germany, Mexico, South Africa, Spain, Sweden, US), Consumers	Internet quota survey; One-way ANOVA, Tukey B post-hoc tests, Mediation analysis (Model 6 by Hayes)	Cultured meat	Stated willingness to eat cultured meat, perceived naturalness, disgust evoked by cultured meat, trust in food industry stakeholders, food neophobia, food disgust sensitivity (FDS-short)	Acceptance of cultured meat	Yes - Cultured meat is framed as an alternative protein source produced in a more environmentally friendly and animal-friendly way compared to conventional livestock farming.	Large cultural differences were observed, with French consumers showing significantly lower acceptance, naturalness, and higher disgust than all other countries; perceived naturalness and evoked disgust were crucial direct and indirect predictors of acceptance across all countries; trust, food neophobia, and food disgust sensitivity directly and indirectly influenced cultured meat acceptance in almost

								all countries.
102	Juraimi et al. (2025)	N=404 regular PBMA consumers (Singapore n=228, UK n=176), Singapore and the UK	Mixed-methods (quantitative online survey & inductive content analysis of open-ended questions); Chi-square tests, t-tests, Exploratory Factor Analysis (EFA), multiple linear regressions	Plant-based meat alternatives (PBMA)	PBMA consumption and purchase frequency, motivations and barriers to sustained consumption, product attribute importance, Food Choice Questionnaire (FCQ), Food Neophobia Scale (FNS), Meat Attachment Questionnaire (MAQ), needs/satisfaction qualitative content analysis	Overall PBMA consumption frequency	Yes - Plant-based meat alternatives are framed as alternative proteins necessary to mitigate greenhouse gas emissions and environmental challenges associated with traditional animal protein production.	PBMA consumption was similar between SG and UK, but motives, barriers, and consumer characteristics differed significantly; UK consumers were more sustainability-driven, while Singapore consumers valued health and faced more accessibility barriers; product-related motives and accessibility barriers significantly predicted overall PBMA consumption; qualitative results showed satisfaction with current options but a need for higher nutritional quality, affordability, and greater product variety.
103	Dzvukamanja et al. (2025)	N=537 (adolescents and young adults), Zimbabwe	Online cross-sectional survey; Pearson correlation, chi-square, binary logistic regression, multiple linear regression	Novel foods / edible insects and insect-based food (mopane worm porridge)	Demographics, standard Food Neophobia Scale (FNS), willingness to eat questionnaire	Prevalence of food neophobia, willingness to consume insects and insect-based foods	Yes - Mentions food system changes and the need to promote underutilized or climate-resistant species and novel foods to combat food insecurity and climate change effects.	Prevalence of food neophobia was low (14.4%), and willingness to consume insects was fair (39.5%); religion showed a minor association, but socioeconomic and demographic variables overall did not significantly predict food neophobia, implying urban youth are receptive to novel foods.
104	Cinar & Bozkiran Yilmaz (2025)	N=386, Türkiye (Izmir)	Face-to-face survey; Critic method, fuzzy paired comparison method, conditional valuation method, lower-bound mean method, multidimensional scaling, multiple correspondence analysis	Cultured beef (artificial meat)	Sensory factors in traditional beef consumption, reasons for not consuming cultured meat, food neophobia scale, willingness to pay (WTP)	Willingness to consume cultured beef, willingness to pay (WTP)	Yes - Cultured meat is framed as a sustainable alternative food product addressing greenhouse gas emissions, freshwater depletion, land use, and animal welfare concerns.	A significant portion (91.71%) were initially reluctant due to food neophobia, health concerns, taste expectations, and disgust; however, willingness changed positively when presented with social recommendations, market availability, and lower prices; consumers were willing to pay ~36% less than conventional beef (USD $13.716/kg), and health concerns/taste were the primary barriers.
105	Lu et al. (2026)	N=1021, USA (National survey)	Online survey; Structural equation modeling (SEM) via Confirmatory Factor Analysis (CFA)	Upcycled foods (products using ingredients that would otherwise be discarded)	Value-Attitude-Behavior (VAB) framework: economic value (price consciousness), quality concern, environmental value (food waste awareness), health value (health consciousness), food neophobia, attitude, consumption intentions	Attitudes and consumption intentions toward upcycled foods	Yes - Upcycled foods are framed as central to reducing food waste, advancing global sustainability efforts, and improving resource efficiency within a circular economy.	Food waste awareness and food neophobia were the most consistent predictors of attitudes and intentions; price and health consciousness influenced attitudes but not direct intentions, while quality concerns negatively affected consumption intentions; attitude strongly predicted consumption intention, supporting the VAB hierarchy.
106	Gere et al. (2017)	N=400 meat consumers, Hungary	Web-based survey; Zero-inflated Poisson (ZIP) regression, Multiple correspondence analysis (MCA)	Edible insects / insect-based food ingredients vs. other protein sources (soy, algae, whey)	Familiarity, food neophobia scale (Fneo), food technology neophobia scale (FTneo), health characteristics, environmental impact, meat nutrition belief, intention to reduce meat consumption	Readiness to adopt insects as a meat substitute, number of preferred insect types willing to try (Count)	Yes - Insects are highlighted as alternative protein sources with advantages in feed conversion efficiency, greenhouse gas/ammonia emissions, water use, and animal welfare.	Hungarian consumers exhibited higher food neophobia and lower familiarity with entomophagy than Western samples; food neophobia acted as a significant barrier, while familiarity and meat reduction intentions positively predicted readiness; males were more ready to eat insects than females.
107	Franco Lucas et al. (2025)	N=112, Switzerland (University restaurants in Bern and Zollikofen)	Real-life setting field study & online survey; Confirmatory Factor Analysis (CFA), Hayes' PROCESS macro for multivariate/mediation analysis	Insect-based burger (containing 24% minced mealworms, Tenebrio molitor)	Food neophobia, general health interest, environmental protection, natural content, attitudes toward recognizable vs. unrecognisable insect forms, insect phobia, insect as feed, disgust, real-life lunch choice	Choice of insect-based burger for lunch	Yes - Insect production is highlighted as a sustainable alternative requiring less feed and land than conventional meat to address environmental burdens.	62% of participants chose the insect burger when offered on the real-life lunch menu; disgust, insect as feed, natural content, and attitudes toward unrecognisable insect forms significantly predicted choice; disgust acted as a key direct barrier and a significant mediator for insect phobia and attitudes toward insect forms, showing that unrecognisable forms (e.g., ground mealworms in a burger) help reduce disgust and boost acceptance.
108	Sogari et al. (2019)	N=88 participants (students and faculty), Italy	Tasting experiment & questionnaire; Principal Components Analysis (PCA), Confirmatory Factor Analysis, Structural Equation Modeling (SEM)	House cricket incorporated into jelly sweets (unprocessed whole cricket vs. processed cricket flour jelly sweets)	Sociodemographics, past exposure (previous insect consumption), food neophobia scale (FNS), sensory property expectations (appearance, taste), willingness to try (WTT)	Intention to eat insects (WTT) and actual eating behavior (tasting behavior)	Yes - Entomophagy is supported by potential sustainable benefits such as a lower environmental impact than conventional animal protein sources.	Males were more open to trying insects than females; food neophobia negatively correlated with willingness to eat; past exposure positively increased sensory expectations and reduced neophobia; intention strongly predicted actual eating behavior (75% ate both products, 19% ate only processed, 6% ate neither); participants preferred processed/disguised insect products.
109	Appiani et al. (2025)	N=202 total (N=104 adults, N=98 children aged 9–11), Italy (Milan)	Sensory evaluation tasting session; GLM ANOVA, Check-All-That-Apply (CATA) method, Correspondence Analysis (CA), penalty-lift	5 plant-based breaded fish analogues (wheat and rice-based) and 2 animal-based control samples (cod fillet and stick)	Overall liking, sensory properties (CATA), emotional responses, food neophobia, family/child fish and plant-based	Overall liking / consumer acceptance, sensory drivers, emotional profiles	Yes - Framed in the context of sustainability transitions, environmental impacts of animal-based food production, and the need to develop sustainable food	Animal-based samples scored higher in liking, but plant-based analogues were well accepted by both age groups; adults preferred the wheat protein-based fillet, while children preferred the rice flake-based stick; off-flavours, legume

			analysis, Multiple Factor Analysis (MFA), Food Neophobia Scale (FNS/ICFNS), EmoSemio & Emoji Pair Rating Scale		consumption habits		alternatives.	odours, bitterness, and pasty/gummy textures negatively impacted liking; positive emotions correlated with animal products and liking, while wheat protein evoked curiosity/surprise in adults; food neophobia showed no significant effect due to low neophobia levels in the sample.
110	Rao et al. (2025)	N=592 meat-eating adults (N=325 Australia, N=267 Denmark), aged 20–70	Online questionnaire; Welch's t-tests, Chi-squared tests, Pearson's correlations, mediation analysis using PROCESS macro with age and sex covariates	Plant-based meals and dietary patterns	Familiarity with plant-based meals, Food Neophobia Scale (FNS), Cognitive Reflection Test (CRT), Meat-centric meal perception (8-item scale), frequency of plant-based meal consumption	Frequency of plant-based meal consumption, meat-centric meal perception (as a mediator)	Yes - Framed around sustainable dietary transitions to address the climate crisis, conserve planetary resources, improve public health, and reduce greenhouse gas emissions and environmental burdens associated with meat consumption.	A stronger meat-centric meal perception was significantly correlated with consuming fewer plant-based meals in both Australia ($r=-0.36$) and Denmark ($r=-0.42$); higher familiarity with plant-based meals indirectly increased plant-based consumption by reducing meat-centric meal perception in both countries; food neophobia directly and indirectly predicted lower plant-based consumption; cognitive reflection showed no significant association.
111	Rombach et al. (2025)	N=1171 total across two periods (571 in 2017, 600 in 2022), China	Online survey administered in 2017 and 2022; Partial Least Squares Structural Equation Modeling (PLS-SEM), Measurement Invariance of Composite (MICOM), Multi-group Analysis (MGA)	Cultured meat, insect-based protein, and plant-based protein	Health importance (food healthiness), environmental concerns, food neophobia, food technology neophobia, emotional responses, rational assessments	Willingness to consume cultured muscle, insect-based protein, and plant-based protein (WTC)	Yes - Climate change, growing population, and demands for more sustainable food production/consumption drive alternative protein choices.	Willingness to consume alternative proteins was driven by rational assessments and emotional responses while hindered by food neophobia across both periods; food healthiness significantly influenced plant protein in 2017 and insect protein in 2022; food technology neophobia significantly hindered cultured muscle and plant protein consumption only in 2022; multi-group analysis showed significant decreases in the influence of emotional response on insect protein and rational assessment on cultured muscle from 2017 to 2022.
112	Onat et al. (2026)	N=400 undergraduate gastronomy and culinary arts students across all 7 regions of Turkey	Online survey, Confirmatory Factor Analysis (CFA), Structural Equation Modeling (SEM), bias-corrected bootstrap mediation analysis, Heterotrait-Monotrait (HTMT) discriminant validity	Edible insects (general food ingredient category)	Perceived future food insecurity (9-item scale), food neophobia scale (10-item FNS), food interest scale (3 items), intention to incorporate edible insects into recipes (12-item adapted scale)	Intention to incorporate edible insects into recipes (recipe incorporation intention)	Yes - Framed around sustainable protein transformation, low environmental impact, high nutrient density, and suitability for circular food systems to address future resource scarcity and population growth.	Perceived future food insecurity had no significant direct effect on recipe incorporation intention; however, it indirectly influenced intention through two mediators: negative association with food neophobia and positive association with food interest; food interest strongly predicted recipe incorporation intention ($\beta=0.715$), while food neophobia negatively predicted it ($\beta=-0.225$); both pathways represented indirect-only mediation.
113	Szczepanski et al. (2022)	N=114 adolescents (grades 9–12, mean age 15.77 years), 3 secondary schools in Osnabrück, Germany	Questionnaire intervention study (pre-test, post-test, follow-up test design), 4-lesson teaching unit ("Entomophagy and Sustainability"), repeated measures ANOVA (rmANOVA), Bonferroni post-hoc tests, multiple hierarchical regressions	Insect-based food (burger patties, pasta, protein bars)	Sociodemographics, food disgust scale (short version), food neophobia scale (children's version), food technology neophobia scale, attitudes toward insect-based food, entomophagy knowledge test, willingness to consume index (try, buy, substitute meat)	Willingness to consume insect-based food, changes in nutritional-psychological factors, attitudes, and knowledge over time	Yes - Framed around global problems (climate change, loss of biodiversity, hunger), sustainable protein transition, and mitigating greenhouse gas emissions from industrial livestock farming.	The teaching unit significantly reduced food disgust, food neophobia, and food technology neophobia, and significantly improved attitudes and knowledge about entomophagy (with gains lasting into the follow-up test); however, students' willingness to consume insect-based food did not significantly increase over time; attitudes were the strongest predictor of willingness to consume across all measurement points, while knowledge did not directly predict willingness to consume.
114	Loera et al. (2026)	N=1500 total (N=500 per country: Italy, the Netherlands, France), quota-representative by gender, age, and education	Cross-national online survey (CAWI); ANOVA, Bonferroni post-hoc tests, Tukey's HSD, Pearson's correlations, simple/hierarchical multiple linear regressions	Cultured meat (CM) (evaluated via a CM burger scenario)	Sociodemographics, engagement/awareness with CM, psychological background factors (Natural-is-better heuristic, trust in food safety authorities/scientists, environmental concern, food neophobia, food technology neophobia), attitudes towards CM, willingness to try (WTT) and buy (WTB)	Behavioural intentions (WTT, WTB) and attitudes towards cultivated meat	Yes - Framed around the protein transition, planetary boundaries, mitigating conventional animal-source food production impacts, greenhouse gas emissions, and land use.	Italian consumers showed high engagement and openness to CM comparable to Dutch consumers, whereas French consumers (especially in western regions) were significantly more reluctant; attitudes strongly predicted intentions; food technology neophobia and distrust in food safety authorities emerged as the most consistent barriers across all countries, while the natural-is-better heuristic was non-significant.
115	Modlinska et al. (2021)	N=1096 nationwide representative random quota sample, Poland	Online panel survey via Computer-Assisted Web Interview (CAWI); descriptive statistics, Student's t-tests, ANOVA, Binary Logistic	Insect-based food / insects as a meat substitute and food with insect-derived ingredients	Acceptance of insects as a meat substitute (MS), acceptance of food with insect-derived ingredients (EA / Entomophagy Attitude	Acceptance of the idea of insects as a meat substitute (MS) and willingness to accept food with insect-derived ingredients (EA)	Yes - Framed around environmental and ethical issues linked to meat production and insects as a sustainable source of protein and essential nutrients.	There is a significant discrepancy between accepting the abstract idea of insects as a meat substitute and the actual willingness to buy/consume insect-based foods (the two were uncorrelated and predicted by different

			Regression, Multiple Regression		Questionnaire), familiarity, health attitudes (FHealth), convenience orientation (FConv), environmental attention (FEnv), food neophobia (FN), general neophobia (GN), disgust sensitivity (DS), variety-seeking tendency (VS), traveling frequency, sociodemographics			factors); acceptance of the general idea was predicted by familiarity, environmental attention, convenience, and male gender; actual willingness to consume insect-based food was predicted by lower food neophobia, lower disgust sensitivity, and higher variety-seeking tendency.
116	Mikulec et al. (2024)	N=1063 university students across multiple university profiles and provinces, Poland	Online platform (CAWI) indirect interview method (November 2023); Exploratory factor analysis, scale reliability (Cronbach's alpha), Spearman correlation analysis, Multiple Regression Analysis (MRA), Path analysis / structural equation modeling	Food made from insects / insect-based food	Health attitudes (HA), environmental attitudes (EA), attitude towards novel food / food neophobia (NF), attributes of novel food (organoleptic traits CA1, health quality CA2, functional traits CA3), attitudes towards food made from insects (ATT), intention to purchase food made from insects (PI)	Attitudes towards food containing insects (ATT) and intentions to purchase food containing insects (PI)	Yes - Framed around sustainable development, environmental protection, reducing area/water consumption and greenhouse gas emissions, zero waste, and circular economy.	Health quality attributes (CA2) and organoleptic attributes (CA1) strongly influenced positive attitudes and purchase intentions, with health quality traits having a far greater impact than organoleptic or functional traits; food neophobia negatively correlated with attitude and purchase intention ($r=-0.30$ to -0.32); attitudes strongly predicted purchase intentions ($\beta=0.63$ to 0.64); structural path analysis confirmed a good fit explaining ~70% of variance.
117	Caparros Megido et al. (2016)	N=79 university students (aged 18–25 years, 51% women), Belgium	Questionnaire survey & hedonic tasting sessions; microbiological analysis of burger samples, 2-way ANOVA with general linear model (GLM), Kruskal-Wallis test, Cohen's d effect size	4 burger patties: beef burger (BB), lentil burger (LB), mealworm/beef burger (MBB, hybrid), and mealworm/lentil burger (MLB, hybrid) using mealworms (Tenebrio molitor)	Sociodemographics, preconceptions/awareness of entomophagy, previous insect consumption experience, 9-point hedonic scales (appearance, odor, taste, overall liking), post-tasting survey questions on future perception and willingness to eat	Sensory-liking (overall liking, appearance, odor, taste) and post-tasting perception/willingness to eat	Yes - Framed around sustainable protein sources, low environmental impact, low greenhouse gas emissions, high conversion rates, and recycling organic industrial/agricultural byproducts as an alternative to meat production.	Beef burger was best rated overall, but insect-based hybrid burgers (MBB, MLB) rated intermediately between beef and lentil burgers with higher-than-neutral scores; women preferred beef burger appearance while men preferred beef and insect burgers; previous insect consumption and entomophagy knowledge positively affected taste/liking evaluations; 84% improved their perception of entomophagy after tasting, and 68% were ready to add insects to their diet (mostly minced or powdered).
118	Aschemann-Witzel et al. (2022)	N=1603 consumers from 5 Northern and Southern European countries (UK, Denmark, Germany, Portugal, Italy)	Between-subjects online survey ($3\times2\times2$ design varying framing, region, and product category origin); manipulation check, Analysis of Variance (ANOVA) with main effects, 2-way interactions, and hypothesized interactions	Upcycled food assortment across categories (bakery, dairy, sweet/salty snacks) incorporating by-products (spent grain, oilcake, berry skin, whey, coffee ground, grape skin)	Attitude towards upcycled food, likelihood to buy (0–100%), environmental concern, frugality orientation, food neophobia, sociodemographics	Attitude towards upcycled food and likelihood to buy	Yes - Framed around United Nations Sustainable Development Goals (halving food waste), reducing global greenhouse gas emissions, resource efficiency, and circular economy.	Framing upcycled food around traditional frugality ("using all of what you have") resulted in significantly higher consumer attitudes compared to taste framing; environmental concern and food neophobia were strong psychographic drivers/barriers respectively; frugality orientation also positively predicted attitude; products typical for one's own region were rated more favourably; younger consumers and females showed more favourably attitudes.
119	Tan et al. (2016)	N=103 Dutch beef consumers (62 males, 41 females, mean age 22.9 years), The Netherlands	Tasting session and questionnaire; 2-way ANOVA (4 labels $\times$ 4 recipes) with covariates (gender, food neophobia), paired samples t-tests, Pearson's product-moment correlations, hierarchical linear regressions	4 beef-based burger patties combined with 4 plant-based recipes (Recipes A-D) and 4 label descriptions: 100% beef burger vs. novel burgers labelled as containing 75% beef and 25% novel ingredients (lamb brain, frog meat, mealworms)	Ingredient familiarity, expected and perceived sensory-liking (before/after tasting), food appropriateness (before/after tasting), future willingness to eat in different situations (meal, special occasion, snack) and forms (burger vs. ingredient), Food Neophobia Scale (FNS), gender	Sensory-liking, food appropriateness, and future willingness to eat	Yes - Mentions insects and sustainable production as more sustainable than traditional livestock, though focusing primarily on psychological and sensory/appropriateness barriers.	Before tasting, expected sensory-liking and food appropriateness of novel burgers were lower than the beef burger; after tasting, experienced sensory-liking of novel burgers increased to the same level as beef, but food appropriateness remained lower; future willingness to eat was predicted primarily by food appropriateness rather than experienced sensory-liking or food neophobia; positive sensory experience is necessary but inadequate for unusual/culturally inappropriate foods.
120	van Dijk et al. (2023)	N=376 omnivore and flexitarian participants from the Netherlands ($n=126$) and Finland ($n=250$)	Online survey; two-way ANOVAs, linear regressions	Plant-based meat substitutes, cultured meat, and hybrid meat products	Demographics, consumption frequency, Food Sustainability Knowledge Questionnaire (FSKQ), Food Neophobia Scale (FNS), Meat Attachment Questionnaire (MAQ), familiarity, attitudes, willingness to buy (WTB)	Attitudes and willingness to buy meat substitutes, cultured meat, and hybrid meat products	Yes - Framed around the climate crisis, global greenhouse gas emissions, food system contributions, and livestock farming impacts.	Meat substitutes and hybrid meat products scored significantly higher in overall attitude than cultured meat, though WTB for hybrid meat and cultured meat was lower than for meat substitutes; flexitarians represent an important target group; food neophobia and meat attachment negatively predict WTB, while familiarity and sustainability knowledge positively predict it.
121	Sick et al. (2024)	N=119 Danish children aged 11–13 years (from 9	Sensory evaluation during science outreach week; 15-point hedonic scale, Check-All-	Six whole roasted or deep-fried insects (buffalo worms, crickets,	Demographics, familiarity, willingness to try insects, Food Neophobia Test Tool	Hedonic ratings (liking) and sensory profiles of insect types	Yes - Framed around sustainable diets, high-quality proteins for human consumption, and reducing	Mealworms, buffalo worms, and bee larvae received higher mean liking than crickets, wax moth larvae, and grasshoppers; "crunchy" was

		primary schools in greater Copenhagen area)	That-Apply (CATA) with 50 sensory attributes, Correspondence Analysis (CA), penalty analysis, Food Neophobia Test Tool (FNTT), univariate ANOVA	grasshoppers, mealworms, wax moth larvae, bee larvae)	(FNTT), hedonic ratings (liking), CATA sensory descriptions		traditional protein sources like meat.	significantly associated with liking; food neophobia negatively affected hedonic acceptance; younger children (11 y.o.) and boys gave higher hedonic ratings.
122	Wang et al. (2026)	Three experiments in China: Study 1 ($n=300$), Study 2 ($n=397$), Study 3 ($n=400$)	Experimental design across 3 studies; ANCOVAs, parallel mediation analyses (PROCESS Model 4, 7, 14, 58, 59)	Insect-based foods (chocolate nut balls with mealworm protein, protein oatmeal, ice cream corn)	Influencer type (health-focused vs. indulgence-focused), food category (utilitarian vs. hedonic), perceived healthiness, perceived tastiness, consumer acceptance, health consciousness, food neophobia, disgust toward entomophagy, prior entomophagy experience	Consumer acceptance, perceived healthiness, perceived tastiness	Yes - Framed around population growth, resource scarcity, climate change, and sustainable protein sources (edible insects).	Health-focused influencers consistently enhanced perceived healthiness of insect-based foods, which in turn increased consumer acceptance; perceived tastiness did not differ by influencer type and did not mediate the overall effect, though tastiness was a stronger predictor of acceptance; the positive indirect effect via healthiness was more pronounced for hedonic foods and significant only among health-conscious consumers with no prior insect consumption experience; food neophobia and disgust did not moderate these pathways.
123	Quevedo-Silva & Pereira (2022)	N=304 Brazilian consumers (online nationwide survey)	Quantitative descriptive survey; Partial Least Squares Structural Equation Modeling (PLS-SEM), convergent/discriminant validity (Fornell-Larcker), bootstrapping	Cultured (cultured) meat	Clean production, animal welfare, food safety, unnaturalness, food neophobia (moderator), purchase intention	Cultured meat purchase intention	Yes - Framed around greenhouse gas (GHG) emissions from ruminant breeding, climate change, and environmental impacts of conventional livestock production.	Clean production and food safety had a positive relationship with cultivated meat purchase intention, whereas unnaturalness had a negative relationship; animal welfare was not significant; food neophobia moderated the relationship between clean production and purchase intention (higher neophobia decreased the importance of clean production).
124	Pronk et al. (2025)	N=1953 meat-eating participants across Germany ($n=472$), Finland ($n=495$), Italy ($n=498$), and Serbia ($n=488$)	Online survey; repeated-measures ANOVAs, logistic regressions	14 different protein sources for meat alternatives (plant-based proteins like potato, rice, peas, soy, oats, lentils, almond, faba beans, rapeseed, sunflower, plus algae, insects/crickets, and cultured beef)	Expected taste, expected healthiness, expected environmental friendliness (0–100 continuous sliders), food consumption frequency, Food Neophobia Scale (FNS), meat commitment scale, meat alternative rejection scale	Expected taste/healthiness/environmental friendliness ratings for alternative protein sources, consumption frequency of traditional and new meat-like meat alternatives	Yes - Framed around the climate crisis, global greenhouse gas emissions, and sustainable dietary shifts away from conventional meat.	Potato, rice, and peas were identified as the most accepted protein sources across countries; algae and cultured meat had low expectations, and insects (crickets) were the least accepted; country-specific preferences emerged (e.g., oats in Finland, almonds in Germany/Serbia, lentils in Italy); food neophobia and meat alternative rejection predicted lower consumption, while familiarity and lower meat commitment increased consumption.
125	Elorinne et al. (2019)	N=567 convenience sample in Finland (74% omnivores, 22% non-vegan vegetarians, 5% vegans)	Online survey; Cronbach's alpha reliability analysis, Chi-Square tests, One-Way ANOVA, K-means cluster analysis	Foods of insect origin / insect-based foods	Attitude (A), subjective norm (SN), perceived behavioral control (PC), intention (I) toward insect consumption based on Theory of Planned Behavior (TPB), modified Food Neophobia Scale (FN), perceived behavioral conditions (healthiness, safety, convenience, price)	Attitude, subjective norm, perceived behavioral control, intention to eat foods of insect origin, consumer clusters (likely, potential, unlikely consumers)	Yes - Framed around the environmental impact of meat production and insects as a sustainable alternative protein supplement.	Dietary groups exhibited significant differences; vegans held rigid negative attitudes, viewed insect consumption as immoral/irresponsible, had lower subjective norms and higher food neophobia/behavioral control, and mostly grouped as "unlikely consumers" (69.2%); non-vegan vegetarians and omnivores held more positive attitudes, viewed insects as a solution to world nutrition problems, and mostly grouped as "likely consumers" (56.4%–58.1%).
126	Mota-Gutierrez et al. (2026)	N=222 Italian schoolchildren aged 8–10 years (from 7 primary schools in Turin, Italy)	Within-subjects design with repeated measures, paper-based questionnaires across two sessions with educational lectures, Cumulative Link Mixed Models (CLMM), Latent Class Analysis (LCA)	Whole insects (buffalo worms, crickets) and insect-based snacks (buffalo worm and cricket protein bars, buffalo worm and cricket chocolates)	Willingness to eat (WTE) insect-based snacks, Food Neophobia Test Tool (FNTT), Food Disgust Scale (FDS), entomophagy awareness, educational information intervention, eating behavior profiles (Latent Class Analysis clusters: cautious eaters vs. adventurous tasters)	Willingness to eat insect-based snacks, acceptance of whole insects and insect-based foods, appropriateness as food ingredients	Yes - Framed around sustainable food systems, alternative protein sources, population growth, and resource pressures.	Educational information increased willingness to eat and acceptance of insect-based foods and attenuated the negative influence of neophobia for cricket protein bars; lower food neophobia and lower food disgust were associated with higher acceptance; latent class analysis identified two profiles ("cautious eaters" vs. "adventurous tasters"), with adventurous tasters showing significantly higher openness to insect-based snacks.
127	Birrell et al. (2026)	N=620 participants in New Zealand (online questionnaire)	Mixed-methods approach combining quantitative survey data with term frequency-inverse document frequency (TF-IDF), sentiment analysis, logistic regression via tidymodels	Word association menu-reading scenario across 14 ingredients spanning animal, plant, and insect groups (locust/sky prawn, mealworm, grasshopper, huhu grub, cricket flour, silkworm pupae)	Prior experience, food neophobia score, existing knowledge (listing edible insect species), demographic variables, word associations and sentiment scores (+1, 0, -1), TF-IDF term frequency	Future likelihood of eating an insect (likely/unlikely), sentiment scores of word associations	Yes - Framed around sustainable protein development.	Prior experience, existing knowledge, and low food neophobia were significant predictors of future willingness to consume insects (logistic regression $R^2=0.23$); sentiment analysis revealed that insect ingredients received significantly lower sentiment scores than animal or plant ingredients, except for cricket flour, which elicited favorable, food-anchored associations; traditional/indigenous species like huhu grubs had neutral/positive cultural

								associations, while pests/silkworms/mealworms evoked negative text associations.
128	Simeone & Scarpato (2022)	N=117 completed questionnaires from respondents responsible for food purchases, Rome, Italy	Online questionnaire survey, probit model analysis, descriptive statistics, Student's t-tests, pairwise correlation indices	Insect-based foods / edible insects (four product images across visibility levels: whole/visible, whole/coated, ground/invisible, processed/protein bars)	Sociodemographics, food neophobia levels (FNS, informed vs. non-informed conditions), entomophagy knowledge, 9 food values (healthiness, naturalness, taste, price, safety, convenience, appearance, origin, environmental impact)	Knowledge of sustainable diets, willingness to consume insect-based food, hedonic liking and evoked emotions for insect images	Yes - Framed around food security, sustainability, reducing greenhouse gas emissions, water use, and land requirements as a sustainable meat substitute.	Consumers aware of sustainable diets were not necessarily worried about high environmental impacts because they already account for them; social acceptance of insect consumption is very low even among those sensitive to sustainable diets; appearance and disgust are major barriers (visible insects evoke disgust, while processed/invisible products increase curiosity and appetite); healthiness, naturalness, and environmental impact are recognized as positive food values.
129	Van der Stricht et al. (2024)	N=3027 respondents across five European countries (the Netherlands, Germany, Hungary, Spain, Italy)	Quantitative online survey, binary logistic regression, exploratory factor analysis, two-stage segmentation analysis (hierarchical and K-means), multinomial logistic regression	Foods with microalgae proteins (Spirulina / microalgae protein ingredients)	Willingness to try (WTT), attribute perceptions (credence vs. experience dimensions), General Health Interest (GHI), Food Neophobia Scale (FNS), Food Technology Neophobia Scale (FTNS), Environmental Concern (EC), familiarity, dietary status, information cue interest, sociodemographics	Willingness to try foods with microalgae proteins, consumer market segments ("Enthusiast", "Cautiously curious", "Undecided", "Uninterested")	Yes - Framed around sustainable protein sources, meeting nutritional needs of a growing population, reducing greenhouse gas emissions, deforestation, and land/water requirements.	About 51.9% were unaware of microalgae foods; binary logistic regression showed flexitarian diet, GHI, FNS, FTNS, and EC significantly shape willingness to try; segmentation identified four clusters ("Enthusiast" 27.7%, "Cautiously curious" 29.5%, "Undecided" 29.8%, "Uninterested" 13.0%); health interest, environmental concern, and low neophobia strongly increased the likelihood of belonging to receptive segments.
130	Ordoñez López et al. (2023)	N=617 respondents in France	Online survey (46 questions), Wilcoxon/Kruskal-Wallis tests, chi-square tests, bivariate correlations, Multiple Correspondence Analysis (MCA)	Insect-based food (IBF) / edible insects	Sociodemographics, traveling/living abroad experiences, Food Neophobia Scale (Pliner & Hobden, 1992), Food Disgust Scale, food choice influencers, entomophagy familiarity, exposure sources, known benefits, eating occasions, experience ratings, motivators, barriers, COVID-19 impact	Willingness to consume insect-based food regularly, entomophagy acceptance	Yes - Framed around global food security, sustainability, population growth, greenhouse gas emissions, and sustainable alternative protein sources.	69% had been introduced to entomophagy, 53% had consumed IBF before; average willingness score was 2.83/5 (slightly unwilling); environmental sustainability and nutritional value were main motivators, while unfamiliarity, limited availability, and disgust were main barriers; younger adults (18–30) and frequent travelers showed higher willingness and lower neophobia; repeated exposure and restaurant experiences improved willingness and reduced disgust/neophobia.
131	Rabitti et al. (2021)	N=127 consumers (63 women, 64 men, aged 19–60 years), Italy	Sensory laboratory test across two sessions (1 week apart), blind, expected, and real hedonic rating (10 cm linear scales), paired t-tests, Food Neophobia Scale (FNS), sustainability attitude questionnaires	Whole corn-based gluten-free formulations ("polenta") as control (100% whole corn) and two enriched samples replacing 20% of whole-corn flour with chickpea or red lentil flour	Blind liking, expected liking (based on health/environmental information and product images), real liking (tasting with information), Food Neophobia Index (FNS categorized into neophilics, neutrals, neophobics), Sustainability Index (SUS index based on sustainable behaviors, environmental impact knowledge, beliefs/attitudes towards sustainable foods)	Hedonic liking scores under blind, expected, and real conditions	Yes - Framed around agri-food system environmental impact, climate crisis, agricultural intensification, water scarcity, and promoting sustainable pulse-based formulations.	No significant differences in baseline liking among the three formulations (all accepted above scale midpoint); information increased hedonic expectations, resulting in an incomplete assimilation effect for pulse samples; neophilic and neutral consumers showed positive responses/assimilation to information, whereas neophobic consumers were completely insensitive to health/environmental information; food neophobia was negatively correlated with sustainability commitment (SUS index).
132	Arena et al. (2020)	N=210 completed questionnaires (from 255 online survey respondents), Southern Italy	Online survey using probit model, ANOVA, Student's t-tests, pairwise correlation index values	Insect-based foods / edible insects presented via 4 images at different visibility levels: F1 (whole/visible mix of insects), F2 (whole/coated chocolate-coated grasshopper and scorpions), F3 (ground/invisible crickets and silkworm flour), F4 (processed/invisible protein bars)	Sociodemographics, Food Neophobia Scale (FNS) (subdivided into informed and non-informed subgroups), hedonic liking (9-point scale), evoked emotions (curiosity, disgust, appetite), 9 food values (healthiness, naturalness, taste, price, safety, convenience, appearance, origin, environmental impact)	Knowledge of sustainable diets, willingness to try insect-based foods, hedonic liking and emotions for insect images	Yes - Framed around food security, sustainability, high-quality proteins respecting animal welfare and the planet, and environmental impact.	Information about edible insects increased food neophobia among high neophobia respondents and negatively affected evaluations of appearance and origin; expected liking was low for visible insects (F1/F2) which evoked strong disgust, but increased when insects were invisible in processed products (F4) which evoked curiosity; consumers recognized healthiness, naturalness, and environmental impact as positive food values in insect foods, but overall social acceptance in Southern Italy remains very low.
133	Vartiainen et al. (2020)	N=567 respondents (convenience sample, 66.8%	Online questionnaire survey, convenience sampling, Mann-Whitney U test, Kruskal-Wallis	Insect-based foods / insects as food	Demographics, earlier experience, Food Neophobia Scale (FN), Theory of	Intention to consume insect-based foods (I), consumer clusters ('likely consumers',	Yes - Framed around greenhouse gas emissions, land requirements, water use, feed conversion rate of insect	70% intended to consume insect-based foods; attitude, subjective norm, perceived behavioral control, and gender explained 80% of variance

		women, 33.2% men), Finland	H-test, hierarchical regression analysis, K-means cluster analysis, Food Neophobia Scale		Planned Behaviour constructs (Attitude [A], Subjective Norm [SN], Perceived Behavioral Control [PBC]), perceived conditions (Healthiness [H], Safety [S], Convenience and price [C])	'potential consumers', 'unlikely consumers')	rearing compared to cattle, and global food security.	in intention; food neophobia negatively correlated with intention ($\rho=-0.501$); women, rural residents, under 25s, and those with no prior experience had less intention; three consumer clusters identified ('likely consumers' 44%, 'potential consumers' 39%, 'unlikely consumers' 17%).
134	Tian & Chen (2025)	N=560 Chinese university students (aged 18–25 years), Xinyang Normal University, China	Online questionnaire survey via Wenjuanxing, Mann-Whitney U tests	Insect-based foods / edible insects	Familiarity, consumption frequency of specific insect foods, reasons for not eating, Food Neophobia Scale (FNS), Food Disgust Scale (FDS), willingness to consume, perceived benefits (nutrition, sources, cost, environmental protection, cooking, health), perceived risks (microorganisms/diseases, food fear/disgust, poison/toxin, sensory dislike, insecticides/chemicals, allergy, general health risks)	Willingness to consume insect foods, perceived benefits and risks	Yes - Framed around global population growth, resource diminishing, biological resource shortages, environmental pollution, and sustainable development / alternative protein resources.	Most university students were unfamiliar with (70%) and unwilling to consume insect foods (66.79% never eaten); higher levels of food neophobia and food disgust indicated greater unwillingness; regardless of neophobia or disgust levels, both high and low groups agreed on the benefits of insect food but perceived high risks; unfamiliarity and disgust were the primary barriers.
135	Ros-Baró et al. (2022)	N=1034 adult consumers (mostly Catalonia/Spain)	Observational descriptive cross-sectional ad-hoc questionnaire survey via Qualtrics, descriptive statistics, Pearson's chi-square tests	Edible insects / insect-based food (various formats: flours, bars, biscuits, whole insects, etc.)	Sociodemographics, food neophobia, social norms, familiarity, consumption experiences, knowledge of benefits, willingness to consume/cook/offer in restaurants, preferred presentations/preparations, reasons for refusal	Willingness to consume, cook, or include insects in the usual diet	Yes - Framed around sustainable protein alternatives, environmental pressures on agriculture/farming, greenhouse gas emissions, land/water use, and sustainable development goals.	Only 13.15% had tried insects; disgust (38.2%), lack of custom (15.37%), and safety concerns were main barriers; flour-based preparations were the most preferred/attractive format (23.5%); 40–59 age group and males showed higher willingness; knowing that insect consumption has sustainable potential improved willingness to consume (56.17% would do so for sustainability reasons).
136	Tuccillo et al. (2026)	N=980 individuals from Finland ($n=530$), Ireland ($n=196$), and the Netherlands ($n=254$)	Online questionnaire survey, exploratory factor analysis, partial least squares structural equation modeling (PLS-SEM), mixed-design ANOVA, hierarchical cluster analysis (6 segments), multiple factor analysis (MFA)	Three visually presented jellyfish preparations: jellyfish arms served as salad (Set A), jellyfish umbrella strips served as soup (Set B), and whole umbrella prepared as chips (Set C)	Sociodemographics, dietary status, importance/frequency of low-calorie foods, Travel-related Food Behavior (TRFB), single-item Food Choice Questionnaire (FCQ), Food Neophobia scale (FN), Sensitivity to Disgust scale (DS), Jellyfish Consumption Acceptance (JCA) scale, perceived appropriateness, expected liking, willingness to buy (WTB), preferred consumption formats, contexts, food pairings, evoked emotions (13 emotions), and stimulus characteristics	Jellyfish consumption acceptance, perceived appropriateness, expected liking, willingness to buy, emotional responses, consumer segments	Yes - Framed around marine resource sustainability, ecological impacts/jellyfish blooms, reducing environmental impact, and diversifying fisheries.	Cross-country differences in attitudes, consumption preferences, and emotional responses (Finland showed higher acceptance/positive emotions than Ireland and the Netherlands); higher willingness to consume jellyfish when presented in processed/culinary-integrated forms, paired with fish and rice, and in exploratory dining contexts; stimuli clustered by country rather than preparation type; six consumer segments identified (angry rejectors, disgusted rejectors, neutrals, calm acceptors, excited acceptors, proud acceptors) linked to sociodemographics, diet, JCA, neophobia, disgust sensitivity, and travel food behavior.
137	Erhard et al. (2023)	N=181 Danish children aged 9–13 years (mean 11.8) across 4 schools and 9 classes	Online learning activity, lecture/quiz intervention (taste, health, or environmental benefits), pre- and post-exposure questionnaire, Linear Mixed Models (LMMs), paired t-tests, Food Neophobia Test Tool (FNTT), Food Disgust Scale (FDS-short)	Unprocessed insects (buffalo worms and crickets - dried/frozen) and nine commercially available insect-based products made with buffalo worm or cricket flour (protein bars, crispbreads, chocolate, burger, falafel, cookies, chips)	Demographics, familiarity, willingness to try (WTT) before and after intervention, Food Neophobia Test Tool (FNTT), Food Disgust Scale (FDS), hedonic response (7-point emoji scale) to whole insects and products, perceived appropriateness as a food ingredient vs. livestock	Willingness to try (WTT) insect foods, hedonic responses to insect species and insect-based products, perceived appropriateness	Yes - Framed around growing global population, rising demand for meat, resource pressure on land/water/energy, and edible insects as a promising alternative protein source.	Communicating information about benefits (taste, health, sustainability) did not increase willingness to try insect foods; food neophobia was a strong negative predictor of willingness to try, whereas food disgust sensitivity had no effect and did not correlate with neophobia; buffalo worms were rated significantly higher in hedonic response and appropriateness than crickets when whole, but species differences disappeared when incorporated into processed flour products (e.g., bars, bread); cookies were the best-liked product.
138	Osorio-Andrade et al. (2025)	N=489 supermarket customer participants in the Valle del Cauca region of Colombia	Survey questionnaire, Partial Least Squares Structural Equation Modeling (PLS-SEM), convergent/discriminant validity testing (Fornell-Larcker, HTMT, cross-loadings)	Insect-based foods / edible insects	Consumer age, educational level, economic power (socioeconomic stratum), food neophobia, preference for traditional protein, attitude towards insects, perceived benefits, environmental awareness,	Adoption of insect-based foods (intention to consume)	Yes - Framed around food sustainability, alternative protein sources, lower carbon footprint, and efficient resource use compared to traditional livestock.	Attitude toward insects ($\beta=0.100$) and perceived benefits ($\beta=0.176$) were positively correlated with consumption intentions; food neophobia ($\beta=-0.201$), food-related health concerns ($\beta=-0.094$), and older age ($\beta=-0.291$) negatively affected acceptance; environmental awareness, educational level, and socioeconomic stratum

					food-related health concerns, perceived importance of traditional nutrition, political ideology, adoption of insect-based foods			did not exhibit significant relationships; psychological and perceptual factors are the primary drivers of edible insect acceptance in Colombia.
139	Bae & Choi (2021)	N=392 adult citizens of Korea (Gyongsang-do and Chungcheong-do areas)	Survey questionnaire, independent t-tests, multiple regression analysis, descriptive statistics	Edible insect foods (EIFs) including silkworm pupa, rice grasshopper, Chinese silkworm, 2-spotted cricket, mealworm, maggot, rhino beetle (and product concepts like insect shape vs. without insect shape)	Food neophobia (FN), subjective norms (SN), perceived behavioral control (PBC), attitude (ATT), experience with EIFs (EX), behavioral intention (BI), demographic characteristics	Behavioral intention to purchase/consume EIFs	Yes (framed around potential sources of nutrition/protein for the future and efficiency compared to meat products).	About 63.5% had previously eaten EIFs (mostly silkworm pupa); primary reason for not eating EIFs was disgust caused by insect shape (51.7%); independent t-tests showed significant differences between experienced and inexperienced groups in food neophobia, subjective norm, attitude, and behavioral intention, and between high and low neophobia groups in SN, PBC, ATT, and BI; multiple regression showed that subjective norm and attitude significantly influenced behavioral intention ($R^2=0.549$), while experience, neophobia, and PBC did not directly affect intention.
140	Rehman et al. (2023)	N=497 consumers in Pakistan (Islamabad, Lahore, and Gujranwala)	Convenience sampling survey questionnaire, Partial Least Squares Structural Equation Modeling (PLS-SEM) using SmartPLS, Harman's single factor test, multiple regression analysis	Organic food products	Environmental concerns (EC), knowledge of organic food (KOF), food neophobia (FNB), food neophilia (FNF), health consciousness (HC), social norms (SN), satisfaction toward organic food (SOF), perceived barriers (PB), intention to purchase organic food (IPOF)	Satisfaction toward organic food, intention to purchase organic food (IPOF)	Yes - Framed around organic food protecting the environment, avoiding chemicals/pesticides, and increased demand during the COVID-19 pandemic.	Environmental concerns ($\beta=0.188$), knowledge of organic food ($\beta=0.091$), food neophilia ($\beta=0.325$), health consciousness ($\beta=0.158$), and social norms ($\beta=0.284$) are positively related to satisfaction toward organic food; food neophobia decreases satisfaction toward organic food ($\beta=-0.125$); satisfaction toward organic food significantly leads to IPOF ($\beta=0.513$); perceived barriers significantly moderate (weaken) the relationship between satisfaction toward organic food and IPOF ($\beta=-0.085$).
141	Bryant et al. (2019)	N=3,030 representative samples across the USA ($n=987$), India ($n=1,024$), and China ($n=1,019$).	Online questionnaire survey via CINT research panel, quotas for age and gender, one-way and Welch ANOVAs, LSD post hoc tests, linear regression models.	Plant-based meat and clean meat (cultured meat).	Demographics, familiarity, attitudes across 16 semantic differential attributes, Meat Attachment Questionnaire, Food Neophobia Scale.	Likelihood of purchasing clean meat regularly, likelihood of purchasing plant-based meat regularly.	Yes - Framed around greenhouse gas emissions, deforestation, and freshwater consumption associated with conventional meat, and alternative proteins alleviating environmental harms.	Acceptance of clean and plant-based meat was significantly higher in India and China compared to the USA; food neophobia was higher and meat attachment lower in India; higher familiarity and lower food neophobia consistently predicted higher acceptance across all countries.
142	Razzaq et al. (2021)	N=473 Chinese consumers across two online studies (Study 1: $n=161$; Study 2: $n=312$).	Online questionnaire surveys, focus group pretest, independent sample t-tests, ordered logistic regression analysis, marginal effects, Hayes mediation and moderated-mediation analysis.	Vitamin A (VA) biofortified rice with invisible (white) vs. visible (yellow) nutrition traits, compared to ordinary rice.	Nutrition-related food neophobia (NFN), sensory-affective food neophobia (SFN), demographics, nutrition information on health benefits.	Purchase intentions (PI) for biofortified rice.	Yes - Framed around micronutrient malnutrition ("hidden hunger"), biofortification building sustainable food systems, and bridging agriculture and nutrition.	Biofortified foods with invisible nutrition traits cause nutrition-related food neophobia (NFN), whereas visible traits cause both NFN and sensory-affective food neophobia (SFN); both neophobia types reduce purchase intentions; NFN mediates acceptance for invisible traits, while both NFN and SFN mediate visible traits; providing nutrition information moderates and reduces these neophobic barriers.
143	Wassmann et al. (2024)	N=578 Singaporean consumers (representative quotas for gender and age 20-79).	Online survey, free elicitation task, semantic differential scale (connotative meaning across 7 food products), within-subject ANOVAs, principal component analysis (PCA) with varimax rotation, correspondence analysis, linear regression analyses.	Microalgae-based food products (15 specific microalgae foods/dishes), compared with beef burgers, chicken, tofu, plant-based burgers, seaweed, and insects.	Free associations and attitudes, semantic differential ratings across 7 products, convincingness of microalgae attributes, willingness to buy (WTB) 15 microalgae products, sociodemographics, sustainability/health concerns, food neophobia, meat attachment, social image eating motivation.	Willingness to buy (WTB) microalgae-based "meat/fish substitutes" and "non-substitutes".	Yes - Framed around global food demand, animal protein production driving climate change, resource scarcity, and microalgae as a sustainable alternative protein source with lower land/water footprints.	Most frequent associations with microalgae were "vegetarian/vegan food" and "negative evaluation"; microalgae were perceived similarly to plant-based burgers; "innovative" was rated the most convincing attribute, followed by environmental friendliness and healthiness; WTB microalgae products was positively associated with younger age, being male, higher income, sustainability concerns, health concerns, lack of food neophobia, being a meat reducer, and social image eating motivation.
144	Modlinska et al. (2020)	N=99 participants (81 females, 18 males; average age 22, university students in Poland).	Mixed-design experiment (2×2 ANOVA / ANCOVA), video behavioral coding using BORIS software, product questionnaires, semi-structured qualitative individual interviews with thematic analysis, FNS, GNS,	Three food products (cookie, muffin, date ball) manipulated by label (explicitly stated insect content vs. no insect content) and appearance (presence of visual features suggestive of	Latency to pick up food, latency to begin eating, amount of food eaten (g), time spent eating, frequency of eating bouts, sniffing/looking duration and frequency, water drinking frequency, subjective product	Behavioral acceptance indicators (latency, amount eaten, eating duration/frequency) and product evaluation scores.	Yes - Framed around environmental burden and animal welfare issues associated with conventional livestock production, and insect-based foods contributing to food sustainability and nutritional challenges.	Products labelled as containing insects were picked up later, eaten in smaller quantities, and consumed for shorter times and less frequently, regardless of their appearance; appearance (visual clues of insect parts) did not significantly affect behavioral measures or evaluations; qualitative interviews showed that disgust and lack of cultural custom are

			FTNS, Disgust Sensitivity Scale, VARSEEK scale.	insect content vs. smooth appearance), though none contained insects.	evaluations, psychometric scale scores.			barriers, but familiar pastry formats and safe/hygienic framing can improve openness.
145	Yuan et al. (2026)	N=571 respondents across three top-tier cities in China (Beijing, Shanghai, Guangzhou).	Online choice experiment (CE) survey, random parameter logit (RPL) model in WTP space, log-likelihood ratio tests, pairwise tests, interaction models, kernel density estimation, latent class logit (LCL) model.	500 g packages of chicken breast varying by production method (cultured meat vs conventional meat), carbon trust label, antibiotics use ("no antibiotics ever" vs no info), and price.	Sociodemographics, environmental awareness, animal awareness, food technology neophobia (FTNS), familiarity, prior awareness, willingness to pay (WTP) / marginal WTP (mWTP).	Choice selections in the choice experiment to derive marginal willingness to pay (mWTP).	Yes - Framed around conventional meat production greenhouse gas emissions, arable land and freshwater use, and cultured meat improving environmental sustainability, food security, and animal welfare.	On average, consumers were unwilling to pay a premium for cultured chicken compared to conventional meat; providing information significantly enhanced consumers' mWTP, increasing it by 8.66 RMB/500 g; latent class analysis identified three segments ("Innovators" 68.9%–72%, "Undecided" 16.9%–21.9%, and "Traditionalists" 6.1%–14.2%); younger males with higher environmental awareness and lower food technology neophobia were willing to pay more for cultured meat ("Innovators").
146	Szlachciuk & Żakowska-Biemans (2024)	N=1000 representative consumers by age, gender, and region responsible for household food purchases, Poland	Cross-sectional quantitative online survey, descriptive statistics, Chi-square tests, ANOVA with Scheffé's post hoc test, Exploratory Factor Analysis (EFA) with principal component analysis and varimax rotation	Insects and food products containing insect proteins / added insect proteins (whole visible insects, whole invisible insects, ground insect powder, isolated insect protein)	Sociodemographics, food neophobia (FNS, categorized into neophilic, neutral, neophobic tertiles), beliefs about eating insects (measured via Ruby & Rozin items; 4 extracted factors: disgust, risks, general benefits, private benefits), perception of food products with added insect proteins (semantic differential scale), willingness to eat insect-based foods across different forms	Beliefs about insect consumption, perception of food products with added insect proteins, willingness to eat insects	Yes - Framed around nutritional deficiencies, environmental sustainability, global food insecurity, and insect farming's sustainability (efficient feed conversion, less land/water, fewer greenhouse gas emissions, organic waste utilization).	Many viewed insect-enriched foods as innovative and environmentally friendly, while others found the idea repulsive and cited allergy concerns; food neophobia was a key determining factor in beliefs and perceptions; respondents were more inclined to consume products with added insect protein rather than visibly identifiable insects; neophobic individuals scored higher in disgust and perceived risks, whereas neophilics scored higher in general and private benefits.
147	Rabitti et al. (2024)	N=413 adult participants (18–69 years, 59.8% females) across four European countries (Belgium $n=113$, Germany $n=96$, Italy $n=108$, Sweden $n=96$)	Sensory evaluation laboratory sessions, Check-All-That-Apply (CATA) sensory task (23 attributes), 7-point labelled liking scale, Food Neophobia Scale (FNS), Chi-square tests, Generalized Linear Models (GLM), Bonferroni post-hoc tests, Cochran's Q tests, Sheskin's multiple comparison test, Partial Least Squares Regression (PLSR)	Crackers added with 5% (w/w) powders/proteins of Arthrospira platensis (green Spirulina), isolated protein from Arthrospira platensis (blue Spirulina), Palmaria palmata, Saccharina latissima, Lithothamnium calcareum, compared to a control cracker	Cracker consumption frequency, algae familiarity, knowledge, and past consumption, willingness to try algae-fortified foods, overall liking, CATA sensory attributes (appearance, taste/flavor, texture), Food Neophobia Scale (FNS, segmented into low, medium, high levels), sociodemographics	Overall liking scores, perceived sensory attributes (CATA), drivers of liking and rejection	Yes - Framed around current food system challenges, population growth, resource burdens, and algae as a nutritious and sustainable food source requiring few natural resources.	Crackers added with Lithothamnium calcareum and isolated protein from Spirulina (blue Spirulina) received the highest liking scores (comparable to control), whereas green Spirulina, Palmaria palmata, and Saccharina latissima received lower scores due to off-flavors (fishy, mushroom, grassy) and bitterness; "sweet", "toasted bread", and "bland" contributed positively to liking; Italians showed the highest liking and lowest food neophobia; blue Spirulina successfully reduced off-flavors compared to green Spirulina; Lithothamnium calcareum is highlighted as a promising calcium-rich sustainable algal ingredient.
148	Yang et al. (2020)	N=100 healthy UK adult participants (76 females, 24 males, aged 18–74 years, stratified into three age groups)	Sensory laboratory evaluation across two sessions (blind vs. informed conditions), 10-minute educational presentation/slides on global food security and Bambara groundnut, Labeled Affective Magnitude (LAM) scale for overall liking, EsSense25 questionnaire for emotional responses, Food Neophobia Questionnaire (FNQ), two-factor mixed model ANOVA, Fisher's least significant difference (LSD) tests, Multiple Factor Analysis (MFA), three-factor ANOVA for food neophobia groups	Four snack products across two categories: sweet biscotti (standard commercial biscotti vs. Bambara biscotti where wheat flour was partially replaced with 7% Bambara flour) and savoury crackers (standard commercial crackers vs. Bambara crackers made with 71% Bambara flour and 18% rice flour)	Overall liking, emotional responses (EsSense25 scale across 25 emotions), Food Neophobia Questionnaire (classified into low and medium neophobic groups), extrinsic information impact	Overall liking scores, consumer emotional responses (EsSense25)	Yes - Framed around global challenges (population growth, climate change, water scarcity), sustainable agriculture, and climate-resilient alternative crops like Bambara groundnut that grow on marginal soils and require fewer resources.	Under blind conditions, no significant differences in overall liking were observed between standard and Bambara snack products, indicating UK consumer acceptance of Bambara flour sensory properties; emotional response was more discriminating than liking, with standard products evoking higher calm/joy/happiness in certain dimensions; extrinsic information about global challenges and Bambara's sustainability/nutritional credentials shifted consumer emotional responses toward more positive emotions and made them feel less guilty when consuming Bambara products; food neophobia did not impact acceptability between standard and Bambara products, but medium-neophobic individuals exhibited lower emotional responses overall.
149	Zaman et al. (2026)	N=904 Italian consumers (representative sample by age, gender, and region of residence)	Cross-sectional quantitative online survey, covariance-based structural equation modeling (CB-SEM) using R (lavaan package), pilot study ($n=52$), Cronbach's alpha,	Upcycled foods (UFs) produced through the reuse of food by-products / food waste into value-added ingredients (generic category	Theory of Planned Behaviour constructs (Attitude toward UFs, Subjective Norms, Perceived Behavioural Control, Purchase Intention), Subjective Knowledge of	Consumers' attitudes toward UFs and purchase intention for UF products	Yes - Framed around food waste reduction, circular economy models, increasing resource efficiency, and sustainable consumption.	Environmental concerns ($\beta=1.052$) and subjective knowledge of UFs ($\beta=0.511$) are strong positive attitudinal drivers; traditionality ($\beta=-0.121$) and food neophobia ($\beta=-0.115$) act as weak negative barriers; health consciousness showed

			Average Variance Extracted (AVE), Fornell-Larcker discriminant validity, Chi-Square likelihood ratio tests for the attitude-intention gap	evaluated conceptually)	UFs, Environmental Concerns, Traditionality, Health Consciousness, Food Neophobia, moderation terms for food neophobia			no significant influence; a significant attitude-intention gap exists for upcycled foods, indicating positive attitudes do not fully translate into purchasing intentions; food neophobia acts as a determinant of attitudes rather than an attitude-intention moderator.
150	D'Souza et al. (2025)	N=600 Australian consumers	Survey analyzed using AMOS 28 and SPSS, measurement invariance testing, cluster analysis, multi-group structural equation modelling	Upcycled food products (food waste-based functional products)	Awareness of consequences, ascription of responsibility, self-efficacy, sensory attitudes, personal norms, social norms, behavioral intention, food neophobia, generational/demographic variables, religious commitment	Behavioral intention to choose/purchase upcycled food products	Yes - Framed around global food system sustainability, valorising food by-products into functional ingredients, reducing food waste, and promoting a circular food system.	Ascription of responsibility strongly predicted social and personal norms; sensory attitudes, social norms, and personal norms mediated relationships; neophobic consumers displayed varied responses; Gen X showed lower purchase intentions due to quality concerns; increased age and religious commitment were associated with reduced willingness to purchase upcycled foods.
151	Chang et al. (2026)	N=280 Taiwanese consumers	Structural equation modeling (SEM) analysis of survey data	Plant-based seafood	Value-Belief-Norm (VBN) theory constructs (biospheric values, environmental beliefs, personal moral norms), food neophobia (FN), technological trust (TT), purchase and advocacy intention (PAI)	Purchase and advocacy intention (PAI) toward plant-based seafood	Yes - Framed around climate change, marine resource depletion, overfishing, and sustainable food innovations.	Biospheric values strongly influenced environmental beliefs ($\beta=0.913$), which shaped personal moral norms and purchase/advocacy intention; environmental beliefs mediated between biospheric values and moral norms; technological trust directly predicted purchase and advocacy intention ($\beta=0.528$); food neophobia showed no significant direct effect in the full model but operated as a conditional constraint for inexperienced consumers.
152	Modlinska, Adamczyk, & Pisula (2025)	N=949 Polish participants (online survey)	Online survey evaluating novel food products, ANOVA, linear regression analysis	Novel food product (meatballs) based on insect, meat, or plant protein, presented with or without a safety certification	Overall liking, expected tastiness, willingness to try, market relevance, perceptions of safety, healthiness, environmental friendliness, food neophobia (FNS), food-technology neophobia (FTNS), attention to food information, disgust sensitivity	Product evaluations, willingness to try	Yes - Framed around environmental sustainability and nutritional value of insect-based foods as an alternative to meat.	Insect-based products scored significantly lower than meat and vegetarian options across all evaluations; safety certification yielded only a modest positive effect (primarily in perceived safety and environmental friendliness) with a stronger impact among women; food neophobia and food-technology neophobia predicted more negative assessments (amplified for insects); paying attention to food information predicted more positive responses; disgust sensitivity and food content concerns negatively predicted willingness to try regardless of certification.
153	Placentino et al. (2021)	N=61 Italian professional athletes (27 females, aged 19–39 years) from the Italian Air Force Sports Centre in Rome	Structured questionnaire, descriptive statistics, independent t-tests, Mann-Whitney U test, one-way ANOVA, multiple linear regression, repeated measures ANOVA, Wilcoxon signed-rank test	Energy protein bar enriched with cricket flour	Supplement consumption habits, General and Sport Nutrition Knowledge (A-NSKQ), Food Neophobia Scale (FNS), willingness to taste cricket protein bar (before and after information treatment), motivations for tasting/refusing, sports endorsement willingness	Willingness to taste cricket energy bar, willingness to endorse	Yes - Framed around environmental sustainability (lower environmental impact, fewer resources, reduced CO2 emissions) and nutritional benefits of edible insects.	All subjects consumed supplements (mainly for recovery/performance), though general nutritional knowledge was poor (47.8%); high protein content and texture curiosity were main drivers to taste, while disgust justified rejection; food neophobia increased with age and reduced willingness to endorse; male athletes were more willing to endorse than females; information treatment significantly increased willingness to taste (from 42.6% to 57.4% willing).
154	Vanutelli et al. (2024)	N=175 Italian university students (final sample n=158 after quality checks)	Online questionnaire survey via Qualtrics, snowball sampling, General Decision-Making Style Inventory (GDMS), Trust in Science and Scientists Inventory (TSSI), Food Neophobia Scale (FNS), Implicit Association Test (IAT), explicit semantic differential attitudes, intention to taste items, cluster analysis (Ward's hierarchical and k-means), univariate ANOVAs	Insect-based foods (grasshopper flour, cricket burger, larvae cookies, insect crackers)	Explicit attitudes (semantic differential scale), automatic attitudes (IAT D-index), intention to taste IBFs, food neophobia, trust in science and scientists, general decision-making styles (rational, intuitive, dependent, avoidant, spontaneous)	Explicit attitudes, automatic attitudes (IAT), intention to taste IBFs	Yes - Framed around facing climate crisis, starvation, environmental footprint, and sustainable diets/alternative protein sources.	Overall sample showed negative explicit attitudes and pro-traditional food automatic bias (IAT mean D=0.61); cluster analysis identified 4 decision-making profiles ("the gut feeling", "the suspicious", "the vicarious", "the mind"); "the mind" (rational style, high trust in science) showed the most favorable explicit attitudes and intention to taste, while "the suspicious" (high neophobia, low trust in science) showed aversive reactions; implicit attitudes did not differ significantly across clusters, highlighting persistent implicit resistance.
155	Rombach & Dean (2024)	N=437 consumers mirroring the New Zealand population in terms of gender, age, and annual household income	Online questionnaire survey, descriptive statistics, partial least squares structural equation modeling (PLS-SEM).	Macro-algae (seaweed) and macro-algae-based foods.	Symbolic value of food, health importance, food safety concerns, food fussiness tendencies, willingness to eat macro-algae-based foods, perceived	Willingness to eat macro-algae-based foods, perceived trust towards countries of origin.	Yes - Framed around climate change, negative environmental externalities, food insecurity, and macro-algae as a sustainable plant-based protein source.	Health importance and food fussiness tendencies are the strongest predictors of willingness to eat and trustworthiness of countries of origin; New Zealand consumers trust both Indonesia and the Pacific Islands as macro-algae producers; food safety and

		(collected November 2023).			trust towards countries of origin (Indonesia, Pacific Islands), sociodemographics.			symbolic value showed non-significant relationships in certain paths.
156	Zhang et al. (2016)	N=400 households sampled across six residential communities in Zhongguancun Sub-District, Haidian District, Beijing, China.	Quantitative household survey (in-person interviews in March 2013), descriptive statistics.	Fresh agricultural produce / food provisioning schemes (conventional wet markets/supermarkets vs. alternative food networks like self-provision, CSA, direct sales, specialized retail).	Consumer participation in food-provisioning schemes, factors affecting food purchase choices (convenience, freshness, price, safety), trust in various food-provisioning schemes, trust in guarantees, information sources, supervision actors.	Consumer participation and trust levels across food provisioning schemes.	Yes - Framed around urban environmental/health challenges in Beijing, sustainable food provisioning, and reducing environmental impacts.	Only a small proportion of urban middle-class consumers regularly use alternative food-provisioning schemes; most rely on conventional wet markets and supermarkets due to convenience, freshness, and price; trust in food safety information remains primarily derived from the government rather than market certification or civil society.
157	You & Choi (2026)	N=1,247 South Korean consumers	Online survey, Principal Component Analysis (PCA) with varimax rotation, Latent Class Analysis (LCA), multinomial logistic regression, split-sample validation, bootstrap resampling, cross-validation.	Smart farm produce / controlled environment agriculture (CEA) / vertical farming / hydroponic vegetables.	Technology Acceptance Model (TAM) constructs (perceived usefulness, perceived ease of use, attitude, behavioral intention), Food Technology Neophobia Scale (FTNS), local food values (origin attachment, terroir appreciation, authenticity seeking), purchase intention, demographic and behavioral covariates.	Consumer market segments (latent classes) and purchase intentions toward smart farm products.	Yes - Framed around climate change, rapid urbanization, global population growth, water conservation, reduced transportation distance, and sustainable food production.	Identified the "local food paradox" where sustainable/local food advocates resist smart farming due to perceived lack of authenticity and terroir; identified three distinct consumer segments: Technology Enthusiasts (37.0%), Conflicted Moderates (25.0%), and Tradition-Oriented Skeptics (38.0%); organic buyers and farmers' market shoppers were more likely to belong to the skeptical group.
158	Muiruri (2024)	N=8,633 usable responses from three rounds of a nationally representative Norwegian survey (2019/2020, 2021/2022, 2023/2024; total initial respondents across rounds N=10,718).	Confirmatory Factor Analysis (CFA) for BFI-20 personality traits, Generalized Structural Equation Modelling (GSEM) with ordinal logit distribution, mediation analysis, multigroup analyses (MGA) with Wald tests.	Food made from insects (entomophagy).	Willingness to try (WTT) food made from insects, trust (social trust, trust in food authorities, trust in food retailers), food safety concerns, food choice motives (environment, health, natural ingredients, novelty, familiarity), OCEAN personality traits (Big Five BFI-20), sociodemographics.	Willingness to try (WTT) food made from insects.	Yes - Framed around sustainable food consumption, environmental and health impacts of meat production, and entomophagy as a sustainable alternative meat substitute.	About 40% of Norwegians were unwilling to try insect food, 31% somewhat willing, and 29% willing (stable over 2019–2024); social trust and trust in food authorities were positively associated with WTT; food safety concerns negatively affected WTT and acted as a significant mediator; environmental friendliness, health, and novelty positively affected WTT, while natural ingredients and familiarity had negative associations; openness was positively associated with WTT, whereas conscientiousness, extraversion, and agreeableness had negative associations.
159	Silva et al. (2026)	N=245 Brazilian consumers segmented into three theoretical profiles (Explorers G1 $n=99$, Achievers G2 $n=79$, Guardians G3 $n=67$).	Mixed-methods design, Text Highlighting Technique, pre/post perception scales (5-point Likert), Mann-Whitney U tests, r-biserial effect sizes.	Dahlia (Dahlia spp.) as an unconventional food plant (UFP) / edible flower food ingredient.	Consumer profiles (Explorers, Achievers, Guardians based on age brackets and attitudes), perception items (allergy/safety concerns, perceived healthiness, sensory contribution, sustainability, interest in trying), text highlighting polarity indices.	Consumer perceptions, evaluations, and acceptance/interest regarding dahlia flowers as a food ingredient.	Yes - Framed around nutritional and sensory attributes, biodiversity, health, and sustainability / low environmental impact of dahlia as an unconventional food plant.	Exposure to structured information significantly reduced safety and allergy concerns while enhancing positive evaluations of innovation and sustainability; Achievers exhibited the most receptive attitudes, Explorers were responsive but sensitive to risk cues, and Guardians remained cautious, prioritizing safety and institutional credibility.
160	Annunziata, Kraus, & Mariani (2026)	N=335 Italian university students from a university in southern Italy.	Online survey questionnaire, descriptive statistics, binary logistic regression analysis (enter method), pseudo-R^2, Hosmer-Lemeshow goodness-of-fit test.	Cultured meat (CM) / cultivated, lab-grown, or cell-based meat.	Meat-eating habits, future meat reduction intention, animal welfare concerns in daily food choices, familiarity and knowledge of CM, perceptions of CM attributes (naturalness, safety, nutritional content, taste, price, sustainability benefits), Food Neophobia Scale (FNS), Food Technology Neophobia Scale (FTNS), sociodemographics.	Willingness to try (WTT) cultured meat (binary: high vs. low willingness).	Yes - Framed around environmental sustainability, greenhouse gas emissions, deforestation, biodiversity loss, resource efficiency, and animal welfare of conventional livestock vs. cultured meat.	Acceptance of cultured meat among university students is driven primarily by familiarity, perceived safety, and ethical considerations (particularly animal welfare), rather than by demographic characteristics, price, perceived nutritional value, or resistance to novel food technologies (neophobia/technophobia).
161	Yang, Ren, Sun, & Gu (2025)	N=799 valid college student respondents across China (survey conducted Oct–Nov 2023).	Online questionnaire survey, exploratory factor analysis, Cronbach's alpha reliability, corrected item-total correlation (CITC), confirmatory factor analysis (CFA) using AMOS	Alternative foods / insect protein foods / insect specialty foods / alternative protein sources.	Theory of Planned Behavior constructs (Social Influence, Food Safety, Nutritional Confidence, Commitment to Sports, Dietary Habits, Satisfaction, Attitude,	Customer satisfaction, attitude, and purchase intention toward alternative/insect foods.	Yes - Framed around climate change, land scarcity, food safety challenges, environmental pollution, and sustainable development goals (zero hunger).	Social influence, food safety, and dietary habits significantly influence college students' satisfaction, attitudes, and purchase intentions when purchasing alternative foods; attitude and purchase intention are positively correlated with satisfaction; nutritional

			V22.0, common latent factors method (CLF) for common method bias, convergent/discriminant validity (HTMT, Fornell-Larcker), structural equation modeling (SEM).		Purchase Intention).			confidence and attitude are significant predictors of purchase intentions; sport commitment did not show a significant relationship in this model; food safety is established as a critical prerequisite for alternative food acceptance.
162	Chuah et al. (2025)	N=2,121 US food shoppers nationwide (online survey via Qualtrics).	Nationwide online survey, exploratory factor analysis (Maximum Likelihood Analysis with Varimax rotation) on seafood attitudes and Domain Specific Innovativeness (DSI), binary logit model, Check-All-That-Apply (CATA) questionnaires, Total Unduplicated Reach and Frequency (TURF) analysis.	Cell-based seafood (lab-grown, cultured, or cultivated seafood).	Attitudes towards conventional seafood, Domain Specific Innovativeness, seafood consumption habits, awareness of cell-based seafood, socio-demographics (age, gender, race, ethnicity, region, community, income, education), barriers preventing adoption (artificialness, unfamiliarity, safety concerns, lack of regulation, lack of exposure, price, etc.), positive attributes for TURF analysis (food safety, affordability, health benefits, animal welfare, sensory, sustainability, alternative protein).	Preferences/willingness to try cell-based seafood.	Yes - Framed around global food security, overfishing, ocean ecosystem degradation, sustainability, and seafood demand doubling by 2050.	Consumers open to innovation and concerned about environmental impact of conventional seafood showed higher preference for cell-based seafood, as did existing seafood consumers, younger generations (Gen Z vs. Boomers/Gen X), females, and higher-educated individuals; artificialness and unfamiliarity were primary barriers; TURF analysis revealed that food safety was the most compelling attribute (reaching 80.1% of consumers), and pairing it with affordability captured 93% of consumers.
163	Bogueva & Marinova (2025)	N=520 Australians aged 18 to 64.	Mixed-methods approach, online survey using LimeSurvey, quantitative descriptive/categorical analysis, qualitative open-text coding and visualization using NVivo 14.	Alternative proteins including plant-based meats, plant-based milks, insect-based proteins, cultured meat, and algae-based products.	Dietary habits/behaviors, past experience/trial and future consumption intent for alternative proteins, perceived safety, nutritional value, health risks (additives, ultra-processing, allergens, high sodium, GMOs), cost, transparency, masculinity perceptions, and environmental/ethical considerations.	Trial and willingness/intention to incorporate alternative proteins into diets.	Yes - Framed around environmental consequences of traditional meat production (greenhouse gas emissions, land use, water consumption) and sustainable food consumption.	Plant-based milk (74.8% trial) and algae (91.9% trial) are widely accepted; plant-based meat trials are moderate (57.3%) but future regular consumption is very low (1.5%); insect-based (21.5% trial) and cultured meat (0.2% trial) face strong aversion; over 53% expressed dissatisfaction with taste/texture; key barriers include high processing/additives, food safety/regulatory concerns, nutritional deficiency fears, and traditional ideas of masculinity associated with meat consumption.
164	Panghal et al. (2024)	N=211 consumers in India (quantitative online surveys and personal visits).	Literature review, structured questionnaire (40 items on a 5-point Likert scale), Exploratory Factor Analysis (EFA) using Principal Component Analysis (PCA) with Varimax rotation and Kaiser normalization via IBM SPSS, Cronbach's alpha reliability testing, KMO and Bartlett's test of sphericity.	Blockchain technology (BCT) enabled sustainable food supply chains and traceable agricultural/food products.	40 items related to consumer perceptions of blockchain in food supply chains, food safety, traceability, transparency, sustainability, health, trust, and switching intentions.	Factors strengthening consumer trust and willingness to accept a blockchain-enabled food system.	Yes - Framed around sustainable food production and consumption (SFPC), SDG 12 (Responsible Consumption and Production), reducing food waste/pollution, and enhancing resource efficiency across food supply chains.	EFA classified the 40 items into five key underlying factors strengthening consumer trust and acceptance: Reliability (F1), Sustainability (F2), Impact on Health (F3), Trust (F4), and Switching Intentions (F5); Cronbach's alpha was 0.9; KMO was 0.964; consumers showed strong willingness to switch to traceable, blockchain-enabled food systems due to enhanced transparency, safety assurance, and resource utilization.
165	Pawelczyk et al. (2026)	N=949 Spanish consumers.	Between-subject experimental design survey, exploratory factor analyses (EFAs), multiple regression models with interaction terms, robust standard errors.	Mycoprotein (fungi-based meat alternatives / FBMAs).	Information treatments (control vs. health-related vs. environment-related info), Food Technology Neophobia Scale (FTNS), dietary patterns (omnivorous vs. restrictive), knowledge, prior experience, general attitude toward mycoprotein, sociodemographics (gender, age, education, income).	Consumer acceptance (ACC) / attitude toward mycoprotein.	Yes - Framed around sustainable protein alternatives, reducing environmental burdens, climate change, land use, and lower ecological footprint of mycoproteins.	Information treatments alone did not significantly increase acceptance; food technology neophobia was strongly negatively related to acceptance; prior experience, knowledge, attitudes, and restrictive dietary patterns were positively related to acceptance; information treatments moderated the negative effect of food technology neophobia.
166	Baum et al. (2023)	N=158 individuals in the United States.	Online survey via Qualtrics (2019), partial least-squares structural equation modeling (PLS-SEM) using SmartPLS 3.3, BCa bootstrapping.	CRISPR food / gene-edited (GE) crops and foods.	Perceived benefits, perceived risks, social trust, Food Technology Neophobia Scale (FTNS), New Ecological Paradigm (NEP) subscales (Dominant Social Paradigm [DSP] and New Environmental Paradigm [NEnvP]).	Behavioral intentions towards CRISPR food.	Yes - Framed around transition to a more sustainable food and agricultural system, climate change, population growth, and food/nutritional insecurity.	Established predominance of perceived benefits vis-à-vis perceived risks for explaining behavioral intentions; perceived benefits have a positive, large-effect impact on behavioral intentions, while perceived risks are not significant; indirect effects of social trust, food technology neophobia, and environmental worldviews transmitted through perceived benefits (and risks).

167	Giacalone & Jaeger (2023)	N=2,494 participants across Australia (n=623), India (n=615), Singapore (n=627), and the USA (n=629).	Online survey, check-all-that-apply (CATA) questions for emotional/conceptual associations and situational appropriateness, willingness-to-consume (WTC) frequency scale, Kruskal-Wallis and Wilcoxon rank sum tests, Correspondence Analysis, PCA, Hierarchical Clustering.	12 novel sustainable food technologies across product categories (plant-based milk/cheese, modified atmosphere vegetables, urban farming vegetables, aquaponics fish, cell-cultured fish/meat, plant-based meat blends, insect powder meat blend, gene-edited bread).	Willingness-to-consume (WTC), emotional and conceptual associations, situational appropriateness, Food Technology Neophobia (FTN), environmental concern (ENV), socio-demographics, dietary status.	Willingness-to-consume (WTC) frequency for individual technologies.	Yes - Framed around reducing the environmental burden of agri-food systems (greenhouse gas emissions, biodiversity loss, freshwater use, eutrophication, land system change, chemical pollution) and transitioning toward sustainable production and diets.	Major differences in consumer acceptance grouped into high (urban farming/modified atmosphere vegetables), medium (aquaponics fish, plant-based alternatives, gene-editing), and low acceptance (insect-containing meat, cell-cultured meat/fish); Indian consumers responded more positively than Western consumers; four pan-national consumer segments identified primarily by baseline WTC; only ~20% in high acceptance segment, showing widespread general skepticism toward food technologies.
168	Jiang et al. (2024)	N=1,245 consumers across five major cities in China (Beijing, Shanghai, Wuxi, Wuhan, Shenzhen).	Discrete Choice Experiment (DCE), fractional factorial design, between-subject information treatment design (control vs. environmental information), Random Parameter Logit (RPL) model in WTP space, Epanechnikov kernel density estimation, hierarchical regression models, EGA.	Prepared plant-based meat (PPBM) products, specifically frozen meatballs.	Product attributes (raw material category, certification labels, quality and safety statements, brands, price), willingness to pay (WTP), Food Technology Neophobia Scale (FTNS), time preference, socio-demographics.	Consumer choice and willingness to pay (WTP) for prepared plant-based meat products.	Yes - Framed around greenhouse gas emissions from animal husbandry (beef as a major source), environmental pressure, and sustainable transformation of the food industry.	Environmental information provision significantly increased consumer preferences and WTP for frozen meatballs containing mixed meat and pure plant-based meat, as well as organic and green labels, but decreased WTP for quality/safety attributes; consumers with high food technology neophobia had lower WTP for plant-based meat, whereas impatient consumers (higher time preference) had higher WTP for PPBM products.
169	Fu et al. (2023)	N=410 omnivore consumers in the United States (nationwide survey via Dynata).	Dual-factor model, online survey, pilot testing/pretesting, Confirmatory Factor Analysis (CFA), Exploratory Graph Analysis (EGA), Structural Equation Modeling (SEM).	Cultivated meat (clean meat, cultured meat, in vitro meat, lab-grown meat).	Facilitators (physical health, animal welfare, food quality), inhibitors (unnaturalness, food technology neophobia), sustainable substitutability as a substitute for conventional meat, purchase intention, sociodemographics.	Evaluation of cultivated meat as a sustainable substitute and purchase intention.	Yes - Framed around conventional meat production environmental damage (freshwater scarcity, air/water pollution, biodiversity loss, greenhouse gas emissions) and sustainable development.	Physical health, animal welfare, and food quality significantly encouraged consumer acceptance of cultivated meat as a sustainable substitute; food technology neophobia significantly inhibited acceptance, whereas unnaturalness did not show a significant impact; evaluation as a sustainable substitute significantly enhanced consumers' purchase intention.
170	Kaczmarek (2026)	N=425 adult Polish meat eaters (cross-sectional online survey).	Cross-sectional online survey, reliability and validity testing (Cronbach's alpha, McDonald's omega, HTMT, Harman's single-factor test, correlated uniqueness sensitivity analysis), binary logistic regression, K-means cluster analysis, bootstrap validation (ARI), linear discriminant analysis (LDA).	Cultured meat (CM / cultivated, cell-based, or lab-grown meat).	Awareness of cultured meat, attitudes toward cultured meat (ATT_CM), behavioural intentions toward cultured meat (INT_CM), technological risk and naturalness concern (TRNC), general acceptance of cultured meat (GACM), conventional meat-purchase motivations, sociodemographics.	Attitudes, general acceptance, and behavioural intentions toward cultured meat.	Yes - Framed around environmental impact, sustainability of conventional livestock systems, animal welfare, and the global protein transition.	Overall attitudes and acceptance were moderately positive, while technological risk and naturalness concerns persisted; four psychographic segments identified (cautious optimists [35.9%] and concerned ambivalents [33.3%] as largest groups); conventional meat-purchase motivations showed statistically significant but modest associations with cultured meat attitudes (ethical/environmental motives weakly positive, sensory-oriented motives weakly negative); limited structural correspondence between meat-purchase motivations and cultured meat psychographic clusters; younger, urban, and higher-educated respondents were more favourable.
171	Castellini, Graffigna, et al. (2026)	N=2,416 Italian adults (cross-sectional online survey representative of the Italian adult population).	Cross-sectional online survey via CAWI, between-subject design (participants randomly exposed to one of three technologies), contingency tables, Pearson's chi-square test, one-way ANOVA with Bonferroni post-hoc comparisons, Pearson correlations, hierarchical linear regression models, Harman's single-factor test.	Three sustainable food technologies in agriculture and their treated foods: upcycling technology (upcycled food), biostimulants (biostimulant-treated food), and artificial intelligence (AI in agriculture).	Knowledge, specific beliefs about technologies and novel foods, intention to buy novel foods, socio-demographics, psychosocial variables (health consciousness, environmental concern, conspiracy mentality, food technology neophobia, psychological food involvement, variety seeking, trust in agri-food system actors).	Intention to buy novel foods treated with the respective technologies.	Yes - Framed around food systems impact on environmental sustainability, greenhouse gas emissions, biodiversity decline, water and land use changes, chemical pollution, and the need for sustainable production methods.	Limited consumer knowledge (80% never heard of upcycling, 67% biostimulants, 50% AI); AI applied in agriculture was better known but perceived as less natural and riskier; upcycled foods perceived as sustainable but low in pleasantness; biostimulants occupied a middle ground; differences between technologies were small; psychological and attitudinal factors (variety seeking, food technology neophobia, food involvement) and trust had stronger associations with purchase intentions than socio-demographics.
172	Cerjak & Faletar (2025)	N=402 respondents in Croatia.	Online survey, exploratory factor analyses (EFA), multiple regression analysis, two-step cluster analysis, binary logistic regression analysis.	Cultured meat (lab-grown meat).	Food Technology Neophobia Scale (FTNS), Animal Ethics Orientation Scale (AEOS), willingness to consume cultured meat (WTC), and socio-economic characteristics.	Willingness to consume (WTC) cultured meat.	Yes - Framed around livestock farming environmental impacts (land and water resource competition) and animal welfare.	Mean willingness to consume cultured meat was 3.12 (uncertain/polarized, ~40% willing); FTNS factors significantly influenced WTC (positive views increased willingness, while critical views and restraint decreased it); animal ethics orientation had no significant influence on WTC; two consumer clusters identified based on neophobia; current place of residence, religiosity, and political affiliation

								predicted cluster membership.
173	Dupont, Harms, & Fiebelkorn (2022)	N=497 participants in Germany.	Online questionnaire via SoSci Survey, descriptive statistics, Spearman's correlations, t-tests, mediation analysis via PROCESS, path model analysis using R-Studio (lavaan package, MLR estimation).	Cultured meat burger.	Theory of Planned Behaviour constructs (general attitudes towards cultured meat, specific attitudes towards a cultured meat burger, perceived behavioural control, subjective norms), nutritional-psychological variables (food neophobia, food technology neophobia, food disgust, sensation seeking, green consumption values), sociodemographic data, meat consumption/reduction, familiarity with cultured meat.	Willingness/intention to consume a cultured meat burger.	Yes - Framed around the diet-environment-health trilemma, livestock greenhouse gas emissions (14.5%–51%), arable land usage (71% for feed), and sustainable nutrition.	58.4% reported willingness to consume a cultured meat burger; extended TPB accounted for 77.8% of variance in willingness to consume; perceived behavioural control was the strongest predictor, followed by specific attitudes and subjective norms; general attitudes' influence was fully mediated by specific attitudes; food technology neophobia was the strongest negative predictor of attitudes, while green consumption values were the strongest positive predictor.
174	Yaşa Özeltürkay et al. (2025)	N=324 participants across Turkey.	Online survey via Google Forms (March–April 2024), Partial Least Squares Structural Equation Modeling (PLS-SEM) using SmartPLS 4.0, Harman's single-factor test, out-of-sample prediction via CVPAT.	Genetically modified (GM) foods.	Food technology neophobia (FTN), perceived knowledge (PK), perceived benefits (PB), perceived risks (PR), attitudes towards GM foods (AGM), intention to consume GM foods (IC).	Intention to consume/purchase GM foods.	Yes - Framed around climate change, population growth, urbanization, natural resource depletion, Sustainable Development Goal 12 (Responsible Consumption and Production), Zero Hunger, and Climate Action.	Food technology neophobia reduces perceived benefits and increases perceived risks; perceived knowledge enhances perceived benefits and lowers perceived risks; perceived benefits positively affect attitudes while perceived risks negatively affect them; positive attitudes toward GM foods strongly influence purchase intentions (explaining 52.5% of variance).
175	Lührmann, Akinmade, & Weinrich (2026)	N=701 Nigerian consumers (online survey, July 2025).	Online survey with bot protection, partial least squares structural equation modeling (PLS-SEM) using SmartPLS4, measurement and structural model assessment, CVPAT out-of-sample prediction, multiple linear regressions.	Cultured meat (CM / cultivated, cell-based, synthetic meat).	Familiarity, interest, green consumption values (GCV), food technology neophobia (FTN), food disgust sensitivity (FDS), social norms (SN), perceived barriers (PB), perceived motives (PM), meat consumption frequency, sociodemographics (age, gender, income, education, urban/rural location, religion/Islam).	Acceptance of cultured meat (composite score of willingness to try, purchase, eat regularly, and replace farmed meat).	Yes - Framed around population growth in Nigeria, rising meat consumption pressures on environmental systems, greenhouse gas emissions, biodiversity loss, desertification, and farmer-herder conflicts.	Strong market potential among educated, high-income urban consumers (66.19% willingness to try, 52.35% willingness to purchase, though lower for regular consumption [33.10%] and replacement [31.10%]); acceptance is positively predicted by individual interest, social norms, and perceived motives, and negatively predicted by perceived barriers, food disgust sensitivity, food technology neophobia, and income; disgust toward CM is the strongest barrier, while environmental benefits are the strongest motive (explaining 80.8% of variance).
176	Kilimann et al. (2026)	N=420 participants in Germany.	Online survey, Partial Least Squares Structural Equation Modelling (PLS-SEM), pre-registered study.	Precision fermentation-derived Gouda (animal-free cheese).	Self-oriented beliefs (safety, nutritiousness, taste), societal-oriented beliefs (climate change, animal welfare, world hunger), attitudes toward purchasing, purchase intention, environmental self-identity, trust in food sector stakeholders, food technology neophobia (FTN).	Attitudes toward purchasing and purchase intention for PF-derived Gouda.	Yes - Framed around environmental and ethical challenges of livestock farming, increasing global protein demand, and sustainable food production.	Both self-oriented and societal-oriented beliefs positively affected consumers' attitudes, with self-oriented beliefs playing a dominant role; attitude positively affected purchase intention; food technology neophobia positively moderated the relationship between self-oriented beliefs and attitudes.
177	Baum, De Steur, & Lagerkvist (2023)	N=727 potential consumers in Germany (representative sample).	Experimental online survey (between-subjects design: text-only, familiar images, high-tech/laboratory images, control), Single-Target Implicit Association Test (ST-IAT), explicit attitudes semantic differential, abbreviated Food Technology Neophobia Scale (FTNS), Preference for Intuition and Deliberation (PID) scale, logistic quantile regression.	Cultured meat.	Behavioral intentions (willingness to try, regularly purchase, prefer over conventional, pay more), explicit attitudes, implicit attitudes (D-scores), food technology neophobia (FTN), preference for intuition and deliberation (PID), meat-substitute familiarity, meat-eating frequency, political views, educational attainment, children at home, information treatments.	Behavioral intentions towards cultivated meat (log-transformed).	Yes - Framed around negative environmental impacts of conventional meat production and prospective benefits of cultivated meat.	Information treatments (details on benefits and different image types) showed no significant main effect on behavioral intentions or attitudes; food technology neophobia was consistently the strongest negative predictor across all quantiles (strongest for those rejecting CM); significance of covariates varied across quantiles (e.g., deliberative thinking stronger among rejecters, intuitive thinking and implicit attitudes relevant for neutral/positive individuals); linear regression overestimates general explanatory power and masks local variations across the distribution.
178	Mariano et al. (2026)	N=139 Brazilian consumers residing in the Federal District.	Quantitative, applied, and explanatory survey approach, partial least squares structural equation modeling (PLS-SEM)	Cultivated meat (enabled by emerging technologies such as 3D printing and bioprinting).	Physical health (SF), animal welfare (BEA), food quality (FQ), perceived unnaturalness (NN), food	Perceived sustainable substitutability and purchase intention toward cultivated meat.	Yes - Framed around growing demand for animal-based protein intensifying environmental, sanitary, and ethical pressures on food	Animal welfare and physical health significantly and positively influenced consumer evaluation of cultivated meat as a sustainable substitute; food technology

			using SmartPLS 4, bootstrapping (5000 resamples), G*Power sample size calculation.		technology neophobia (NF), perceived sustainable substitutability (SU), purchase intention (IC), sociodemographics (age, income).		systems, greenhouse gas emissions, freshwater consumption, and sustainable alternatives.	neophobia had a significant negative effect; perceived sustainable substitutability served as a robust mediator between antecedents and purchase intention; moderating effects of age (on animal welfare and food quality relationships) and income (on food technology neophobia) evidenced consumer response heterogeneity.
179	Pagnani et al. (2026)	N=405 consumers across Italy (nationwide online survey).	Nationwide online survey, exploratory factor analysis (PCA), hierarchical cluster analysis (HCA) using Ward's linkage method and squared Euclidean distances, ANOVA, Pearson's Chi-square tests.	Upcycled apple-based foods / foods enriched with apple pomace (biscuits, cakes, yogurt, crackers, bread, pasta, salami).	Food Involvement Scale (FIS), Food Responsibility (FRL), Health domain of Meaning of Food in Life Questionnaire (MFLQ), Food Neophobia Scale (FNS), Food Technology Neophobia Scale (FTNS), circular economy and upcycled food knowledge, microbiological risk concern, purchase motivations (environmental sustainability, UN 2030 Agenda, apple pomace recovery, appreciation of farmers' labour/efforts, health, taste, fibre, children), willingness to try/pay, socio-demographics.	Willingness to try and purchase upcycled apple pomace foods.	Yes - Framed around food waste reduction, circular economy promotion, resource conservation, greenhouse gas emissions, and sustainable food systems.	Three consumer clusters identified: sustainability & ethical food advocates (36.3%, young educated women), mainstream consumers with moderate interest (39%, gender-balanced, older/rural), and young, rural & food-sceptical consumers (24.7%); key purchase drivers included environmental sustainability, health, taste, and a notable ethical dimension (appreciating farmers' work and dedication); sweet baked goods (biscuits, cakes) received highest acceptance, while savory items (salami, pasta) faced resistance; awareness of circular economy and upcycled foods was low.
180	Kuff et al. (2023)	N=256 Brazilians who are gym users and consume conventional sports supplements.	Online survey via Google Forms, extended Theory of Planned Behavior (TPB) framework, Partial Least Squares Structural Equation Modeling (PLS-SEM) using SmartPLS 3.3.3.	Insect-based protein bars and powders (sports supplements enriched with cricket/buffalo powder).	Attitude, subjective norms, intention to consume, health beliefs, taste, sustainability, perceived risk, expert recommendations (health professionals), media recommendations.	Intention to consume insect-based sports supplements (protein bars and powders).	Yes - Framed around sport nutrition demands, health/well-being, and environmental attributes of edible insects (lower greenhouse gas emissions, high feed-to-protein conversion rates, low water requirements compared to conventional livestock).	Perceived risk outperformed attitude as a predictor, negatively diminishing the intention to consume insect-based sports supplements; health was the most important explanatory factor of attitude, followed by a smaller effect from sustainability (taste impact was not significant); media recommendations were a strong predictor of subjective norms, whereas experts (health professionals) were not significant.
181	Kamrath et al. (2025)	Study 1: N=463 German consumers; Study 2: N=154 German university students at the University of Bonn.	Study 1: Online survey (October 2018), quota sampling, Partial Least Squares Structural Equation Modeling (PLS-SEM), PLS-MGA multigroup analysis. Study 2: Online survey experiment with 4 information treatment groups and a control group, multiple linear regression, independent-sample t-tests, mediation analysis via PROCESS macro.	3D-printed food.	Subjective knowledge, trust in institutions (general, calculative, and affective trust), perceived benefits (convenience, naturalness, socioeconomic, environment, health), perceived risks, perceived behavioral control, attitude, subjective norm, information credibility and usefulness, socio-demographics, food involvement, ecological worldview, innovativeness.	Intention to consume 3D-printed food.	Yes - Framed around new food technologies providing sustainable food innovations and transitions toward a more sustainable food system.	FTAM and TRA models demonstrated good explanatory power (TRA R^2=0.673, FTAM R^2=0.608 for intention); subjective knowledge, trust in institutions, perceived benefits, perceived risks, attitude, and subjective norm significantly predicted consumption intention; perceived behavioral control lacked discriminant validity and was excluded; Study 2 revealed that affective trust significantly predicts calculative trust both directly and indirectly through information credibility, while information provision alone did not alter calculative trust.
182	Mustapa et al. (2025)	N=127 Spanish consumers participating in sensory laboratory sessions in Barcelona, Spain.	Face-to-face online survey and sensory evaluation session in a sensory lab (ISO 8589:2007), Becker-DeGroot-Marschak (BDM) mechanism incentive-compatible auction method, exploratory factor analysis (EFA), paired-sample t-tests, multinomial logistic regression (MNL) model.	Innovative legume-based quick-frozen gnocchi made from pea, red lentil, and chickpea flours.	Sociodemographic characteristics, attitude variables (government support, health consciousness, environmental concerns, trust in plant-based products, perceived benefits, perceived risks, attitude toward plant-based products), sensory characteristics (color, smell, flavor, appearance, texture, global acceptance), willingness to pay (WTP) before (perception) and after tasting (evaluation).	Willingness to pay (WTP) and WTP categories (low, moderate, high WTP) for legume-based gnocchi.	Yes - Framed around plant-based foods, environmental sustainability of livestock farming, EAT-Lancet Commission planetary health diet, and environmental advantages of legume cultivation (lower water/fertilizer needs, soil properties).	Over 80% expressed initial WTP, but tasting the products resulted in negative disconfirmation (lowering WTP post-tasting); average WTP post-tasting was €5.44 for chickpea, €4.96 for pea, and €4.86 for red lentil gnocchi; multinomial logit regression showed that education level, monthly income, financial situation, environmental concerns, government support (for chickpea), perceived risks, color, and flavor significantly influenced consumers' WTP.
183	Li, Van Loo, Bai, & van	N=1,532 Chinese consumers across 23	Between-subjects online survey experiment (randomized	Cultured meat (CM).	Perceived naturalness, perceived disgust, perceived	Consumer attitudes toward cultured meat.	Yes - Framed around potential environmental merits of cultured	Cultured meat and "cell-based meat" were significantly more appealing and rated higher

	Trijp (2024)	provinces (urban and rural areas).	assignment to three name-framing conditions: "cultured meat", "artificial meat", "cell-based meat"), one-way ANOVA with Games-Howell post-hoc tests, mediation analysis via PROCESS macro (bootstrap confidence intervals with 10,000 samples).		personal and societal benefits, perceived personal and societal risks (integrated into "benefits" and "risks" measures), consumer attitudes toward consuming CM.		meat such as reducing natural resource use (land, water, energy, fodder) and livestock pollution.	in perceived benefits and naturalness, while evoking less disgust than "artificial meat"; name framings affected benefit perceptions more than risk perceptions; perceived disgust and perceived naturalness acted as crucial mediators between name framings and attitudes, with perceived disgust fully mediating the effect of naturalness on perceived benefits and risks (affect heuristic processing); perceived risks were not a significant mediator in cognitive processing.
184	Hsiao & Tang (2026)	N=311 potential users in Taiwan recruited via online social media communities (Facebook and LINE groups).	Three-month online survey evaluation of a case surplus food app ("Tasteme"), questionnaire based on scenario and interface presentations, common method bias checks (Harman's single-factor test, full collinearity VIF test), Partial Least Squares Structural Equation Modeling (PLS-SEM) using SmartPLS 4.1.	Tasteme (mobile app connecting stores offering edible surplus food with nearby consumers).	Altruism value (AV), convenience value (CV), monetary value (MV), perceived risk (PR), awareness of consequence (AC) regarding food waste.	Three distinct behavioral intentions: purchase intention (PI), app adoption intention (AI), and recommendation intention (RI).	Yes - Framed around urban food waste challenges, Sustainable Development Goals, circular economy solutions, and pro-environmental awareness.	Value-driven factors (altruism, convenience, monetary value) and awareness of consequences significantly enhance purchase, app adoption, and recommendation intentions (model explains 42.2% to 52.5% of variance); perceived monetary value influences purchase and recommendation intentions but not app adoption intention; perceived risk significantly reduces purchase intention but has little effect on app adoption and recommendation intentions, indicating that risk perceptions mainly affect transaction-related intentions rather than platform engagement.
185	Cantalapiedra, Juan-García, & Juan (2023)	N=235 higher-education students (undergraduates and graduates in health sciences, nutrition, pharmacy, gastronomy, food science, veterinary) from universities in Valencia, Spain.	Observational, descriptive, cross-sectional survey study via Google Forms (academic years 2021/2022 and 2022/2023), binary Yes/No questions and 5-point Likert scale questions, Pearson's Chi-Squared test, Student's t-test, ANOVA with Tukey HDS post-hoc test.	Edible insects / novel insect-based foods.	Dietary habits/previous experience, cultural influences, gastronomic perception, food safety perception, knowledge of nutritional and health contributions (protein, minerals, anti-nutrients), marketing and labeling preferences (quality seals, EU certification, ingredient labeling), sociodemographics (gender, age, university studies).	Perception and acceptance of edible insects as a novel food source.	Yes - Framed around sustainable protein sources, dietary diversification, food and nutrition security, and smaller ecological impact.	Only 18% had tasted insects, but >84% showed favorable attitudes toward trying novel foods and insects; students recognized high nutritional and protein content; men showed greater willingness to cook insects at home, eat them in restaurants, and include them in daily diets compared to women; participants preferred clear ingredient labeling, EU/quality certification seals, and transparent information; marketing campaigns and knowledge were seen as major influencers of acceptance, while food safety concerns were prominent regarding wild-harvested versus industrially processed insects.
186	Li, Kallas, & Rahmani (2022)	N=1,006 consumers in China.	Semi-structured online survey (June 2020), Multiple Price List (MPL) lottery approach for risk attitude, binary logistic regression models.	Food purchasing and consumption behavior during the COVID-19 lockdown.	Sociodemographic variables, Multiple Price List stated risk attitude (lotteries), risk perceptions (health, financial, food security risks), health concerns about COVID-19, experience during COVID-19 (food shortages, price increases), subjective and objective knowledge regarding COVID-19, sustainable purchasing attributes (local, animal welfare, fair-trade, organic), healthy/sustainable diet adoption, household food waste changes.	Changes in purchasing food with sustainable attributes (P), sustainable/healthy diet adoption (D), and home food waste changes (W).	Yes - Framed around sustainable food consumption, greenhouse gas emissions (GHGE), land, water, and energy use, biodiversity, and reducing food waste.	Females and consumers aged 40-59 were more likely to increase purchases of food with sustainable attributes; risk-neutral and risk-averse consumers were less likely to increase sustainable food purchases; health risk and food security risk perceptions increased healthy diet adoption and food waste (due to stockpiling/panic buying); objective and subjective knowledge regarding COVID-19 increased healthy diet adoption; single-member households and unhealthy individuals experienced higher food waste increases.
187	Cui et al. (2026)	N=362 Taiwanese consumers (online survey, Oct–Nov 2025).	Confirmatory Factor Analysis (CFA), Structural Equation Modeling (SEM) using AMOS, Harman's single-factor test, reliability and convergent/discriminant validity testing.	Ultra-processed foods (UPFs).	Health risk perception, environmental burden perception, Theory of Planned Behavior constructs (attitude, subjective norms, perceived behavioral control), health involvement, environmental awareness, behavioral intentions (reducing UPF consumption), sociodemographics (age, education, income).	Intention to reduce ultra-processed food consumption.	Yes - Framed around global food supply chain greenhouse gas emissions (26%), energy-intensive processing, cold-chain logistics, single-use plastic packaging, and sustainable diets (SDG 12).	Both health and environmental risk perceptions significantly and positively shape attitudes toward reducing UPF intake (with health risk perception exerting a stronger effect); attitude, subjective norms, and perceived behavioral control significantly increase reduction intentions (subjective norms being the strongest predictor); environmental awareness produces a diminishing marginal effect, negatively moderating the relationship between environmental burden perception and behavioral intention.
188	Mao et al. (2025)	N=1,261 U.S. consumers	Online survey via Qualtrics, quota sampling, linear	Upcycled food (specifically, milk	Perceived risk, perceived benefit, subjective knowledge	Consumer attitudes toward upcycled food.	Yes - Framed around food waste management pathways, reintegrating	Perceived risk exerts a significant negative impact on attitudes, while perceived benefits

		(nationally representative sample matched to U.S. Census for age, gender, and region).	regression analysis, PROCESS macro (Model 1) for moderation analysis.	enriched with peanut skins).	of upcycled food, subjective knowledge of food waste, social consumption motivation, trust in food producers, consumer attitudes toward upcycled food.		by-products into the food production chain, reducing food loss, and circular economy solutions.	exert a positive effect (outweighing risk perception); subjective knowledge of upcycled food directly predicted attitudes; two heuristic factors (social consumption motivation, trust in food producers) and two systematic factors (subjective knowledge of upcycled food, subjective knowledge of food waste) significantly moderated and weakened the negative effect of risk on attitudes.
189	Qi et al. (2024)	N=353 valid respondents in China (collected via online and offline sources, total 375 collected).	Reliability and validity analysis, exploratory and confirmatory factor analysis (CFA), analysis of variance (ANOVA), multiple comparisons, structural equation modeling (SEM) using SPSS and AMOS.	Triploid food (crops and aquatic species produced using triploid breeding technology).	Demographic variables (sex, age, education level, employment, place of residence), fear degree of triploid food, social trust, food safety concern, risk perception of triploid food.	Consumers' risk perception of triploid food.	Yes - Framed around sustainable food, new food processing/breeding technologies, and agricultural stress resistance/yield enhancement.	Consumers' concerns regarding triploid food primarily revolve around health risks rather than ecological risks; significant differences in risk perception were found based on age, educational background, place of residence, and employment status (e.g., elderly and lower-educated individuals exhibited higher risk perceptions, while students showed the lowest); fear and social trust were negatively correlated with risk perception, whereas food safety concerns were positively correlated with risk perception.
190	Legendre & Baker (2020)	N=423 U.S. consumers (nationally representative online panel).	Self-administered cross-sectional online survey, descriptive statistics, Shapiro-Wilk normality tests, measurement model analysis (outer model) via PLS-SEM (SmartPLS 3.2.7), structural model analysis (inner model), simultaneous mediation analysis.	Edible insect food products / entomophagy.	Trust on regulators, risk perception, overall benefit perception (higher-order factor comprising environmental benefits, health benefits, economic benefits), purchase activism, purchase intention.	Purchase intention and purchase activism regarding edible insect foods.	Yes - Framed around nature-based solutions (NBS) to global food security, decreasing arable land, greenhouse gas emissions, land/water use, and long-term sustainability.	Grounded in the legitimacy judgment process model; trust in food regulators negatively affects risk perception and positively affects overall benefit perception; risk perception negatively affects purchase activism and purchase intention, while overall benefit perception positively affects both; purchase activism positively affects purchase intention; risk and benefit perceptions and purchase activism act as significant mediators.
191	Castellani et al. (2025)	N=167 respondents in Italy (convenience sampling).	Online questionnaire via SurveyMonkey, partial least squares structural equation modeling (PLS-SEM) using SmartPLS 4.1.0.0, bootstrapping (10,000 subsamples), PLSpredict algorithm for out-of-sample predictive power.	Lab-grown meat (cell-based / cultured meat).	Attitudes towards consuming lab-grown meat, environmental benefits, subjective norms, perceived risks, purchase intentions.	Intention to purchase lab-grown meat.	Yes - Framed around Sustainable Development Goal 12 (sustainable consumption and production), global population growth, livestock greenhouse gas emissions, land/water use limitations, and environmental sustainability.	Environmental benefits strongly and positively influenced attitudes (path coefficient 0.707) and indirect purchase intentions (0.605); perceived risks had a negative effect on attitudes (-0.213); attitudes strongly predicted purchase intentions (0.856, explaining 74.9% of variance); subjective norms did not have a significant effect on purchase intentions; model demonstrated high explanatory power and predictive validity.
192	Xu et al. (2024)	N=580 valid respondents (out of 641 collected) in Shanghai, Nanjing, Wuhan, and Xi'an, China.	Questionnaire survey using the Contingent Valuation Method (CVM) with a payment card approach, one-way ANOVA, exploratory factor analysis, Structural Equation Modeling (SEM) using AMOS 26.0, latent variable interaction terms, bias-corrected nonparametric percentile Bootstrap method for mediation.	Five carbon-labeled agricultural products (CAP): milk, corn, bananas, tomatoes, and eggs.	Awareness of consequences (AC), ascription of responsibility (AR), attitudes toward the behavior (ATT), low-carbon awareness (LA), perceived behavioral control (PBC), personal knowledge of CAP (PK), personal norms (PN), risk perception (RP), subjective norms (SN), willingness to pay (WTP), purchase intention (PI), purchase behavior (PB).	Willingness to pay (WTP) premium amount, purchase intention (PI), and purchase behavior (PB).	Yes - Framed around food systems responsible for 26–34% of global greenhouse gas emissions, IPCC climate targets, low-carbon consumption, carbon labels indicating greenhouse gas emissions, and reducing carbon footprints.	Consumers' willingness to pay a premium for carbon-labeled milk, corn, bananas, tomatoes, and eggs is 27.50%, 29.73%, 26.86%, 26.51%, and 24.26%, respectively; perceived behavioral control has the strongest positive influence on purchase intention, followed by subjective norms, attitudes, and personal norms; risk perception negatively moderates the relationship between purchase intention and purchase behavior (intention-behavior gap); awareness of consequences indirectly influences personal norms through ascription of responsibility.
193	Chen et al. (2023)	N=647 valid urban residents across seven Chinese cities (Zhejiang, Shanghai, Shandong, Anhui, Hubei, Shaanxi, and Gansu).	Online questionnaire survey via WJX.cn, reliability and validity analysis, exploratory factor analysis, structural equation modeling (SEM) to test the "awareness-situation-behavior" model, path analysis, interaction and moderation analysis.	Man-made meat (meat substitutes / plant-based / cultivated meat).	Climate change and energy issue perception (CCEIP), low-carbon awareness (LA), social responsibility awareness (SRA), man-made meat risk perception (MMRP), man-made meat information disclosure (MMID) (measured via knowledge of brands, ingredients, nutrition, price), intention to consume man-made meat (MMCI).	Intention to consume man-made meat (MMCI).	Yes - Framed around global climate change, carbon emissions, carbon peaking and carbon neutrality ("dual carbon") goals, and dietary carbon footprints.	Low-carbon awareness, personal social responsibility awareness, and man-made meat risk perception significantly influence the public's intention to consume man-made meat, with risk perception having the greatest negative influence (-0.434); low-carbon awareness and risk perception have a significant interaction effect on consumption intention; man-made meat information disclosure significantly moderates the relationship between low-carbon awareness and consumption intention, as well as between risk perception and consumption intention.
194	Harrison & Liem (2026)	N=1,540 Australian adult consumers	Cross-sectional online questionnaire survey	Cultivated meat (CM) / cellular agriculture	Interpersonal trust, institutional trust	Acceptance / willingness to buy cultivated meat (CM).	Yes - Framed around sustainable protein sources, animal agriculture	Epistemic trust, institutional trust (authorities), and trust in technology oversight correlated

		(recruited via Deakin University sensory database, social media, and flyers).	(Compusense), exploratory factor analysis (Principal Component Analysis with varimax rotation), Pearson's correlation, multiple linear regression, parallel mediation analysis using PROCESS macro v4.2 with bootstrapping (5,000 resamples), non-parametric Mann-Whitney U tests.	products.	(authorities), institutional trust (food), epistemic trust (trust in CM-related science), trust in CM technology oversight, perceived benefits of CM, perceived risks of CM, willingness to accept / buy CM, sociodemographics/individual characteristics (rurality, education, gender, age, diet).		land use (80% of agricultural land), greenhouse gas emissions (57% from livestock), freshwater use, biodiversity, and animal welfare.	significantly with acceptance of CM (trust in technology having the strongest correlation, r=0.537); parallel mediation analysis revealed that perceived benefits and risks mediate the relationship between trust domains and CM acceptance (total indirect effects via perceived benefits generally exceeded those via perceived risks); perceived benefits positively impacted acceptance (b=0.44–0.58), while perceived risks negatively impacted acceptance (b=-0.34 to -0.39); younger age groups, university-educated individuals, and major city residents displayed higher trust, perceived benefits, and acceptance.
195	Pareti et al. (2025)	N=59 participants across seven cities in five administrative regions in China (Beijing, Shanghai, Changsha, Jinan, Linyi, Urumqi, Ili).	Qualitative focus group interviews (7 sessions, ~90 mins each), thematic coding (open, axial, and selective coding via NVivo 12), co-occurrence analysis of nodes using UCINET 6.365 and NetDraw.	Cell-cultured meat (animal cell-cultured meat) and plant-based meat.	Consumer knowledge, consumer perceptions (ecological environment protection, resource conservation, animal welfare, cleanliness, nutritional diversity, famine alleviation, food safety, laws/regulations, unnatural taste/texture, monopoly/livelihood losses), potential acceptance/willingness to try and buy.	Consumer perceptions and potential acceptance of meat substitutes.	Yes - Framed around environmental pollution, grassland degradation, greenhouse gas emissions, carbon peak and neutrality goals, and sustainable food systems.	Chinese consumers generally exhibit low awareness and knowledge of meat substitutes, especially cultured meat; while participants acknowledged environmental, resource-saving, and animal welfare benefits, they expressed strong concerns about artificial taste, high costs, food safety, market monopolization, and impacts on traditional animal husbandry; willingness to try (driven by curiosity) was much higher than willingness to regularly purchase; participants showed a clear preference for plant-based meat over cultured meat; legislative support, technical advancement, price reduction, and traceability systems are crucial for enhancing consumer confidence and acceptance in China.
196	Abebe et al. (2024)	N=1,800+ responses (1,563–1,580 analyzed across models) from a nationally representative sample of Canadian consumers across all regions.	National survey via Angus Reid panel (proportional stratified sampling, online via Qualtrics), Multivariate Probit (MVP) model, Multinomial Logit (MNL) model, exploratory factor analysis for scale reliabilities.	Alternative protein foods (APF): plant-based (PB) milk, plant-based (PB) meat, lab-grown (LG) meat, and other alternative proteins (OAP like insect and fungal proteins); compared with animal-based foods (ANBF): milk and meat.	Innovation-adoption characteristics (IAC: perceived complexity, perceived relative advantage, perceived trialability, perceived compatibility), perceived risks (health/nutrition, processing, additives, calories, sodium), meat attachment, sustainability orientation, demographic and contextual controls (gender, age, children, education, income, neighborhood, region).	Current adoption / consumption of APF categories and intentions to alter dietary patterns (ANBF and APF) in the next 12 months.	Yes - Framed around greenhouse gas emissions, land and water use of animal agriculture, climate change mitigation, resource efficiency, and sustainable protein transitions.	PB milk was the most consumed APF (57%), followed by PB meat (39%), while lab-grown meat was the least tried (5.7%); perceived complexity discouraged individuals from cutting back on animal-based foods while increasing APF consumption; perceived relative advantage positively predicted APF adoption; perceived compatibility strongly influenced PB meat and OAP adoption; perceived trialability significantly influenced PB milk and PB meat adoption; health and nutritional perceived risks did not prevent current PB milk and OAP consumption but affected future dietary alteration intentions; meat attachment and sustainability orientation significantly predicted current consumption decisions and future dietary intentions.
197	Li, Wang, & Yang (2022)	Study 1: N=180 young Chinese participants; Study 2: N=200 students and MBAs in northern China; Study 3: N=288 WeChat community members; Study 4: N=292 online participants.	Four empirical studies, ANOVA, independent t-tests, mediation analysis via PROCESS (Model 4), moderated mediation analysis (Model 7).	Plant-based meat (brands: Plant Diary, Beyond Meat, Protein Meat).	Social media marketing intensity, cognitive fluency, customer inspiration, perceived risk, brand community identity (in-group vs. out-group), purchase intention.	Purchase intention.	Yes - Framed around food production and consumption as major contributors to environmental issues, improving animal welfare, reducing environmental pollution, and achieving carbon neutrality.	Social media marketing enhances young people's purchase intention more than traditional marketing; cognitive fluency mediates the relationship between marketing intensity and purchase intention; higher cognitive fluency increases customer inspiration and purchase intention, whereas lower cognitive fluency increases perceived risk and reduces purchase intention; brand community identity moderates these pathways (in-group members show higher cognitive fluency and customer inspiration, while out-group members show higher perceived risk).
198	Shaw et al. (2026)	Pre-study: n=52 Australian residents; Main study: N=990 Australian residents (906 meat eaters, 94 vegetarians).	2(product type: plant-based meat vs. meat) x 2(message appeal: rational vs. emotional) between-subjects experimental design, pre-study paired-samples t-tests, moderated-mediation analysis (PROCESS Model 7 with 5,000 bootstrap samples), simple mediation	Plant-based meat vs. meat (burgers).	Perceived risk (functional, financial, social, physical, psychological, and time risk dimensions), perceived value (quality, emotional, price, and social value dimensions), message appeal (rational vs. emotional), purchase intentions, word-of-mouth	Purchase intentions and Word-of-Mouth (WOM).	Yes - Framed around environmental, animal welfare, and sustainability concerns associated with meat consumption and the need to shift toward alternative food products.	Regardless of message appeal, meat eaters perceived plant-based meat as riskier and less valuable than meat, reducing acceptance; functional, financial, physical, psychological, and time risks (but not social risk) and all four value dimensions significantly mediated the relationship between product type and behavioral responses; for younger meat-eaters, emotional message appeals increased meat

			analyses.		(WOM), product involvement, age/generation.			acceptance through quality value, whereas for vegetarian consumers, rational message appeals enhanced perceived quality value for plant-based meat products, leading to higher purchase intentions and WOM.
199	Gao et al. (2026)	Study 1: N=1,154 participants across four Chinese cities (Beijing, Shenzhen, Zhengzhou, Chengdu); Study 2: N=746 valid participants (pretest: n=128 students; formal experiment: N=746) in Beijing and Zhengzhou.	Study 1: Questionnaire survey, Structural Equation Modeling (SEM) using Amos 26 based on the SOR framework; Study 2: 2x3 between-subjects factorial experiment, two-way ANOVA, ANCOVA controlling for environmental concern.	Near-expired bread (suboptimal food / near-expired food).	Price discounts, time remaining until expiration, normative beliefs regarding food waste avoidance, moral satisfaction, perceived risk, perceived value, purchase intentions, product attitudes, environmental external benefits, environmental concern (covariate).	Purchase intentions (Study 1 & 2), product attitudes (Study 2), and environmental external benefits (Study 2).	Yes - Framed around reducing food waste, green consumption, conserving ecological resources, and sustainable food systems.	Price discounts and longer remaining shelf life increase purchase intentions by enhancing perceived value and reducing perceived risk; normative beliefs with regard to food waste avoidance positively predict purchase intentions through moral satisfaction; price discounts significantly influence purchase intentions and product attitudes, while information framing (gain vs. loss) affects purchase intentions and environmental external benefits; an interactive effect between price discounts and information framing shapes perceived environmental external benefits; environmental concern significantly predicts all outcome variables.
200	Ali et al. (2026)	Study 1: N=397 U.S. participants; Study 2: N=735 U.S. participants (recruited via Prolific).	Study 1: Pre-registered experimental study, linear mixed-effects models, multilevel parallel mediation analysis; Study 2: Pre-registered 2x2 mixed/between-subjects experimental design (Food Type: Original vs. Replica x Food Category: Natural vs. Processed), linear mixed-effects models, multilevel moderated mediation analysis.	Cultured foods across meat (beef, chicken, salmon), plant-based (cacao, coffee, vanilla), and dairy/egg (cheese, butter, egg) categories; compared with processed snack foods (Twinkies, Clif bars, Doritos) and their independent technological replicas.	Naturalness, willingness to try, perceived risk, disgust.	Willingness to try.	Yes - Framed around cellular agriculture, mitigating environmental and ethical costs of conventional food production, and reducing greenhouse gas emissions and agricultural land pressure.	Participants consistently reported lower willingness to try cultured foods compared to traditionally sourced counterparts across all food types; risk and disgust mediated this effect, with risk emerging as a general driver of aversion across meat, dairy/egg, and plant-based cultured products; Study 2 demonstrated that replicating natural foods is judged as riskier and more aversive than replicating human-made processed snack foods, and disgust arises only for cultured versions of natural foods, suggesting that aversion reflects broader risk perceptions associated with tampering with and replicating natural processes.
201	Arango, Chaudhury, & Septianto (2023)	Study 1: N=181; Study 2: N=361; Study 3: N=594 participants located in the United States (recruited via Prolific).	Three experimental studies, one-factor and two-way ANOVAs, mediation analysis (PROCESS Model 4), moderated mediation analysis (PROCESS Models 6 and 7 with 5,000 bootstrap resamples).	Cultured meat (chicken nuggets and beef burger patties).	Scarcity appeals (demand-based vs. supply-based vs. no-scarcity/control), perceived risk (general, physical, psychological, and social risks), perceived naturalness (heightened vs. non-heightened), perceived availability.	Willingness to try cultured meat.	Yes - Framed around population growth, conventional meat production pressures, deforestation, greenhouse gas emissions, water depletion, and sustainability.	Demand-based scarcity appeals outperform supply-based and no-scarcity appeals in promoting cultured meat by reducing perceived risk (via social proof); this effect is moderated by perceived naturalness, being especially effective when consumers perceive cultured meat as unnatural; Study 3 confirmed specific dimensions like physical and psychological risks.
202	Szentesi et al. (2026)	N=631 respondents in Romania.	Quantitative online survey via Google Forms, variance-based structural equation modeling (PLS-SEM) using SmartPLS 4.	Sustainable food products / food products with alternative ingredients.	Perceived food-related misinformation on social media (MISINFO), agentic AI use for food information verification and decision support (AAI), perceived nutritional value (PNV), trust in food-related information and products (TRUST), sustainable food purchase intention (INTENT).	Sustainable food purchase intention (INTENT).	Yes - Framed around sustainable consumption, Sustainable Development Goal 12 (responsible consumption and production), agri-food economic system resilience, and sustainable diets.	AI assistance strengthens both nutritional benefit evaluation (PNV) and trust (TRUST), whereas social media misinformation undermines them; purchase intention is explained primarily through perceived nutritional benefit evaluation, while trust plays a complementary uncertainty-reducing role; PNV and trust partially mediate the relationships between digital antecedents and purchase intention.
203	García, Nieto-Villegas, & Rabadán (2026)	N=1,218 consumers across Spain (408), the United Kingdom (402), and Germany (408).	Online survey (Qualtrics) featuring a discrete choice experiment (DCE) with D-optimal efficient design, mixed logit (MIXL) model in WTP space, latent class logit (LCL) model for consumer heterogeneity.	Watermelon produced using different genetic selection and pesticide strategies.	Genetic selection (precision-bred organism vs. no info), pesticide use (conventional, organic-certified, zero-residue), production method (outdoors vs. greenhouse), price, consumer attitudes (health attitudes/HTAQ, New Ecological Paradigm/NEP, food technology neophobia/FTNS, chemophobia), sociodemographics.	Consumer preferences and marginal willingness to pay (mWTP) / choice.	Yes - Framed around environmental degradation from global food systems, land use, fresh water use, greenhouse gas emissions, and sustainable agricultural practices.	22–27% of consumers across Spain, UK, and Germany reject precision-bred watermelon; conventional pesticides are perceived as significantly riskier than precision breeding or alternative pesticide strategies; consumers show a stronger preference for zero-residue pesticides over organic-certified alternatives; latent class analysis identifies a safety-oriented segment (~22–27%) that strongly rejects precision breeding and pays high premiums for zero-residue/organic pesticides, while a majority are neutral or unconcerned.
204	Vanutelli et al.	N=311 Italian older	Between-subjects online	Three alternative protein	Intention to consume (ITC),	Intention to consume (ITC)	Yes - Framed around sustainable	Insect-based food was the least favored option

	(2025)	adults (ages 65–75, 50.5% female).	experimental survey via Bilendi platform, multiple linear regression models, Implicit Association Test (IAT) for automatic attitudes, one-way ANOVAs with Welch's/Fisher's tests and post-hoc comparisons.	sources (APS): plant-based foods (PBF), cultured meat (CM), and insect-based foods (IBF).	explicit attitudes (tastiness/disgust, safety/risk), automatic attitudes (IAT D-index), familiarity / tasting experience, sociodemographics.	alternative protein sources.	protein sources supporting planetary well-being and healthy aging, contrasting with the environmental impacts of conventional meat production.	overall; for PBF, familiarity was the strongest predictor of consumption intention, followed by taste and automatic attitudes; for cultured meat, intention was associated with explicit attitudes regarding both taste and safety; for insect-based foods, intention was primarily related to taste attitudes; unexpectedly, while PBF was rated explicitly as tastier and safer than cultured meat, it received less favorable automatic attitudes and consumption intentions, suggesting older adults may implicitly resist plant-based meat substitutes compared to novel animal-based options like cultured meat.
205	Yu & Chang (2025)	N=515 valid online respondents in Taiwan.	Anonymous web-based questionnaire survey via SurveyCake, Harman's single-factor test for CMV, reliability/validity analysis, structural equation modeling / path analysis using SPSS 23 and SmartPLS 3.	Upcycled foods.	Environmental knowledge (EK), environmental risk perception (ERP), credibility of product environmental quality (CPEQ), deontological evaluation (DE), teleological evaluation (TE), ethical judgment (EJ), purchase intentions (PI).	Purchase intentions (PI) toward upcycled food.	Yes - Framed around food shortages, food waste reduction, circular food systems, natural resource strain, greenhouse gas emissions, and environmental sustainability.	Applying an ethical decision-making framework (Hunt & Vitell model) in an Eastern cultural context (Taiwan), the study reveals that moral philosophies (deontological and teleological evaluations) play a central role in shaping consumers' ethical judgments and purchase intentions toward upcycled food; environmental knowledge, environmental risk perception, and product environmental quality credibility significantly influence these moral evaluations; deontological evaluations exert a stronger influence on ethical judgment than teleological evaluations.
206	Basarir & Dayan (2022)	N=400 consumers in the United Arab Emirates (UAE).	Questionnaire survey via stratified random sampling (paper-based, web-based, social media, personal interviews), Harman's single-factor test, confirmatory factor analysis (CFA), structural equation modeling (SEM) using SmartPLS.	Sustainable / innovative food products (functional foods, extended shelf-life foods, fortified foods, nanotechnology foods).	Innovative product characteristics / contextual factors (relative advantage, compatibility, complexity, trialability, observability), perception of innovation, perceived cost/benefit, perceived risk and uncertainty, attitude strengths (cognitive, affective, behavioral), demographics.	Consumer adaptation / acceptance of food innovations.	Yes - Framed around sustainable innovations in the food industry considering environmental and social issues, future generations, and health/environmental benefits.	Consumer perception of sustainable food innovations is directly and significantly affected by product characteristics (relative advantage, complexity, and observability, while compatibility and trialability were non-significant in direct paths); perception of innovation mediates the relationship between these product characteristics and consumer adaptation; adaptation is further influenced by perceived cost/benefit, perceived risk & uncertainty, and multi-dimensional attitude strengths (cognitive, affective, behavioral).
207	Lee & Lee (2024)	Experiment 1: N=189 undergraduate students; Experiment 2: N=143 undergraduate students at a large midwestern U.S. university.	Two online experiments (Experiment 1: 2x2 within-subjects factorial design; Experiment 2: 2x2x2 mixed-factorial design including sensation seeking), repeated measures ANOVA, pairwise comparisons.	Cell-cultured meat (news posts on social media).	Message frame (societal benefits vs. individual benefits), food cue (direct vs. indirect), sensation seeking (high vs. low, via Brief Sensation Seeking Scale), perceived risk, positive affect, negative affect, attitude, purchase intention.	Perceived risk, attitude toward cultured meat, and purchase intention.	Yes - Framed around environmental and ethical impacts of meat production, greenhouse gas emissions, land and water use, and animal welfare.	Individual benefit-framed messages result in more positive affect, lower negative affect, and lower perceived risk compared to societal benefit-framed messages; direct food cues (realistic photos) lead to lower risk perception, more favorable attitude, and higher purchase intention than indirect food cues; sensation seeking moderates message effects (low sensation seekers respond more favorably to direct food cues and individual benefit frames).
208	Kubacak et al. (2022)	N=238 valid responses from a nationally distributed U.S. sample (recruited via Marketing Systems Group / MSG).	Between-subjects experimental design with researcher-developed blog posts (opposition, neutral, support), Meltwater sentiment analysis, one-way ANOVAs, post-hoc Bonferroni comparisons.	Lab-grown meat.	Message evaluation, intention to share content, risk perception, benefit perception, uncertainty perceptions, intention to consume, political views, demographics.	Message evaluation, intention to share, risk perception, benefit perception, uncertainty, and intention to consume lab-grown meat.	Yes - Framed around global population growth, rising meat demand, environmental impacts of traditional agriculture, and greenhouse gas emissions.	Message theme has a statistically significant effect on risk perception, benefit perception, intention to share content, and intention to consume (with opposition themes increasing risk and decreasing consumption/sharing intent), but no significant effect on message evaluation or uncertainty perceptions.
209	Taillie et al. (2021)	N=1,235 US red meat consumers (mean age 44 years) recruited via CloudResearch Prime Panels.	National online survey, randomized experiment with four label conditions (no-label control, health warning, environment warning, combined health and environment warning), choice experiment with burritos, logistic and OLS regressions.	Red meat products (steak burrito, bacon, sausage, pizza, cheeseburgers, ground beef, deli ham) with front-of-package warnings.	Label conditions, perceived message effectiveness, believability, negative emotions, perceived risk, attention, learning new information, red meat consumption reduction intentions, consumption frequency, climate change beliefs.	Preferred item selection in choice experiment (steak burrito), item perceived most damaging to health/environment, message effectiveness, and consumption reduction intentions.	Yes - Framed around greenhouse gas emissions from beef production, climate change mitigation, and planetary health.	Warnings did not significantly affect burrito preference overall, but health warnings increased recognition of red meat as damaging to health (73%); combined and health warnings outperformed environment warnings on perceived effectiveness, believability, negative emotions, and perceived health harm; warning impact was stronger among frequent red meat consumers.
210	Cascone & Timpanaro (2026)	N=2,127 Italian adult consumers (nationally	Computer-Assisted Web Interviewing (CAWI) survey, exploratory factor analysis	Enabling technologies in sustainable agriculture / products obtained	Experiential dimensions (Awareness, Risk Perception, Environmental Concern,	Experiential consumption intention.	Yes - Framed around agriculture 4.0, climate resilience, reducing environmental impacts, resource	EFA identified four latent dimensions (Awareness, Risk Perception, Environmental Concern, Trust in Benefits); cluster analysis

		representative CAWI survey).	(EFA) with Varimax rotation, k-means cluster analysis using silhouette width index, robust OLS linear regression model.	through sustainable agricultural technologies (Key Enabling Technologies - KETs / agriculture 4.0).	Trust in Benefits), experiential consumption intention (single-item indicator), socio-demographics.		optimization, and sustainable agri-food production transitions.	revealed four consumer segments (Informed Skeptics, Eco-Optimists, Green Doubters, Pragmatic Tech-Adopters); Pragmatic Tech-Adopters, Eco-Optimists, and Green Doubters reported significantly higher experiential consumption intention than Informed Skeptics; income was positively and age negatively associated with experiential intention.
211	Pareti et al. (2025)	N=2,241 valid respondents (out of 2,647 distributed) in China.	Online questionnaire survey via Wenjuanxing, descriptive statistics, correlation analysis, SPSS 27, Hayes' PROCESS macro for moderated dual-mediation analysis with bootstrapping (5000 resamples).	Meat substitutes (plant-based protein meat and cultured meat).	Consumer trust (in manufacturing companies, regulatory agencies, media, traceability), consumer knowledge (types, industry development, manufacturing tech, pricing), perceived benefits (environmental protection, resource conservation, animal welfare, nutrition), perceived risks (health effects, unnatural taste, agricultural impact, monopoly), consumer acceptance (willingness to purchase).	Consumer acceptance (willingness to purchase meat substitutes).	Yes - Framed around greenhouse gas emissions, carbon peaking and neutrality ("dual carbon") goals, grassland preservation, and environmental pressures of animal husbandry.	Consumer trust positively influences acceptance, enhances perceived benefits, and significantly reduces perceived risks; perceived benefits and perceived risks partially mediate the relationship between trust and acceptance (indirect effects exceed direct effects); consumer knowledge negatively moderates the trust-perceived risk relationship (higher knowledge amplifies trust's risk-reduction effect) and negatively moderates the trust-perceived benefit relationship.
212	Fechner et al. (2026)	Study 1: n=484 Prolific participants in Australia; Study 2: n=45 eye-tracking experiment.	Survey experiment (Study 1), eye-tracking experiment (Study 2), chi-square homogeneity tests, logistic regression, multiple linear regression.	Plant-based meat burger in a restaurant menu.	Video intervention (manufacturing process video vs. mug video vs. availability video), informational familiarity, perceived risk (naturalness, health, ingredient unfamiliarity), curiosity, visual attention (gaze time, fixation count), food neophobia, food technology neophobia, meat ordering habit, experiential familiarity, age, gender.	Stated selection of plant-based meat burger.	Yes - Framed around hospitality sector progress toward net zero, food systems driving climate change, greenhouse gas emissions, and plant-based diets as mitigation strategies.	Watching a video about the manufacturing process increases simulated ordering of plant-based meat burgers from 5% to 15% by increasing curiosity, rather than lowering perceived risk or directing attention; perceived risk theory did not predict food choices in this hedonic context, while exploratory consumer behavior (curiosity) did; eye-tracking data showed visual attention (gaze bias effect) predicted choice, but the video did not increase attention.
213	Ma et al. (2025)	N=3,756 valid online questionnaires collected from 25 provinces and 3 autonomous regions in China.	Nationwide online survey via JiShuYun platform, Generative Pre-trained Transformer (GPT) models for automated open-ended/survey analysis, Partial Least Squares Structural Equation Modeling (PLS-SEM).	Alternative proteins / four new protein-based food products: plant-based foods, cell-cultured meat, insect-based proteins, and microbial proteins.	Food technophobia (FTN), food values (FV), perceived benefits (PB), perceived risks (PR), consumer acceptance, demographic characteristics (DC).	Consumer acceptance of plant-based foods, cell-cultured meat, insect-based proteins, and microbial proteins.	Yes - Framed around global population growth, unsustainable traditional food production, alternative proteins offering solutions to food security, environmental sustainability, and animal welfare.	Acceptance is highest for plant-based foods and microbial proteins and lowest for cell-cultured meat; perceived benefits positively and perceived risks negatively impact consumer willingness; mediation analysis reveals that perceived risks and benefits mediate the effects of food technophobia and food values on acceptance; demonstrates the capability of GPT models for automated, large-scale consumer food survey analysis.
214	Hartmann et al. (2025)	Study 1: N=9,578 valid responses across 7 European countries (France, Germany, Hungary, Italy, Norway, Serbia, United Kingdom); Study 2: N=498 participants in Germany.	Study 1: Two pan-European consumer surveys, exploratory factor analysis (EFA), paired sample t-tests, OLS regressions with robust standard errors; Study 2: Single-factor between-subjects experiment, ANOVAs, Bonferroni post-hoc tests, serial mediation analysis using Hayes & Preacher (2014) approach with Mplus (bootstrapping with 5,000 samples).	EU organic "green leaf" logo (original vs. modified logos adding "ECO" or "ECO EU certified").	Label design conditions (original vs. Modification 1 ["ECO"] vs. Modification 2 ["ECO EU certified"]), label perception factor scores, uncertainty reduction, label trust, behavioral intention to use the label.	Label perception, uncertainty reduction, label trust, and behavioral intention to use the label.	Yes - Framed around food systems greenhouse gas emissions, tropical deforestation, biodiversity loss, land degradation, water scarcity, and sustainability transitions.	Logo modifications that improve signal clarity induce more favorable label perceptions across all seven European countries (Study 1); Study 2 uncovers the underlying mechanism: logo modifications reduce consumer uncertainty, which in turn significantly improves label trust and ultimately consumers' behavioral intentions to use the label in purchase decisions.
215	Proi, Coderoni, & Perito (2025)	N=800 participants representing the Italian population (quota sampling by gender, age, and region).	Online survey via IPSOS online panel (December 2023), ordered logistic regression model, marginal effects analysis.	Cultured meat.	Willingness to try (WTT) cultured meat, willingness to eat (WTE) regularly, willingness to substitute farmed meat with cultured meat; risk attitudes (DOSPERT scale, attitude to food in general, attitude to	Willingness to try (WTT), willingness to eat regularly (WTE), and willingness to substitute farmed meat with cultured meat.	Yes - Framed around environmental and animal welfare impacts of industrial animal farming, natural habitat loss, tropical deforestation, and sustainable food systems.	49% are willing to try cultured meat, 37% willing to eat regularly, and 38% willing to substitute farmed meat; at lower commitment (WTT), gender, prior knowledge, eating habits, meat importance, risk attitudes, conservative political tendencies, and comparative product perceptions predict acceptance; at higher commitment (substitution), comparative

					new food, food safety), political affiliation (right-left orientation, liberal-conservative scale), perception of cultured meat compared to farmed meat (healthiness, environmental friendliness, ethicality, tastiness, appeal, zoonosis risk), demographics, eating habits, prior knowledge, meat importance.			product perceptions (health, environment, ethics, taste, appeal, zoonosis risk) are the sole significant predictors; conservative tendency negatively impacts initial trial, while left-right orientation is non-significant.
216	La Barbera et al. (2021)	N=194 Italian consumers (122 females, mean age 42.56 years).	Snowball sampling via social media, online questionnaire, Entomophagy Attitude Questionnaire (EAQ), perceived risk measurement, stepwise regression models.	Edible insects (direct entomophagy) and meat from animals raised on insect-based feeds (indirect entomophagy / Processed Animal Proteins - PAP).	EAQ subscales (Disgust/EAQ-D, Interest/EAQ-I, Feed/EAQ-F), perceived risk (probability x seriousness), intention to engage in direct and indirect entomophagy.	Intention to engage in direct and indirect entomophagy.	Yes - Framed around sustainable food production, limiting repercussions of traditional food systems on planet resources, and alternative protein sources.	Perceived risk does not significantly improve the predictive validity of the EAQ; intention to engage in indirect entomophagy is higher than direct entomophagy; beef and pork reared with insects are less accepted than fish and poultry.
217	Hüppe & Zander (2021)	N=84 organic consumers across 9 focus groups (5 in Germany, 4 in Switzerland).	Qualitative exploratory research, semi-structured focus group discussions, thematic qualitative text analysis using MaxQDA.	Organic food processing technologies, focusing on preservation and processing of organic milk (homogenisation, pasteurisation, microfiltration/ESL, UHT) and orange juice (direct juice vs. juice from concentrate, fresh juice, high-pressure processing/HPP, pasteurisation).	Consumers' knowledge, expectations, and attitudes towards food processing technologies, naturalness perception, risk and benefit perceptions, food technology neophobia (FTN).	Consumer preferences, expectations, and attitudes toward organic food processing technologies (qualitative study).	Yes - Framed around environmental degradation, naturalness, sustainable organic value chains, energy/water consumption, and food waste reduction.	Consumers have little knowledge of processing technologies and rely on top-down values and heuristics (e.g., "natural-is-better", fewer processing steps); two consumer groups identified: 'organic traditionalists' (prefer minimal processing, e.g., pasteurised milk, direct juice) and 'organic pragmatics' (accept higher processing levels for convenience and sustainability benefits like reduced food waste or efficient transport, e.g., ESL milk, HPP juice).
218	Ardoin & Prinyawiwatk u (2020)	N=1,005 U.S. adult consumers (Survey 1: n=403; Survey 2: n=602) recruited via online platforms, consumer databases, and LSU campus.	Two online surveys, check-all-that-apply (CATA) format, cumulative logistic regression, Stuart-Maxwell test, McNemar's test for marginal homogeneity.	Foods, beverages, and snacks containing insect protein powder (evaluated across 30 products in 6 categories: candy/snack, beverage, bakery/cereal, meat/poultry/seafood, dairy).	General willingness to try (GWTT), product appropriateness (WTT per product), perceived risks (appearance, cultural/religious beliefs, negative emotions/disgust, nutrition, odour/aroma, price, taste, texture/mouthfeel, safety, social acceptability, unfamiliarity), entomophagy benefit information (EBI).	General willingness to try (GWTT) and product-specific willingness to try (WTT).	Yes - Framed around sustainable alternatives to livestock consumption, feed conversion efficiency, water/land usage, and greenhouse gas emissions.	72% of U.S. consumers were willing to try at least one insect-containing product; most appropriate products were protein/energy bars (65%), chips/snack crackers (61%), and protein shakes (60%), followed by bakery/cereal products and snacks/candy; unfamiliarity, negative emotions (disgust), and sensory expectations (taste/texture) were primary perceived risks; presenting entomophagy benefit information (EBI) significantly increased willingness to try across all products.
219	Li et al. (2021)	N=1,203 participants in Spain.	Semi-structured online questionnaire survey via Qualtrics consumer panels, binary logistic regression models.	General food products / sustainable food products.	Socio-demographic factors, mood states, trust in information sources, risk preferences (Multiple Price List / MPL), risk perceptions (COVID-19 risk, food security risk, financial risk), knowledge regarding COVID-19, experiences, shopping places.	Total food consumption (C), food expenditure (E), and purchase of food with sustainable attributes (S) during the COVID-19 lockdown.	Yes - Framed around global food security threats, sustainable development, and sustainable food availability during the COVID-19 pandemic.	Gender, age, employment status, income, place of residence, mood, household size, risk perceptions, trust in information sources, and risk preferences significantly influenced food consumption, expenditure, and sustainable purchasing behavior during the lockdown.
220	Weir & Sproul (2019)	N=1,043 survey participants recruited via Amazon Mechanical Turk (Mturk) in the United States.	Online survey, discrete choice experiment (DCE), random-parameters mixed logit model, LASSO (least absolute shrinkage and selection operator) L1-regularization for variable selection, cross-validation.	Fresh, farmed Atlantic Salmon fillets with varying attributes (price, country of origin, and GM labels: No Label, Organic, Verified Non-GM, Fed with GM Soy, GM, Both GM).	Choice attributes, information treatments (positive, negative, balanced, control), behavioral measures (risk perception, confidence in risk perception, concern about risks in health and environmental domains), subjective knowledge, domain-specific ambiguity aversion.	Consumer choice of salmon fillets in discrete choice experiment.	Yes - Framed around aquaculture sustainability, wild fish supply plateau, environmental impacts of open-ocean salmon farming, and genetic modification disclosure (National Bioengineered Food Disclosure Standard / NBFDS).	Health and environmental risk perceptions, confidence and concern about potential risks, subjective knowledge, and domain-specific ambiguity aversion are significant predictors of consumer choice; participants prefer Verified Non-GM and dislike Fed-GM fillets; familiarity with GM technology promotes consumption, while risk concerns can push consumers out of the market entirely.
221	Baker, Shin, & Kim (2016)	Study 1: N=221 online marketing panel respondents in	Three experimental studies (Study 1: 2x2 retail setting experiment; Study 2: 2x2	Edible insect food products (Asian spice mix in retail, fried rice dish in	Product image (actual bug vs. powdered/powdered bug), product description (easily	Purchase intention and expected liking.	Yes - Framed around global population growth, food waste, unsustainable mass food production	In retail settings, actual insect images significantly increase risk perceptions and decrease purchase intent; in restaurant settings,

		the US; Study 2: N=200 online marketing panel respondents in the US; Study 3: N=201 online marketing panel respondents in the US.	restaurant setting experiment; Study 3: 2x2 setting x description experiment), MANCOVA, linear regression, MPlus path analysis for mediation.	restaurant).	recognized name vs. scientific/ambiguous name), food consumption setting (retail vs. restaurant), risk perceptions (functional, social, physical, psychological risks), food neophobia, expected liking, purchase intent.		practices, and edible insects as a sustainable, nutritious alternative protein source promoted by the UN.	both images and descriptions influence risk evaluations; vague/ambiguous descriptions are preferred over explicit descriptions in minimizing risk and increasing purchase intention; functional and psychological risks act as key negative mediators on expected liking and purchase intention.
222	Castellini, Savarese, & Graffigna (2021)	N=1,004 Italian citizens representative of the population (stratified sampling by gender, age, profession, center size, geographical area).	Computer Assisted Web Interviewing (CAWI) online survey, descriptive statistics, contingency tables, Pearson's Chi-square, standardized residuals, one-way ANOVA with Bonferroni post-hoc tests.	Certified sustainable food products (labels including Sustainable cleaning, Ecocert, Ecolabel, Cruelty free, FSC, Fairtrade, Friends of sea, Dolphin safe, MSC).	Risk susceptibility to COVID-19 contagion, changes in attitudes after COVID-19 regarding "One Health" dimensions (environmental issues, animal welfare, human health, individual responsibility in society), self-reported consumption of certified sustainable food products (Phase 1, Phase 2, and next 6 months intentions), socio-demographics.	Self-reported consumption and future purchase intentions for certified sustainable food products.	Yes - Framed around the COVID-19 pandemic, the "One Health" perspective connecting human health, animal health, and environmental preservation, and sustainable food consumption.	During Phase 1 of COVID-19, ~30% of Italians frequently consumed certified sustainable food products, and ~20% intended to increase consumption; higher risk susceptibility to contagion significantly associates with higher consumption and intention to buy sustainable foods; COVID-19 increased awareness of environmental issues, animal welfare, and human health, driving future sustainable food consumption intentions, particularly among highly educated consumers.
223	Kaczmarek (2026)	N=290 young, urban, meat-eating adults in Chad (from a recruited sample of n=304 aged 18-34 years).	Cross-sectional online survey, hierarchical OLS regression with HC3-robust standard errors across six hypothesis blocks, dominance analysis (Shapley R^2 decomposition), binary outcome sensitivity analysis (logistic regression, ROC/AUC), Bayesian nested model comparisons (brms and bridgesampling), elastic net regularisation, conditional random forest analysis.	Cultured meat / cultivated meat.	Cultured meat adoption intention (Q30), binary willingness indicator (Q31), socio-demographics, conventional meat purchase priorities and frequency (Q5, Q7.1-Q7.8), knowledge and awareness (Q1-Q3), product beliefs (safety, health, taste, Q10-Q12), affective risk barriers (disgust, risk, unnaturalness, Q13, Q18.1-Q18.5), need-advantage-value fit (Q19-Q21).	Cultured meat adoption intention (Q30) and binary willingness (Q31).	Yes - Framed around global protein security, greenhouse gas emissions, land use, biodiversity impacts, and the sustainable protein transition.	Approximately 52.4% of young urban Chadian adults expressed willingness to try cultured meat; conventional meat purchasing priorities and knowledge blocks showed no significant incremental contribution; affective risk barriers ($\Delta R^2=0.086$, ranking 1st in dominance analysis at 22.8%), product beliefs ($\Delta R^2=0.056$), and value fit ($\Delta R^2=0.039$) were significant predictors; willingness to pay a price premium was the most constrained component of adoption readiness.
224	Mosier (2023)	N=228 US consumers (subsample completing food and trust pathways from the 2016 Natural Marketing Institute LOHAS survey of over 4,000 individuals).	Secondary data analysis using 2016 Natural Marketing Institute (NMI) LOHAS survey data, ordinal logistic regressions, logistic regressions for very important label preferences.	Food and beverage eco-labels / attribute labels (Fairtrade certified, locally grown/local, natural, non-GMO, organically grown, USDA certified organic).	Eco-label preferences / level of importance (Fairtrade certified, local, natural, non-GMO, organically grown, certified organic), trust in three major institutions (US government, corporations, non-profit organizations/NPOs) to protect the environment, demographic characteristics (gender, age, income, education, race, shopping responsibility, political party affiliation, regional location).	Consumer preference / perceived importance of six major food eco-labels.	Yes - Framed around sustainable food products, environmentally friendly products, and eco-label signaling under information asymmetry.	Trust in non-profit organizations is positively associated with expressed preferences for certified Fairtrade and certified organic foods; trust in the US government is a significant explanatory factor for natural labeling preferences; respondents expressing high interest in organic labels indicated trust in corporations; younger and Democrat-leaning respondents are more likely to seek out eco-labels.
225	Bisht & Mamgain (2026)	N=290 urban consumers across five cities in Uttarakhand (Dehradun, Haridwar, Haldwani, Roorkee, Rudrapur), India (from an initial Wave 1 sample of N=400).	Vignette-based, two-wave survey (21-day separation), PLS-SEM using a Type II reflective-formative hierarchical component model, bootstrapping (5,000 resamples), blindfolding, PLSpredict, MICOM invariance, PLS-MGA.	Millets / millet-based food products (hypothetical brand "NutriMillet").	Perceived Circularity of the Millet Brand (PCMB, formative 2nd order with Circular Production and Waste Valorisation [CPWV] and Circular Packaging and Design [CPD] dimensions), Circular Brand Credibility (CBC), Millet Stigma (MS), Health Consciousness (HC), Taste Perception (TP), Environmental Concern (EC).	Purchase intention (PI).	Yes - Framed around linear agricultural models, third of anthropogenic greenhouse gas emissions, soil degradation, freshwater depletion, circular economy (CE), and sustainable food systems.	Perceived circularity reliably establishes brand credibility (fully mediating its effect on purchase intention, with no direct heuristic shortcut); cultural millet stigma independently suppresses purchase intention ($\beta=-0.207$) across income and age demographics; health consciousness is the strongest direct predictor of purchase intention, whereas environmental concern has zero direct effect; consumers pay significantly more attention to packaging circularity than production-level circularity.
226	Rejman et al. (2023)	N=701 adults (N=330 in Warsaw, Poland; N=329 in Brussels, Belgium).	Direct face-to-face paper and pencil interviews (PAPIs), focus group interviews (FGs), descriptive statistics, non-parametric chi-square tests,	Foods with sustainability certificates / quality labels (Euro Leaf, Fairtrade, PDO, PGI, TSG, etc.).	Perception of certified foods, visibility/availability at points of sale, trust in certification labels, importance of food choice	Consumer perceptions, trust, visibility evaluation, and food choice importance of certified sustainable foods.	Yes - Framed around sustainable food systems, European Green Deal, Farm to Fork strategy, and responsible consumption patterns.	44% of Belgian respondents and 33% of Polish respondents consider certified foods to be of better quality; Belgian consumers possess more extensive knowledge and higher trust in certified foods compared to Warsaw

			Kruskal-Wallis tests, Mann-Whitney tests, Cramer's V coefficient.		factors, sociodemographic characteristics.			inhabitants; certificates are moderately important due to consumer confusion from label proliferation, lack of uniformity, greenwashing, limited visibility/availability, and price sensitivity.
227	Lu et al. (2026)	N=25 university students (aged 18–25, from 14 countries) in Amsterdam, the Netherlands.	Qualitative study combining think-aloud protocols and semi-structured interviews in a simulated supermarket setting using a diminished reality prototype (Microsoft HoloLens 2), Framework Method analysis using ATLAS.ti.	Sustainable food products / food items on a mock supermarket shelf (using bio/non-bio certification as a proxy for sustainability across oat milk, pasta sauce, coffee, peanut butter, pasta).	User experience with diminished reality effects (blurring, hiding, desaturation, complete removal), attention, perceived object presence, curiosity, moral reasoning, habitual preferences, motivations and barriers to future adoption (perceived ease of use, perceived usefulness, autonomy, psychological reactance, trust in technology).	Consumer decision-making processes, product consideration set formation, and future adoption intentions (qualitative study).	Yes - Framed around sustainable food choices, supermarket choice architecture, and sustainable consumption goals.	Diminished reality shapes consumer choices by guiding attentional processes (highlighting non-diminished sustainable options), altering perceived object presence, sparking curiosity, and prompting moral reasoning, which interact with habitual preferences; motivations for future adoption include perceived ease of use and usefulness, while barriers include autonomy concerns, potential psychological reactance, and the need for high functional and institutional trust in technology transparency.
228	Sharma et al. (2026)	N=273 participants (aged 18-60 years) in India.	Online self-administered questionnaire survey, Theory of Planned Behaviour (TPB) extension, structural equation modelling (SEM) via IBM AMOS 29, confirmatory factor analysis (CFA), reliability (Cronbach's alpha) and validity (AVE) assessments.	Sustainably packaged food products.	Trust in brand, environmental concern, social influence, personal innovativeness, price value, product label, purchasing attitude, perceived behavioural control (PBC).	Purchase intention of sustainably packaged food.	Yes - Framed around environmental pollution (landfill overloading, groundwater/marine pollution), single-use plastics, and the need for biodegradable/recyclable packaging.	Attitude is the strongest determinant of sustainable food packaging purchase intention among Indian consumers; environmental concern, social influence, product label, and PBC significantly influence positive attitude formation and subsequent purchase intention; brand trust, personal innovativeness, and price value positively but non-significantly influence purchase intention; the insignificance of trust reflects consumer skepticism of greenwashing.
229	Bassignana et al. (2025)	N=3,023 consumers across Italy (n=1,016), Portugal (n=1,001), and Germany (n=1,006).	Cross-national online survey (Computer Assisted Web Interviewing / CAWI), Mann-Whitney U tests, Kruskal-Wallis tests with Dunn's post-hoc comparisons, ordinal logistic regression, Wilcoxon signed-rank test.	Underutilized crops (UCs) and potential UC labels/logos (three prototypes).	UC knowledge, perceived benefits (economic, environmental, social indices), interest in a UC label, willingness to buy UC-labeled products, perceived overlap with Organic/PDO/PGI labels, socio-demographics, food choice motives, trust in UC logo, reading food labels.	Willingness to buy products with a UC logo (ordinal logistic regression) and interest in a UC logo.	Yes - Framed around agrobiodiversity, resilient agroecosystems, climate change adaptation, low-input practices, and sustainable food systems.	Familiarity with UCs is low (only 16.9% heard of them, 8% identified correct definition), but declared knowledge is associated with higher perceived social, environmental, and economic benefits; trust in the UC label is the strongest predictor of willingness to buy, followed by education, ethical concern, and label-reading habits; consumers perceive a potential UC label as overlapping more with the Organic label than with PDO/PGI quality schemes; the third logo prototype was consistently preferred across all three countries.
230	Ung-Pham et al. (2026)	N=630 volunteers (consumers aged 18–40 years) in Spain.	Online experiment, six video interventions (varying format [formal vs. TikTok-style] and content [health, sustainability, both]), questionnaire, ANOVA with Tukey's post-hoc tests, qualitative analysis of remembered information, correspondence analysis.	Bread enriched with high protein content from lentil and quinoa.	Trust in information presented in videos, remembered information (retained terms/topics), hedonic expectations (expected liking), purchase intention, General Health Interest questionnaire, sustainability attitude questionnaire, food neophilia questionnaire.	Trust in information, expected liking, and purchase intention.	Yes - Framed around public health, environmental sustainability, plant-based foods, and transition to sustainable food consumption.	Trust in information is significantly affected by video format and content; consumers trust formal videos (expert-delivered) more than TikTok-style videos (non-expert), especially when videos present both health and sustainability information; specific information about health and sustainability benefits is better retained by consumers watching TikTok videos; product acceptance and purchase intention vary mainly with consumers' attitudes towards sustainability and neophilia.
231	Bryant et al. (2025)	N=34 adult consumers in Swansea, UK.	Qualitative visual focus groups (creative drawing tasks and group discussion), inductive thematic analysis, visual content analysis (incorporating content analysis, semiotics, and iconography).	Nettles (Urtica dioica) as a general food source and nettle-based soluble, powdered supplements.	Familiarity/awareness of nettle consumption, sensory expectations, perceived barriers to consumption, perceptions of processing and carrier ingredients, trust in health claims, preferences for supplement formats.	Acceptability, perceived behavioral control, and behavioral intentions toward consuming nettle-based supplements (qualitative).	Yes - Framed around sustainable food consumption (SFC), climate change, global greenhouse gas (GHG) emissions, responsible production (SDG 12), and the ecological benefits of nettles (e.g., low inputs, use of marginalized land, improving biodiversity).	Consumers had limited awareness of nettles as a food source, with prior painful experiences (being stung) and negative sensory expectations acting as major barriers to consumption; concerns over processing and unfamiliar carrier ingredients reduced consumer trust, leading them to view the powder supplement as 'ultra-processed' and unhealthy; participants showed a strong preference for natural additional ingredients (e.g., potato starch) and transparent labeling to improve confidence; versatility, convenience, and the ability to disguise the flavor heavily influenced format preferences (e.g., capsules or flakes).
232	Puteri et al. (2024)	N=922 potential early adopters of	Online survey, discrete choice experiments (CE) using real	Insect-based meatballs and insect-based crackers.	Product attributes (price, sustainability claims,	Product choice / Willingness to pay (WTP).	Yes - Framed around alternative proteins, environmental sustainability	Identified four consumer segments for each product, three of which showed willingness to

233		insect-based food in Germany.	packaging designs, latent class conditional logit (LCL) analysis.		nutritional information, naturalness claims, trust indicator, insect labelling), socio-demographics, food neophobia / entomophagy experience.		of insect rearing, reducing deforestation from soy/palm oil, and sustainable food labels.	buy; consumers preferred crackers over meatballs; naturalness claims ("100% natural ingredients", "no additives") were the most important attribute across all target segments; sustainability and nutritional info were favored but not top priorities; institutional trust indicators were key for reluctant buyers; insect-specific labelling ('mealworms') was preferred by enthusiasts, while skeptics preferred general 'insects' labelling.
233	Yousefian, Wenninger, & Dittrich (2022)	N=38 middle-class households in the urban center of Bengaluru, India.	Qualitative telephone interviews, snowball sampling, thematic coding analysis using MAXQDA based on Social Practice Theory.	General food consumption / Immune-boosting foods, organic foods, raw vs. cooked vegetables.	Perceptions of food safety, food shopping practices, food preparation/consumption practices, media/sources of information, impacts of COVID-19, cultural influences.	Shifts in food consumption practices / sustainable food transitions (qualitative).	Yes - Framed around sustainable food transitions, food safety, agro-ecological sustainability, environmental pollution from packaging, and pesticide use.	Middle-class consumers mitigate food safety risks (e.g., pesticide concerns, hygiene) by carefully selecting where to shop and avoiding raw vegetables; COVID-19 increased consumption of immune-boosting foods and traditional herbs/spices (e.g., kashaya tea); demand for organic food is hindered by high costs and lack of trust in verification/labeling systems.
234	Mankad & Carter (2025)	N=17 primary carers of infants/toddlers in Australia.	Qualitative study, online semi-structured focus group discussions (FGDs) via WebEx, inductive thematic analysis.	Infant formula containing synthetically derived human lactoferrin (produced via precision fermentation / GM yeast).	Emotions related to formula feeding, general attitudes and perceived risks of novel functional ingredients (precision fermentation), acceptability of human lactoferrin, decision-making criteria for choosing infant formula.	Acceptability / Attitudes towards human lactoferrin in infant formula (qualitative).	Yes - Framed around lower environmental impact, improved animal welfare, reducing reliance on traditional bovid milk extraction, and sustainable functional food ingredients.	Participants were supportive and highly accepting of using precision fermentation-derived human lactoferrin in infant formula; safety was assumed based on high trust in Australia's national regulatory systems; a more "nature-equivalent" formula provided psychological comfort to mothers feeling guilt over not breastfeeding; main concerns were about consistent accessibility, affordability, and availability of the product rather than the GM/synthetic biology technology used; no "yuck factor" or unnaturalness objections were observed.
235	Faber et al. (2024)	N=7,588 adults across 10 European countries (AT, DE, DK, ES, FR, IT, NL, PL, RO, UK).	Web-based survey, Partial Least Squares Structural Equation Modelling (PLS-SEM), Exploratory Factor Analysis.	Plant-based food alternatives (plant-based meat and plant-based cheese alternatives).	Food choice motives (Environment & ethics, Intrinsic product quality), trust towards plant-based alternative proteins, perceived barriers to plant-based food consumption.	Behavioural intentions towards plant-based meat and cheese alternatives.	Yes - Framed around global warming, food security, and the need to shift towards diets lower in animal-based proteins to achieve climate goals.	Environment & ethics motives are positively associated with intentions to consume plant-based meat (in 6/10 countries) and cheese (in 8/10 countries); "Intrinsic product quality" motives were not directly associated with meat alternatives but varied by country; perceived barriers and trust significantly mediate the relationship between food choice motives and behavioral intentions across all 10 countries.
236	Chakrabarti et al. (2026)	N=72,407 households in Italy (data from the Italian Multipurpose Household Survey 2014 and 2019-2021).	Ordered probit model.	Local and organic food products.	Environmental concern, trust in institutions (Italian Parliament, EU Parliament, Municipal govt.) and people, political motivation, health-related behaviors (reading labels, physical activity), pro-environmental behaviors (saving water/electricity, alternative transportation), socio-demographics.	Frequency of purchasing local and organic food products.	Yes - Framed around ecological citizenship, preserving natural resources, reducing carbon footprints, and sustainable agri-food transitions.	Higher levels of environmental concern, trust in institutions (especially municipal, national, and European) and people, other pro-environmental behaviors (e.g., saving water/electricity), and political motivation significantly increase the likelihood of purchasing local and organic food products; the findings confirm the dual role of individuals as consumers and ecological citizens.
237	Hoesterey et al. (2023)	N=305 and N=485 (two waves) in Germany.	Questionnaire study, Structural Equation Modeling (SEM) based on the Technology Acceptance Framework (TAF).	CUBES Circle (a multitrophic interconnected food production system combining hydro- and aquaculture with insect farming).	Intention to accept, outcome efficacy, subjective norm, trust in stakeholders, positive affect, perceived effects, problem perception.	Intention to accept the technology.	Yes - Framed around food security, resource-efficient systems, reducing ecological footprint, and climate change.	Perceived problem-solving efficacy (outcome efficacy), subjective norm, positive affect, and trust in stakeholders were the most important determinants of acceptance intentions; merely drawing attention to current food production problems did not directly increase acceptance.
238	Macready et al. (2025)	N=25,610 respondents from 13 European countries.	Online survey conducted over two years (2019 and 2020), moderation analyses using bootstrapping (PROCESS macro).	Healthy, sustainable, and novel foods (credence attributes).	Motivation (to eat healthily, live sustainably, be innovative), Trust (social trust, beliefs about trustworthiness of food chain actors, overall trust).	Intention to make healthy, sustainable, and novel food choices.	Yes - Framed around the environmental impact of food production (greenhouse gas emissions, freshwater use) and the European Green Deal.	Trust strengthened the relationship between motivation and intention for sustainable and innovative food choices, but not for healthy food choices; for sustainable choices, this relationship was moderated by trust in farmers and retailers (but not manufacturers or authorities).
239	Dong et al. (2025)	N=4,929 consumers (1,939 from China, 2,986 from	Conjoint analysis, choice experiment, mixed logit model, structured questionnaire.	Rice (with attributes: ecological certifications, traceability, food chain	Trust in food chain actors (farmers, processors, third authority, government),	Consumer switching decisions (choice between status quo and new options) / Willingness to	Yes - Framed around carbon emission reduction in food supply chains and shifting from the status quo toward	Ecological certifications are more effective than food chain brands in China, while this reverses in Thailand; abstract trust in

		Thailand).		brands, price).	preferences for ecological certifications, traceability, and food chain brands, price, demographics.	pay (WTP).	sustainable practices.	institutions/government can reinforce the status quo, whereas interpersonal trust in farmers consistently accelerates switching decisions to sustainable alternatives; consumers experience a "trust paradox" during food transitions.
240	Yang et al. (2022)	N=519 (487 valid samples) consumers who had experienced innovative alternative foods in China.	Online survey, structural equation modeling (SEM), confirmatory and exploratory factor analysis, maximum likelihood estimation.	Alternative foods (specifically focusing on insect-based foods and innovative food designs).	Expectation (EXP), Food Innovation Quality (FIQ), Perceived Value (PV), Satisfaction (SAT), Trust (TRU), demographics.	Continuance Intention (CI).	Yes - Framed around environmental degradation from meat-based diets, carbon dioxide reduction, and environmental sustainability of alternative proteins.	Expectation positively associates with Food Innovation Quality and Perceived Value; Food Innovation Quality and Perceived Value positively influence Satisfaction; Satisfaction leads to Trust, which ultimately determines Continuance Intention; Perceived Value does not directly affect Continuance Intention.
241	Faber et al. (2026)	N=10 (focus group) and N=300 (online survey) in Denmark and Austria.	Exploratory sequential mixed-methods design, focus group discussions (FGD), cross-national online survey, ordinal logistic regression.	Legume grass concentrate (LGC) / clover grass-derived protein.	Awareness, food values, attitudes, behaviour and familiarity, appropriateness of product concepts, neophobia score, sociodemographics.	Willingness to try (buy and consume) / Willingness to pay a premium.	Yes - Framed around sustainable protein sources, reducing greenhouse gas emissions, freshwater consumption, and finding alternatives to monoculture dependency (e.g., soy).	Perceptions of LGC were generally positive, with Austrian participants showing greater interest, trust, and perceived safety than Danish participants; higher education and larger household size predicted awareness and acceptance, while older age negatively influenced willingness to try; consumers preferred LGC in savoury applications (bread, cereals, soup) or smoothies over sweet formats; despite moderate willingness to consume, participants were reluctant to pay a premium.
242	Chu et al. (2023)	N=2,422 respondents across 5 provinces (Beijing, Nanchang, Xian, Taiyuan, and Shenyang) in China.	Population-based face-to-face survey, extended Theory of Planned Behavior (TPB), exploratory and confirmatory factor analysis, structural equation modeling.	Sustainable food (environmentally friendly food).	Behavioral Attitude (BA), Subjective Norms (SN), Perceived Behavioral Control (PBC), Perceived Quality (PQ), trust in certification bodies, demographics.	Behavioral Intention (BI) / willingness to pay more for sustainable food.	Yes - Framed around reducing greenhouse gas emissions, land use, water resource depletion, and shifting away from meat-intensive diets.	Behavioral attitude had the most significant positive effect on willingness to pay for sustainable food, followed by subjective norms and perceived quality. Perceived behavioral control (convenience/access) was negatively associated with purchase intention. Consumers expressed the highest level of trust in government certification bodies over international or local organizations.
243	Lucas et al. (2025)	N=224 German-speaking residents in Switzerland.	Online survey, exploratory factor analysis, multiple linear regression analysis (Backward method).	True Cost Accounting for Food (TCAF) / true pricing of food products.	Previous knowledge of TCAF/externalities, trust in TCAF, concerns for the national economy, true price effect on consumption, affordability importance, green trust, food choice motives (e.g., environmental protection, health interest), sociodemographics.	Attitude towards True Cost Accounting for Food (TCAF).	Yes - Framed around uncovering hidden environmental (e.g., biodiversity loss, soil degradation), social, and health costs of food to incentivize sustainable agri-food systems.	Initial knowledge of TCAF was low, but attitudes became positive after information was provided. Trust in TCAF and the true price methodology (green trust) strongly predicted a positive attitude towards TCAF. A sense of environmental protection and a lower emphasis on affordability also drove positive attitudes. Females and flexitarians showed higher trust in TCAF than males and omnivores.
244	Mustapa et al. (2025)	N=1,006 consumers in Spain.	Quantitative online survey, Contingent Valuation Method (CVM), Heckman sample selection model, payment card format, cheap talk and solemn oath scripts.	Animal products (chicken, pork, eggs) derived from insect-fed livestock and poultry.	Sociodemographics (income, education, age), food neophobia, environmental attitudes (ecocentric vs. anthropocentric), attitudes towards insects in animal feed, purchase behavior, informational context.	Purchase decision / Willingness to pay (WTP) premium.	Yes - Framed around reducing greenhouse gas emissions and land use by replacing traditional livestock feed with resource-efficient, sustainable insect meal.	Consumers were willing to pay a 21-31% premium for insect-fed animal products; surprisingly, providing specific information on insect rearing/processing reduced WTP, while omitting additional information yielded the highest premium. Higher education and online shopping increased WTP, whereas higher income, strong anthropocentric attitudes, and neophobia decreased WTP.
245	Aghaei et al. (2026)	N=246 restaurant consumers in Northern Cyprus.	Quantitative survey, partial least squares structural equation modeling (PLS-SEM), purposive non-probability sampling.	Restaurant services / food consumption.	Perceived corporate social responsibility (economic, legal, ethical, philanthropic), consumer trust, perceived consumer effectiveness, socially responsible consumption.	Attitudes toward restaurant food waste reduction / Ethical behavioral intention toward food waste.	Yes - Framed around global food loss, environmental sustainability, resource scarcity, greenhouse gas emissions, and UN Sustainable Development Goal 12.	Perceived consumer effectiveness fully mediates the relationship between perceived CSR and attitudes toward food waste reduction; perceived CSR positively impacts consumer trust, but consumer trust does not significantly affect attitudes; socially responsible consumption does not moderate the attitude-intention link; empowering consumers' perceived impact is crucial for promoting sustainable habits.
246	Nguyen et al. (2025)	N=450 valid survey respondents via online survey in Vietnam (primarily urban consumers).	Quantitative online survey, Partial Least Squares Structural Equation Modeling (PLS-SEM).	Sustainable food (in the context of blockchain-enabled traceability and reviews).	Blockchain reviews, traceability systems, technology trust, environmental information transparency perceptions, sustainable purchase intention, environmental	Sustainable purchase intention.	Yes - Framed around reducing food safety risks and promoting Sustainable Development Goal 12 (responsible production and consumption) through verifiable environmental transparency.	Blockchain reviews and traceability systems significantly increase technology trust and perceptions of environmental information transparency. Perceived transparency directly enhances sustainable purchase intentions. Traceability systems do not directly influence purchase intention without the mediation of

					concern (moderator).			environmental transparency perceptions. Environmental concern did not significantly moderate the relationship between transparency perceptions and purchase intention.
247	Thomas et al. (2025)	N=109 regular pulse consumers in the USA (Michigan).	Sensory evaluation, 9-point Hedonic scale (blind tasting), visual assessment of packaging (10-point scale), sustainability attitudes survey, stepwise regression.	Ready-to-eat (RTE) retort-processed yellow beans and chickpeas packaged in metal cans, glass jars, and plastic pouches.	Sensory liking (overall, appearance, texture, flavor), product quality, convenience, trust in product, perceived packaging sustainability, sustainability attitudes, demographics.	Overall liking / Perceived sustainability and quality.	Yes - Framed around the environmental benefits of pulses (nitrogen-fixing, low greenhouse gas emissions, low water footprint) and addressing consumer misconceptions about packaging sustainability.	Blind tasting revealed metal cans produced the most consistently liked sensory experience across modalities. When assessing unopened packages visually, consumers overwhelmingly rated glass jars as the highest quality, most trustworthy, and most sustainable packaging, while plastic pouches were rated lowest. Age, diet type, and existing sustainability attitudes significantly predicted how consumers rated packaging attributes.
248	Taufik et al. (2023)	N=136 organic supermarket patrons (Study 1); N=750 nationally representative consumers (Study 2) in the Netherlands.	Two quantitative surveys (field setting and online panel), mediation analysis with bootstrapping.	True price food products (where prices incorporate social and environmental externalities).	Perceived 'green value' (environmental impact), social status, remediation beliefs, green trust (trust in true pricing), purchase intention.	Purchase intention of true price food products.	Yes - Framed around true cost accounting to internalize the hidden environmental (climate change, biodiversity loss) and social costs of food production.	Perceiving 'green value' and social status from true price products significantly increases consumer trust in true pricing ("green trust"), which in turn enhances purchase intention. Remediation beliefs (the belief that extra revenue fixes external costs) did not significantly predict purchase intention.
249	Banovic & Grunert (2023)	Study 1: N=308 (Denmark); Study 2: N=300 (Denmark); Study 3: N=3,032 (Denmark, Germany, Poland).	Between-subjects experimental designs (Studies 1 & 2), online surveys, ANOVA, Structural Equation Modeling (SEM) (Study 3).	Precision fermentation technology (novel food ingredient production).	Study 1: Goal framing (natural vs. sustainable). Study 2: Representative heuristic. Study 3: Trust, health/environmental consciousness, neophobia, skepticism, perceived benefits/usefulness, attitude, purchase intention.	Attitude towards precision fermentation / Purchase intention.	Yes - Framed around precision fermentation as a strategy to mitigate environmental footprints, reduce dependence on traditional agriculture, and improve food security.	Framing precision fermentation as "natural" resulted in more positive attitudes than framing it as "sustainable" (Study 1). Highlighting similarity to traditional fermentation (representative heuristic) enhanced acceptance regardless of framing (Study 2). Cross-culturally, trust in manufacturers increased perceived benefits and reduced skepticism/neophobia; perceived usefulness strongly predicted positive attitudes and subsequent purchase intentions (Study 3).
250	De Mesmaeker et al. (2024)	N=98 dietitians from the Flemish Association of Dietitians in Belgium.	Online survey, multivariate linear regression.	Genetically modified organisms (GMOs) and GM foods (biofortified crops).	Knowledge (objective and subjective), perceived risks, perceived benefits, trust in information sources, amount and direction of information.	Willingness-to-recommend (WTR) GM foods.	Yes - Framed around the use of GMOs as a sustainable strategy to address global food and nutrition security challenges, higher yields, and reduced environmental impact.	Dietitians had limited objective knowledge of GMOs. They were highly willing to recommend GMOs that provide nutritional benefits or contribute to sustainable agriculture (76% WTR). Trust in information sources and perceived benefits were the strongest predictors of WTR, while predominant negative information lowered trust and WTR.
251	Muiruri & Rickertsen (2024)	N=4,683 adult respondents from the Norwegian Monitor Survey (data collected pre- and during COVID-19).	Repeated cross-sectional survey, partial proportional odds (PPO) ordinal logit model.	Cultured meat (CM).	Trust (retailers, food authorities, social), support for green parties, food choice motives (naturalness, health, safety, novelty, price, environment, animal welfare), sociodemographics.	Willingness to try (WTT) cultured meat.	Yes - Framed around substituting conventional meat to reduce environmental footprints (methane emissions, deforestation, land degradation) and improve animal welfare.	Higher social trust, trust in food authorities, and support for green parties significantly increased willingness to try (WTT) CM. Consumers emphasizing natural components and food safety were less willing to try CM, while those emphasizing health, novelty, environment, and price were more willing. Young, male, highly educated, urban, non-religious, and non-vegetarian individuals were more willing to try CM.
252	Verdeau & Monnery-Patris (2023)	N=29 users of a social grocery store in Dijon, France (experiencing food insecurity).	Qualitative semi-structured face-to-face interviews, word association task, thematic analysis.	Food in general (exploring the 4 dimensions of sustainable diets).	Food values (health, naturalness, trust/control, solidarity/frugality, preferences), barriers and enablers to acting on values.	Food choice motives and actual food behaviors.	Yes - Framed around the transition to sustainable diets (health, environment, economic, sociocultural) and the barriers faced by low-income populations.	Consumers experiencing food insecurity prioritized health (nutrition), naturalness, trust/control, solidarity/frugality, and taste preferences. The environmental dimension of sustainable diets was rarely mentioned. Financial constraints severely limited their ability to align behaviors with their values, leading to a feeling of disempowerment, though they employed strategies like home cooking and relying on the social grocery store to regain control.
253	Verbeke et al. (2021)	N=398 consumers from Flanders, Belgium (data collected in Dec 2013).	Cross-sectional web-based survey, binary logistic regression.	Cultured meat (framed as 'cultured', 'in-vitro', or 'synthetic' meat).	Unaided awareness, perceived motives (animal welfare, environment, food security), perceived barriers (unnaturalness, lack of trust), framing terminology, sociodemographics.	Willingness to eat cultured meat as a substitute for conventional meat.	Yes - Framed around the potential environmental, animal welfare, and food security benefits of replacing conventional meat with cultured meat.	Initial perceived barriers (primarily perceived unnaturalness and lack of trust) were twice as powerful as perceived motives (e.g., improved animal welfare) in shaping the willingness to eat cultured meat. Males, younger consumers, and non-vegetarians were significantly more likely to adopt cultured meat. The specific

								terminology used ('cultured', 'in-vitro', or 'synthetic') did not significantly affect willingness to eat.
254	Myint Thu Thu et al. (2024)	N=212 respondents from Yangon and Mandalay, Myanmar.	Telephone interviews, Extended Theory of Planned Behaviour (TPB), Structural Equation Modelling (SEM), Confirmatory Factor Analysis (CFA).	Reared crickets (RC) as food.	Attitude (ATT), Subjective Norms (SN), Perceived Behavioural Control (PBC), Perceived Product Quality (PPQ), Consumer Knowledge (CK), Trust in Producers (TP), Trust in Retailers (TR).	Consumption Intention (CI).	Yes - Framed around insect farming requiring less space, water, and capital, emitting fewer greenhouse gases, and aiding Sustainable Development Goals (SDGs) compared to conventional livestock.	83% of respondents were ready to accept reared crickets as food; consumption intention is directly influenced by attitude, perceived behavioural control, and trust in producers. Consumer knowledge of the environmental friendliness of cricket farming has a positive indirect effect on intention, whereas subjective norms, trust in retailers, and perceived product quality do not significantly affect consumption intention.
255	Lin et al. (2026)	N=16 Vietnamese migrants aged ≥40 years in Sydney, Australia.	Qualitative in-person data collection (individual, dyadic, and small group discussions), reflexive thematic analysis.	Sustainable diets / everyday food choices.	Conceptualizations of sustainability, drivers of food choice, trust, dietary acculturation, motivations and barriers to change.	Food choices and practices (qualitative).	Yes - Framed around planetary health, reducing animal meat consumption, minimizing processed food, and adopting circular economic principles.	Participants had limited familiarity with the concept of "sustainable diets," instead associating sustainability primarily with waste reduction and food quality. Food choices were strongly driven by health, quality, and trust in the Australian food system rather than environmental concerns. Motivation to change diets for environmental reasons was low due to skepticism of impact and language barriers.
256	Lin-Hi et al. (2023)	N=966 participants in Germany.	Pre-post intervention design (two-part online questionnaire), structural equation modeling (SEM).	Cultured meat (burgers from a fictitious restaurant).	Organizational trustworthiness, corporate social responsibility (CSR), extrinsic motives, product characteristics, emotional benefits (warm glow).	Consumer acceptance (willingness to buy).	Yes - Framed around cultured meat's potential to substantially reduce the negative externalities (greenhouse gas emissions, environmental footprint) of the global food system.	Organizational trustworthiness and CSR have a significant and positive influence on consumers' willingness to buy cultured meat. Product characteristics and emotional benefits (warm glow) also positively drive acceptance. The attribution of extrinsic motives did not significantly impact trustworthiness or acceptance.
257	Eom & Choy (2024)	Study 1: N=594; Study 2: N=1,469 participants in the United States (MTurk).	Experimental design (control vs. financial motives vs. prosocial motives conditions), repeated-measures mixed-model analyses, mediation analysis.	Cultured meat.	Motives of scientists (financial vs. prosocial), scientific consensus, trust in scientists.	Safety perceptions of cultured meat / Willingness to consume.	Yes - Framed around sustainable meat production as a solution to greenhouse gas emissions and global sustainability challenges.	Scientific consensus on the safety of cultured meat generally increased positive safety perceptions and willingness to consume. However, providing information about scientists' financial motives weakened this consensus effect by undermining trust in the scientists. Information regarding prosocial motives did not significantly alter the scientific consensus effect.
258	Poulis, Theodoridis, & Zacharatos (2026)	N=324 adult social media users who follow sustainability influencers and buy eco-labeled food (national coverage panel).	Quantitative online survey, Partial Least Squares Structural Equation Modeling (PLS-SEM).	Eco-labeled or sustainability-positioned food products.	Influencer perceived credibility, perceived greenwashing, green brand authenticity, brand trust, sociodemographics.	Green purchase intention.	Yes - Framed around the food industry's contribution to greenhouse gas emissions, biodiversity decline, and closing the "attitude-behavior gap" in sustainable food consumption via digital signals.	Influencer credibility acts as a strong positive signal that significantly enhances green brand authenticity, while perceived greenwashing acts as a negative signal that undermines it. Green brand authenticity strongly predicts brand trust, which in turn acts as the primary mechanism (mediator) driving green purchase intentions.
259	Bayer & Kühl (2024)	N=729 meat consumers in Germany.	Quantitative online survey, cluster analysis (Ward's algorithm, K-means), factor analysis (PCA), ANOVA.	Organic animal products (organic meat/husbandry).	Trust in organic animal husbandry (standards, farmers, control system, processing, retailers), socio-demographics, knowledge, attitudes/expectations, information behavior/preferences.	Consumer trust levels (clusters) and attitudes towards organic animal husbandry.	Yes - Framed around sustainable food consumption, animal welfare, and European policies (Green Deal) aiming to expand organic production by 2030 to promote sustainable agriculture.	Three consumer clusters identified: strongly trusting (34.6%), moderately trusting (52.4%), and weakly trusting (13.0%). Highest trust was placed in organic retailers and farmers; least trust in processing and conventional retailers. Strongly trusting consumers preferred emotional information (photos/stories), while others preferred rational, fact-based information to avoid "idyllic" misrepresentations.
260	Ho, Ou, & Vijayan (2023)	N=24 Muslims (Millennials, Gen X, Baby Boomers) in Singapore.	Qualitative online focus group discussions, inductive coding, cognitive miser model framework.	Cultured meat.	Religious beliefs (halal status), trust in stakeholders (MUIS, government), risk perceptions (health, artificiality), benefit perceptions (environment, food security).	Perceptions and acceptance of cultured meat.	Yes - Framed around cultured meat's potential to alleviate future pressure on global food production, use fewer natural resources, and reduce greenhouse gas emissions.	Participants would only consider consuming cultured meat if it is certified halal by recognized religious authorities (MUIS). Trust in the government and MUIS was strong. Like non-Muslims, participants also had risk perceptions regarding health (artificiality) and recognized societal benefits like food security and environmental protection.
261	Shen et al. (2024)	N=570 consumers in Zhengzhou, China (a new first-tier city).	Online questionnaire survey ("WJX" platform), bibliometric analysis (Citespace), structural equation modeling (SEM) via	Plant-based meat products.	Technical security, price rationality, flavor richness, perceived trust, perceived value, purchase intention,	Purchase intention / Consuming behavior.	Yes - Framed around lowering biological carbon emissions, easing agricultural ecological stress, and ensuring sustainable growth of the	Product features (technical security, price rationality, flavor richness) significantly affect consumer purchase intentions and behaviors by shaping perceptual activity (perceived trust

			SPSS and AMOS.		consuming behavior.		food industry compared to animal meat.	and perceived value). Price rationality is the strongest factor for perceived value, while flavor richness and technical security are most critical for perceived trust. Perceived trust and value act as partial mediators.
262	Stranieri et al. (2023)	N=1,139 beef-eating consumers in Italy.	Online survey (snowball sampling via social media), Confirmatory Factor Analysis (CFA), Structural Equation Modeling (SEM) using Lavaan package in R.	Beef (with an institutional environmental eco-label / EU Ecolabel framework).	Intention to buy, attitude, perceived behavioral control (PBC), subjective norms, food shopping habits, environmental concern, institutional trust, individual characteristics (frequency of consumption, age, gender, education, family components).	Intention to purchase eco-labeled beef.	Yes - Framed around livestock greenhouse gas emissions, mitigating climate change, and orienting consumers toward sustainable meat production and consumption (EU Farm to Fork Strategy).	Beef consumers are likely to change habits toward more sustainable beef choices given clear eco-labeling. Alongside standard TPB constructs (attitudes, PBC, subjective norms), institutional trust and food shopping habits strongly predict purchase intentions. Trust positively impacts attitudes, perceived control, and label-checking habits, highlighting the necessity of public trust for sustainable food choices.
263	Grasso & Asioli (2020)	N=106 UK consumers.	Online survey questionnaire, hypothetical ranking experiment, Mixed Logit (ML) model, Latent Class Logit (LCL) model.	Biscuits made with upcycled sunflower flour (defatted sunflower cake by-product).	Price (£0.40 vs. £1.50 per 300g pack), flour type (wheat vs. upcycled sunflower), protein content (source of protein vs. no info), Carbon Trust label (with vs. without label), food neophobia, knowledge of upcycled ingredients, sociodemographics.	Consumer preferences / Willingness to Pay (WTP).	Yes - Framed around valorizing food processing loss and by-products to support a circular bio-economy and reduce the environmental impact of disposal.	85% of consumers had never heard of upcycled food ingredients before the study, but 85% would consider buying them (primarily driven by environmental and food waste reduction motives). On average, consumers preferred biscuits made with wheat flour over upcycled sunflower flour, but valued the Carbon Trust label and protein claims positively. Latent class analysis revealed three consumer segments: traditionalists who strongly reject upcycled flour (49%), price-sensitive consumers, and environmentalists with high WTP for the Carbon Trust label.
264	Gerber et al. (2025)	Volunteers attending a public tasting event in Miami, Florida (n ≈ 100 total attendees, 19 interviewed by researchers).	Ethnographic analysis, semi-structured interviews, field observations, secondary data (media/social media analysis), Grounded Theory.	Cultivated meat (CM) (chicken product from UPSIDE Foods).	Sensory evaluation, political alignment, consumer acceptance, trust, American identity, verisimilitude, government role.	Consumer acceptance / Public perception and willingness to try (qualitative).	Yes - Framed around cultivated meat as a sustainable alternative or complement to conventional livestock farming to reduce environmental impacts, resource demands, and externalities of animal agriculture.	Public tasting of cultivated meat revealed that aligning CM with ethical priorities while meeting sensory and transparency expectations is vital for building consumer trust. Attendees across diverse political backgrounds united against government bans, viewing them as infringements on consumer freedom. Sensory experiences were mixed, with texturing playing a crucial role, and consumers expressed a strong need for more education, evidence-based communication, and proof of ethical/environmental claims.
265	Kovacs et al. (2023)	N=1,120 US consumers (online panel via Dynata).	Web-based survey, discrete choice experiment (DCE), mixed logit model specified in WTP space, Poe combinatorial test, information treatments.	Ground beef hamburger patties (4 lb packages).	Production method (conventional vs. In-vitro/lab-grown meat), Carbon Trust label, organic label, animal welfare label, price, information treatments (neutral, positive, negative, combined), sociodemographics, political orientation, animal welfare attitudes.	Marginal Willingness to Pay (mWTP) / Consumer preferences.	Yes - Framed around reducing intensive farming environmental impacts (greenhouse gas emissions, water scarcity, land requirements, deforestation).	Consumers require a large price discount to accept in-vitro meat (IVM) over conventional meat across all information treatments. Negative information framing leads consumers to demand the largest discount (requiring a $7.34 discount), whereas positive information significantly reduces the required discount ($4.68 discount). Political orientation and animal welfare attitudes affect mWTP for IVM, depending on the information framing.
266	Vastola et al. (2024)	N=233 Italian participants (online convenience sampling via university channels and social media).	Quantitative online survey ("Google Forms"), Extended Protection Motivation Theory (PMT) model including disgust, Partial Least Squares Structural Equation Modeling (PLS-SEM) via SEMinR package in R.	Insect-based foods.	Perceived severity, perceived vulnerability, intrinsic rewards, extrinsic rewards, response efficacy, self-efficacy, response cost, disgust (entomophagy attitude constructs), purchase intention.	Purchase intention for insect-based foods.	Yes - Framed around mitigating the environmental impacts of food production, reducing greenhouse gas emissions, and adopting sustainable healthy diets (European Green Deal and Farm to Fork strategy).	Response efficacy (belief in environmental benefits) and self-efficacy (confidence in incorporating insects) positively and significantly influence purchase intentions for insect-based foods. Conversely, intrinsic rewards and feelings of disgust act as major psychological barriers, negatively impacting purchase intentions. Perceived severity, vulnerability, extrinsic rewards, and response costs were not significant predictors.
267	Jakubowska et al. (2024)	N= 438 Polish respondents (computer-assisted web interviewing (CAWI))	Online interview method (CAWI), Structural Equation Modeling (SEM) using IBM AMOS 29, Confirmatory Factor Analysis (CFA).	Sustainable food (focusing on traditional food products - TFPs).	Personal attitude, subjective norms, perceived behavioral control, consumer knowledge, trust, health consciousness, intention to purchase.	Intention to purchase sustainable food.	Yes - Framed around sustainable food consumption addressing global environmental challenges, mitigating environmental degradation, and promoting health and ethical practices.	Personal attitudes and consumer knowledge are significant positive predictors of sustainable food consumption intentions among Generation Z. Subjective norms, perceived behavioral control, health consciousness, and trust did not significantly affect purchase intentions in this cohort.
268	Dong et al.	N=2,028 consumers	Dual-method design: Choice	Chicken / Food supply	Platform architecture (VBP	Consumer decision certainty /	Yes - Framed around regulated digital	Regulated digital platform architecture acts as

	(2025)	(Study 1 conjoint/choice experiment; Study 2 structural equation modeling).	experiment grounded in prospect theory (Study 1) and Structural Equation Modeling (SEM) based on trust theory (Study 2).	chain products via digital platforms.	platform reference point), variety seeking, institutional trust, individual trust, demand motives (functional, experiential, symbolic), purchase intention.	Choice probability / Purchase intention.	platforms fostering sustainable food consumption, supply chain transparency, and food system integrity.	a powerful reference point that anchors consumer choice and triggers loss aversion, enabling consumers to transition from heuristic simplification to analytical variety seeking. A sequential chain mediation exists where institutional trust in the platform precedes and facilitates individual trust in component suppliers (trust transfer mechanism).
269	Wang, Chung, & Lai (2023)	N=402 valid Green food consumers in China.	On-site questionnaire survey, structural equation modeling (SEM-AMOS 24.0), bootstrapping test, ANOVA test.	Green food (certified by China's Green Food Development Centre).	Chinese traditional cultural values (Confucian collectivism and Daoism), label trust, attitude, willingness to pay (WTP), control variables (education, occupation, income).	Willingness to Pay (WTP) a premium for Green food.	Yes - Framed around ecological agriculture, reducing chemical overuse, protecting the ecological environment, and fostering a sustainable food production system in China.	Trust in the Green food label directly influences willingness to pay and indirectly affects it through consumer attitude. Traditional cultural values (Confucian collectivism and Daoism) significantly influence consumers' attitude and label trust, which in turn drive their WTP for Green food.
270	Banovic et al. (2025)	N=4,361 participants across Denmark, Italy, the UK, and the US.	Online survey platform (Qualtrics) and market research company (Norstat), choice experiment (CE) with mixed logit (MIXL) models in WTP space, latent class logit (LCL) model.	Precision fermentation (PF) milk (compared to conventional cow and plant-based milk).	Type of milk technology, animal welfare information ("100% animal-free"), protein type ("complete protein"), Carbon Trust label, price, information treatments (control, animal welfare, health, environment), socio-demographics, food technology neophobia scale (FTNS), animal welfare and environmental attitudes.	Consumer preferences / Willingness to Pay (WTP) / Policy labeling preferences.	Yes - Framed around sustainable dairy production, mitigating environmental and greenhouse gas emissions, addressing climate change, and meeting societal nutritional and animal welfare demands.	Consumers in all four countries generally show reluctance toward precision fermentation milk compared to conventional cow milk, though plant-based milk faces less resistance. Information framing (animal welfare, health, environment) significantly alters consumer willingness to pay, with effects varying by country and type of benefit. Latent class analysis reveals three consumer segments across countries: strong traditionalists, light traditionalists, and a third group with inconsistent choices. Consumer preferences for conventional dairy naming on PF products vary by country, with the US and Italy exhibiting stronger support for traditional dairy labeling terminology.
271	Kumar, Sharma, & Raj (2026)	N=456 young Indian adults (Varanasi and Lucknow).	Structured questionnaire, purposive sampling, mall intercept method, Structural Equation Modelling (SEM) via AMOS.	Organic food.	Perceived knowledge, environmental awareness, health awareness, trust, attitude, subjective norms, purchase intention.	Purchase intention for organic food.	Yes - Framed around sustainable consumption, enhancing individual well-being, ecological preservation, and sustainable agriculture.	Perceived knowledge, environmental awareness, and health awareness significantly impact consumer attitudes towards organic food, and trust is a key factor in fostering positive attitudes. Attitude and subjective norms have a notable predictive effect on purchase intention.
272	Legendre et al. (2019)	N=337 U.S. respondents (nationwide online marketing pool).	Online survey via Qualtrics, descriptive statistics, Partial Least Squares Structural Equation Modeling (PLS-SEM) using SmartPLS 3.2.7.	Edible insect food products.	Consumer familiarity, media trust, purchase activism, purchase intention, expected liking.	Purchase intention and expected liking of edible insect food products.	Yes - Framed around global food sustainability, protein alternatives, and reducing the environmental damage (greenhouse gas emissions, water usage, soil contamination) of current food systems.	Consumer familiarity with edible insect food products positively builds media trust, which in turn motivates purchase activism regarding sustainability causes. Purchase activism directly triggers positive behavioral outcomes such as expected liking and purchase intention.
273	Sirieix et al. (2012)	N=16 participants across two focus groups (Edinburgh University, Scotland; Newcastle University, England).	Qualitative methodology: two focus groups with individual written exercises followed by group discussions, thematic analysis.	Sustainable food labels (social responsibility/fair trade, carbon emissions, organic, sustainable agriculture, combinations like COGS, local origin, and nutrition labels).	Consumer perceptions, familiarity, trust, preferences, and rejections of single and combined sustainable food labels vs. origin and nutrition labels.	Consumer attitudes, label preferences, and ideal/rejected combinations (qualitative).	Yes - Framed around sustainable consumption, socially responsible practices, reducing carbon footprints, and environmental/social innovations in food production.	Consumers hold positive perceptions of organic and fair-trade labels (like Max Havelaar) but express skepticism toward unfamiliar labels and general corporate greenwashing claims (e.g., Tesco Nurture). Familiarity, trust, and structural fit between combined labels significantly influence consumer acceptance, though certain combinations can detract from a label's perceived value.
274	Happer & Wellesley (2019)	N=270 participants across four countries (UK, US, China, Brazil; 36 focus groups total).	Qualitative focus group analysis (3-staged look at audience reception, fact sheets/information provision).	Meat consumption / livestock products.	Attitudes and beliefs on climate change, meat consumption practices, media engagement, trust/credibility in experts/scientists, government role, individual vs. collective responsibility.	Negotiation of new information and potential attitudinal/behavioral change (qualitative).	Yes - Framed around the livestock sector accounting for 14.5% of greenhouse gas emissions, climate change, deforestation, water depletion, and sustainable development goals (SDGs).	Perceptions and beliefs on climate change are culturally specific but contextualized within individually constructed media environments. Trust in scientists, skepticism towards governments, and individualism vs. collectivism shape responses to information about meat and climate change, with participants in developing nations showing greater openness to reducing meat consumption.
275	Pambo et al. (2018)	N=432 rural participants drawn from two regions in Kenya: Siaya County (common	Personal interviews using structured questionnaires, Principal Component Analysis (PCA), Covariance-based Structural Equation Modeling	Foods from edible insects (FFEI - cricket-flour buns).	Extended Theory of Planned Behaviour (TPB) constructs (attitudes, subjective/personal norms, perceived behavioural	Intentions to consume insect-based foods (FFEI).	Yes - Framed around edible insects as a sustainable, less environmentally burdensome protein source than livestock to reduce environmental pressures.	Rural households show positive intentions to consume insect-based foods, higher among those more familiar with the practice. Perceived behavioural control and self-identity/familiarity interaction exert a stronger

		consumption) and Machakos County (uncommon consumption).	(SEM) via SPSS-AMOS.		control, self-identity and familiarity interaction), trust in information sources, socio-demographics.			influence than general attitudes. Industry and policy sources are more trusted than media.
276	Stubelj et al. (2025)	N=458 adults in Slovenia.	Cross-sectional study using an online closed questionnaire (1KA web application), chi-square tests, Mann-Whitney U test, multiple linear regression, Exploratory and Confirmatory Factor Analysis.	Alternative protein sources (plant-based meat substitutes, insect-based foods, and cultured meat).	Frequency of meat consumption, willingness to include alternative protein sources in diet, ecological awareness, food technology neophobia scale (FTNS), aversions to insect-based foods (Entomophagy Attitude Questionnaire), gender, age, education, intention to reduce meat consumption.	Willingness/intention to include alternative protein sources in the diet / willingness to try cultured meat.	Yes - Framed around climate change threatening global food security, disrupting agriculture, and the need for sustainable diets and plant-based/insect/cultured protein substitutes to reduce environmental footprints.	66.2% of respondents were open to plant-based sources, 23.1% to insect-based foods, and 21% to cultured meat. Acceptance is influenced by demographics, familiarity, meat consumption frequency, and intention to reduce meat. Men and those with prior insect-eating experience were more favorable to insect foods and cultured meat. Multiple linear regression showed that higher food technology neophobia and aversion to eating insects significantly lower interest in trying cultured meat, and women showed lower interest in trying cultured meat. Ecological awareness was not a statistically significant predictor of willingness to try cultured meat.
277	Cankül, Oğuz, & Cankül (2026)	N=502 consumers in Turkey.	Quantitative correlational survey, convenience and snowball sampling, online questionnaire via Google Forms, Exploratory Factor Analysis (EFA), Confirmatory Factor Analysis (CFA), Structural Equation Modeling (SEM).	Edible insects / insect-based foods.	Food neophobia, environmental awareness, healthy eating awareness, feelings of disgust, risk perception, experience, familiarity, attitude, subjective norms, perceived behavioral control.	Intention to consume / level of acceptance of edible insects.	Yes - Framed around sustainable food production and consumption, growing world population, resource use efficiency, and reducing environmental damage compared to traditional livestock farming.	Food neophobia, disgust, risk perception, experience, and familiarity have a negative effect on acceptance, while environmental awareness and healthy eating awareness have a positive effect. Negative experiences and familiarity can reduce rather than increase acceptance.
278	Mancini et al. (2019)	N=165 university students (bachelor's and master's degree) at the University of Pisa, Italy.	Seminar session on entomophagy, two questionnaires before (Q1) and after (Q2) the seminar, sensory evaluation / tasting session of two identical bread samples (one claimed to be supplemented with insect powder), ANOVA, GLM, Wilcoxon rank sum test, structural equation modeling (SEM) via IBM SPSS AMOS 24.0.	Bread supplemented with insect powder ("insect-labelled" vs. control bread).	Sociodemographic attributes, food neophobia (FNS), beliefs towards insect consumption (disgust, distaste, fear, social acceptance), behavioral beliefs, perceived behavioral control (PBC), personal and social insect food rejection, sensory attributes (appearance, odor, flavor, texture, overall liking, probability of future consumption).	Behavioral intention to eat insect-based food / willingness to try (WTT).	Yes - Framed around entomophagy as a sustainable source of nutrients and protein alternatives compared to conventional meat production.	Perceived behavioral control is the main predictor of behavioral intention, followed by neophobia and personal insect food rejection. Attending an informative seminar significantly reduced disgust and improved overall attitudes towards eating insects. Sensory scores showed participants gave "insect-labelled" bread higher scores for flavor, texture, and overall liking, though 28.8% indicated they were less likely to consume it in the future.
279	Wallner et al. (2023)	N=91 consumers in Austria (recruited from 170 respondents to an initial survey).	Product development of pasta sauces, 9-point hedonic scale for acceptance testing, Check-All-That-Apply (CATA) method, penalty analysis, Cochran's Q-test, principal component analysis (PCA), ANOVA and Tukey's HSD test.	Pasta sauces with varying mealworm content (100% mealworm sauce, hybrid 50% mealworm/50% meat sauce, 100% meat sauce).	Liking/acceptance, sensory attributes (appearance, odor, taste, mouthfeel), CATA descriptive attributes, ideal product profile, food neophobia, oral disgust, background lifestyle variables.	Consumer acceptance / overall liking.	Yes - Framed around sustainable protein sources, reducing meat consumption, lowering resources needed, and reducing emissions compared to conventional meat production.	The hybrid sauce (meat and mealworms) received a liking score comparable to the traditional meat sauce (6.9 vs. 6.5, $p > 0.05$), whereas the 100% mealworm sauce was significantly less liked (5.7, $p < 0.05$). CATA analysis showed "fleshy" had the strongest positive impact on liking, while "brownish" and "mushy" had the strongest negative impacts. Results suggest a stepwise substitution of meat rather than a complete and immediate replacement is more effective for consumer acceptance.
280	Thomas et al. (2026)	Study 1: N=475 UK parents of children aged 6–10 years; Study 2: N=453 UK parents of children aged 11–15 years (recruited via Prolific).	Two online cross-sectional studies, structured questionnaires, backward stepwise linear regression models.	Cultivated meat (cell-cultivated meat products / burgers).	Likelihood of trying and regularly consuming cultivated meat (for self, child, and family), familiarity with cultivated meat, acceptance of new technologies/processes, specific acceptance of cultivated meat, Visual Analogue Scale (VAS) attitudes toward cultivated meat/plant-based/conventional beef burgers, food neophobia, adult food disgust, eating behaviors (AEBQ and CEBQ	Likelihood of trying and regularly consuming cultivated meat (self, child, family).	Yes - Framed around climate change challenges, sustainable alternative proteins, and the environmental benefits (greenhouse gas emissions, land and water use reduction) of cultivated meat production.	Across both studies, familiarity with cultivated meat, acceptance of new technologies and processes, and attitudes toward cultivated meat were strong positive generic predictors of trying and regularly consuming cultivated meat. Acceptance of cultivated meat and attitudes toward conventional beef also predicted outcomes, whereas child neophobia and food fussiness were not significant predictors. Child and adult eating behaviors and temperament acted as selective predictors depending on the specific outcome and age group.

					subscales), child temperament (EAS for study 1, EATQR for study 2), fruit/vegetable intake, demographic characteristics.			
281	Chang, Ma, & Chen (2019)	N=316 valid questionnaire responses (out of 408 distributed) in Taichung city, Taiwan.	Purposive sampling, face-to-face survey questionnaire, Confirmatory Factor Analysis (CFA), Structural Equation Modeling (SEM) via SPSS and Amos 21.0.	Insect foods / edible insect products (cricket biscuits, cricket bread, fried grasshoppers, pupae, mealworms, etc.).	Theory of Planned Behavior constructs (attitude, subjective norm, perceived behavioral control), environmental concern, food neophobia, purchase intention.	Purchase intention toward insect foods.	Yes - Framed around global climate change, shortages in global arable land, rapid population growth (9 billion by 2050), and the lower economic and environmental costs/greenhouse gas emissions of insects compared to traditional livestock.	Consumer attitudes, perceived behavioral control, and food neophobia significantly influence purchase intention toward insect foods. Subjective norms and environmental concern did not demonstrate statistically significant direct relationships with purchase intention.
282	Hartmann & Siegrist (2016)	N=104 general population participants in the German-speaking part of Switzerland.	Between-subject experimental design, random assignment to control (traditional corn tortilla chips) vs. experimental condition (cricket flour tortilla chips), pre- and post-tasting questionnaires, multiple linear regression.	Tortilla chips made with cricket flour vs. traditional corn flour tortilla chips.	Food neophobia scale, previous consumption of insects, living contaminants disgust scale, willingness to eat unprocessed insects scale, sociodemographics.	Willingness to eat unprocessed insects.	Yes - Framed around environmental and nutritive reasons why insects might be a more valuable source of protein than conventional animal protein, lower environmental pollution, and reduced water/space requirements.	Exposure to processed insect products (cricket flour chips) significantly increased consumers' willingness to eat unprocessed insects ($\beta = .16$, $p = .048$). Previous consumption of insects ($\beta = .36$) and low food neophobia ($\beta = -.33$) were also strong positive predictors, while living contaminants disgust ($\beta = -.17$) was a negative predictor.
283	Dai et al. (2026)	Study 1: N=395 American participants; Study 2: N=324 American participants (recruited via CloudResearch Connect).	Study 1: Cross-sectional online survey; Study 2: Experimental design manipulating mental accessibility of multicultural experience via essay writing, ANCOVA, parallel mediation analyses, Bayesian structural equation modeling (BSEM).	Novel food technologies (plant-based meat alternatives, insect proteins, cultivated meat, modified atmosphere packaging, urban/vertical farming, aquaponics, gene editing).	Multicultural experience, need for cognitive closure, openness to experience, food technology neophobia, acceptance of novel food technologies.	Food technology neophobia and acceptance of novel food technologies.	Yes - Framed around sustainable food system transitions, food production as a major contributor to environmental degradation (greenhouse gas emissions, freshwater and land use, biodiversity loss), and integrating technology to facilitate pro-environmental outcomes.	Multicultural experience is associated with lower need for cognitive closure and higher openness to experience, which in turn predict lower food technology neophobia and greater acceptance of novel food technologies, respectively. Study 2 confirmed that recalling multicultural experience increased openness to experience, predicting greater acceptance of novel food technologies, and directly reduced food technology neophobia.
284	Shi et al. (2022)	N=2,226 completed questionnaires in China (online survey via WenJuanXing).	Randomized controlled trial (RCT) investigation, online survey, message framing experiment (neutral, gain-oriented, avoidance-oriented), Structural Equation Modeling (SEM).	Artificial meat (plant-based meat and cultured/clean meat).	Regulatory focus (promotion focus, prevention focus), message framing (gain-oriented, avoidance-oriented, neutral), perceived benefits, perceived risks, consumer attitudes toward artificial meat, balance check variables (food involvement, food tech attitude, food neophobia, meat consumption behavior/beliefs).	Consumer attitudes / acceptance toward artificial meat.	Yes - Framed around sustainable development goals (SDGs), ensuring food security, avoiding water wastage, promoting energy efficiency, stopping global warming, and reducing greenhouse gas emissions and resource consumption compared to traditional livestock.	Promotion focus results in higher acceptance of artificial meat by emphasizing perceived benefits and lowering perceived risks, whereas prevention focus results in lower acceptance due to lower benefits and higher risks. Gain-oriented message framing strengthens the relationship between promotion focus and perceived benefits, while avoidance-oriented message framing diminishes the relationship between prevention focus and perceived risk. Prevention focus has a stronger negative effect on cultured meat acceptance, whereas promotion focus has a stronger positive effect on plant-based meat.
285	Egolf, Hartmann, & Siegrist (2019)	N=313 participants from the German-speaking part of Switzerland (quota sampling for gender and age via Respondi AG).	Online survey questionnaire, repeated measurement analysis of variance, full mediation models via PROCESS macro in SPSS.	Seven new food technology applications: Genetically Modified (GM) meat, GM fish, edible nanotechnology coating film, nanotechnology food box, artificial meat, artificial milk, and synthetic food additive (citric acid).	Willingness to eat (WTE) / willingness to drink (WTD), risk perception, benefit perception, state disgust response toward each technology, food disgust sensitivity (Food Disgust Scale - FDS), control variables (general disgust toward meat/fish/milk).	Willingness to eat/drink (WTE/WTD) (acceptance), risk perception, and benefit perception.	Yes - Framed around global population growth, limited environmental resources, the excessive environmental resource consumption of animal-based food products, and the potential of new food technologies to create a more efficient and sustainable system.	Acceptance of gene-technology applications and meat coated with edible nanotechnology film was low, while disgust responses were high. In full mediation models, trait food disgust sensitivity predicted state disgust responses toward each technology, which in turn fully mediated the effects on WTE/WTD, risk perceptions, and benefit perceptions. Higher disgust responses consistently predicted lower acceptance, lower benefit perceptions, and higher risk perceptions across all tested technologies.
286	Sciortino, Sgroi, & Modica (2026)	N=1,500 adult consumers residing in Italy.	Structured online questionnaire, principal component analysis (PCA), binary logit and probit models, marginal effects estimation.	Food products with blockchain-based traceability / value-added food products (functional, clean-label, allergen-free, sustainability-oriented products).	Transparency Index (TRPI) combining trust in blockchain, trace importance, and motivations (safety, sustainability, authenticity, transparency, fraud prevention), education level, income category, purchase frequency, gender, age group.	Willingness to pay (WTP) a price premium for blockchain-traceable food products (binary measure).	Yes - Framed around food sustainability, authenticity, and environmental performance/carbon-related attributes.	The Transparency Index (TRPI) is the strongest predictor of willingness to pay for blockchain-verified products. Consumers who place greater importance on authenticity, ethical/environmental motivations, and trust display higher acceptance, whereas standard socio-demographic characteristics explain little variation. Blockchain mainly reinforces existing consumer expectations regarding product credibility rather than creating new demand.
287	Mohammed	N=236 consumers in	Face-to-face survey	Organic food.	Utilitarian value, hedonic	Consumer purchase intention	Yes - Framed around environmental	Utilitarian and hedonic values, trust, and

	(2021)	Riyadh, Saudi Arabia.	questionnaire outside organic outlets, Partial Least Squares Structural Equation Modeling (PLS-SEM).		value, trust, subjective norms, availability (moderating variable), consumer purchase intention, actual purchasing behavior.	and actual purchasing behavior toward organic food.	sensitivity, ecological matters, and sustainable organic farming.	subjective norms positively affect consumer purchase intention toward organic food, with utilitarian value having the largest effect. Purchase intention positively influences actual purchasing behavior, and availability acts as a significant moderator strengthening the relationship between purchase intention and actual behavior.
288	Asioli et al. (2023)	N=320 consumers in Finland.	Hypothetical choice experiment (CE), online survey, Mixed Logit (ML) model in WTP preference space, Latent Class Logit (LCL) model.	Butter derived from milk produced by cows fed with lipid-rich rapeseed feed vs. regular feed.	Type of feed (regular vs. rapeseed feed), saturated fat content (reduced vs. regular), Carbon Trust label (with vs. without), price, socio-demographics, food neophobia (FNS), health attitudes (HTAQ), pro-environmental attitudes (NEP).	Willingness to pay (WTP) / consumer preferences for butter attributes.	Yes - Framed around livestock greenhouse gas (GHG) emissions, climate mitigation, methane emissions reduction from dairy cows, and sustainable dairy production.	On average, consumers preferred low-price butter produced from cows fed regular feed, with the "reduced saturated fat" claim and Carbon Trust label. However, about one-third of consumers (Segment 1: "Healthier & Environmentalists") were willing to pay a premium price for butter derived from rapeseed feed (+0.99 €/500 g), low saturated fat, and Carbon Trust labeling. Younger and more educated consumers showed stronger preferences for the rapeseed-fed butter.

Abbreviations: FNS: Food Neophobia Scale; FTNS: Food Technology Neophobia Scale; WTP: Willingness to Pay; PLS-SEM: Partial Least Squares Structural Equation Modeling; TPB: Theory of Planned Behavior.
